# Modulation of ABCG2 Transporter Activity by Ko143 Derivatives

**DOI:** 10.1021/acschembio.4c00353

**Published:** 2024-10-24

**Authors:** Qin Yu, Sepehr Dehghani-Ghahnaviyeh, Ali Rasouli, Anna Sadurni, Julia Kowal, Rose Bang-Soerensen, Po-Chao Wen, Melanie Tinzl-Zechner, Rossitza N. Irobalieva, Dongchun Ni, Henning Stahlberg, Karl-Heinz Altmann, Emad Tajkhorshid, Kaspar P. Locher

**Affiliations:** a Institute of Molecular Biology and Biophysics, Department of Biology, ETH Zurich, Zurich 8093, Switzerland; b Theoretical and Computational Biophysics Group, NIH Center for Macromolecular Modeling and Visualization, Beckman Institute for Advanced Science and Technology, Department of Biochemistry, and Center for Biophysics and Quantitative Biology, University of Illinois Urbana−Champaign, Urbana, Illinois 61801, United States; c Institute of Pharmaceutical Sciences, Department of Chemistry and Applied Biosciences, ETH Zurich, Zurich 8093, Switzerland; d Laboratory of Biological Electron Microscopy, Institute of Physics, SB, EPFL, Lausanne 1015, Switzerland

## Abstract

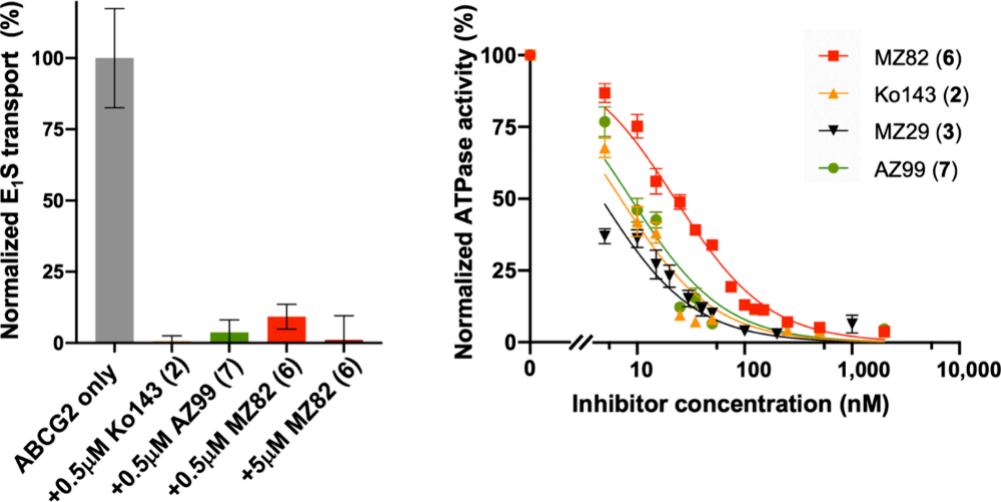

ABCG2 is a multidrug transporter that protects tissues from xenobiotics, affects drug pharmacokinetics, and contributes to multidrug resistance of cancer cells. Here, we present tetracyclic fumitremorgin C analog Ko143 derivatives, evaluate their *in vitro* modulation of purified ABCG2, and report four high-resolution cryo-EM structures and computational analyses to elucidate their interactions with ABCG2. We found that Ko143 derivatives that are based on a ring-opened scaffold no longer inhibit ABCG2-mediated transport activity. In contrast, closed-ring, tetracyclic analogs were highly potent inhibitors. Strikingly, the least potent of these compounds, MZ82, bound deeper into the central ABCG2 cavity than the other inhibitors and it led to partial closure of the transmembrane domains and increased flexibility of the nucleotide-binding domains. Minor structural modifications can thus convert a potent inhibitor into a compound that induces conformational changes in ABCG2 similar to those observed during binding of a substrate. Molecular dynamics simulations and free energy binding calculations further supported the correlation between reduced potency and distinct binding pose of the compounds. We introduce the highly potent inhibitor AZ99 that may exhibit improved *in vivo* stability.

## Introduction

ABCG2, also known as the breast cancer resistance protein (BCRP),^1^ is a clinically relevant ATP-binding cassette (ABC) transporter that limits the cellular penetration of toxins and drugs, thereby promoting physiological detoxification and contributing to multidrug resistance (MDR) in cancer cells.^1−3^ It is constitutively expressed in membranes of numerous tissues and tissue barriers, including the liver, the kidney, or the blood-brain barrier.^4^ By extruding a wide variety of compounds from cells, ABCG2 not only protects tissues from xenobiotics^4^ but also affects the pharmacokinetics of drugs by limiting absorption after oral administration.^5^ When expressed on the apical membrane of renal tissue, ABCG2 has been reported to contribute to uric acid excretion,^6,7^ and its dysfunction caused by genetic polymorphisms is associated with hyperuricemia and hypertension.^8^ When overexpressed in certain cancer cells, ABCG2 contributes to MDR, suggesting that inhibition of ABCG2 may have a therapeutic value in cancer treatment.^1,3,9,10^ ABCG2 was found to be highly expressed in human G3 medulloblastoma, and its inhibition potentiated the response to topotecan, resulting in a > 9-fold increased suppression of tumor cell proliferation.^9^ Because of its biomedical and clinical relevance, extensive efforts have been directed toward understanding the molecular basis of ABCG2 function and its inhibition by small molecules. Structural studies by cryo-EM have revealed the architecture and ligand binding pocket of ABCG2.^11−14^ The transporter is a homodimer, with each half consisting of a transmembrane domain (TMD) and a cytoplasmic nucleotide binding domain (NBD). In an inward-open conformation, two TMDs form a slit-like substrate binding cavity. Substrates bind as single copies, whereas inhibitors can bind as either one or two copies, depending on their size.^12,15^ At the center of the cavity, the side chains of F439 from both TMDs sandwich polycyclic ring systems of substrates. When inhibitors bind, they occupy more space in the cavity and act as a “wedge”, preventing the TMDs from closing and the transporter from proceeding with substrate extrusion. The structure of a catalytically deficient mutant (ABCG2_E211Q_) revealed a closed conformation, with two molecules of ATP bound at the NBDs’ interface and the translocation pathway between the TMDs having collapsed, with no cavity visible at the level of the membrane. This structure represented the conformation of the transporter after substrate release and before reset to an inward-open conformation. Under turnover conditions, ABCG2 revealed two conformational states with bound substrates and nucleotides but differing in the degree of cavity opening. ‘Turnover-2’ state featured semiclosed NBDs and a less accessible substrate cavity compared to the ‘Turnover-1’ state.^16^ While these studies revealed coupled conformational changes within ABCG2 and provided preliminary insight into the discrimination of substrates and inhibitors, it remains poorly understood what determines whether a specific compound acts as a substrate or an inhibitor.

**Scheme 1:**
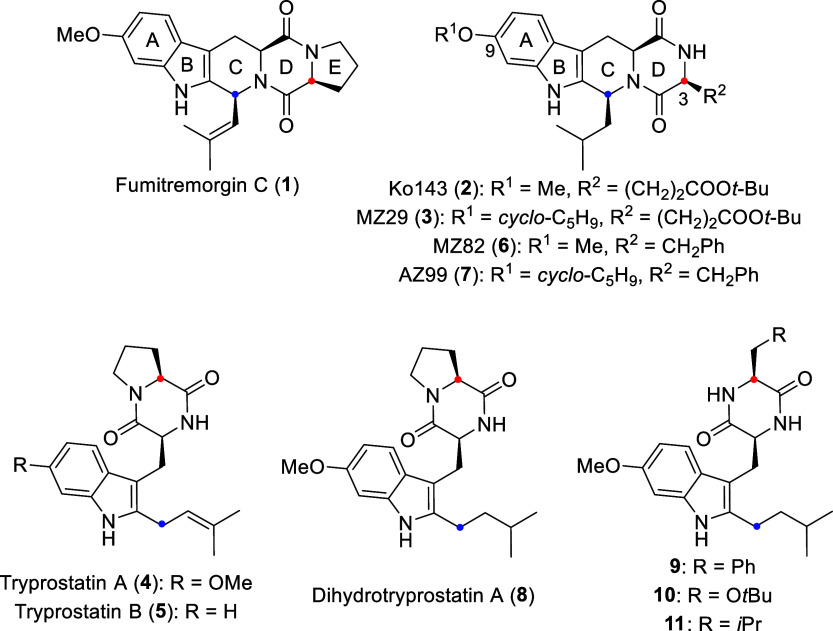
Molecular Structures of the Natural Products Fumitremorgin C (**1**), Tryprostatin A (**4**), and Tryprostatin B (**5**) and of Synthetic Fumitremorgin and Tryprostatin Analogs Investigated in This Study Red and blue dots indicate atom correspondence between tetracyclic and ring-opened structures.

Many compounds have been reported to inhibit ABCG2 *in vitro*, including fumitremorgin C (FTC, **1**) (Scheme 1) and its derivatives,^17−20^ tariquidar and its derivatives,^12^ flavones, chromones, chalcones, quinazolines,^21,22^ and dimeric paliperidone.^23^ However, although inhibition of ABCG2 may increase the efficacy of cancer chemotherapy by undermining the MDR phenotype of cancer cells, no effective and safe inhibitors have been approved for clinical use due to limitations of toxicity, oral availability, or specificity.^9,24,25^ FTC was the first specific inhibitor reported in the literature.^17,18^ It is a tremorgenic mycotoxin that was first isolated from a strain of *Aspergillus fumigatus* obtained by Cole and co-workers in 1977.^26−28^ FTC (**1**) is a pentacyclic indole alkaloid that is derived from L-tryptophan and l-proline as the constituent amino acids, with a prenyl unit providing the link between the indole moiety and a diketopiperazine ring. The neurotoxicity of **1** precludes its use as an MDR-reversal agent *in vivo*,^24^ but several analogs have been developed that are free of neurotoxic effects and are even more potent inhibitors of ABCG2. In particular, Ko143 (**2**) (Scheme 1) was shown to be nontoxic and highly potent.^24^ Derivatives of **2** are therefore potential drug candidates or lead structures for drug discovery, and we have previously reported that MZ29 (**3**) (Scheme 1) is a strong ABCG2 inhibitor *in vitro*.^12^ A drawback of **2** and **3** is the presence of an ester bond in their structures, which can be hydrolyzed by liver or plasma esterases, thus limiting the applicability.^25,29−31^ This problem could be alleviated, at least in part, by replacing the glutamic acid-derived C-3 side chain in Ko143 with a simple methyl group. The corresponding Ko143 analog was shown to be less susceptible to hepatic metabolism *in vitro*(29) and to have an improved *in vivo* pharmacokinetic profile in rats.^30^ More recently, we have shown that the ester-free Ko143 derivative MZ82 (**6**) not only showed greatly improved metabolic stability over Ko143 (**2**) in liver microsomes, but also in mice; the compound was able to penetrate into the brain.^31^

Tryprostatin A (**4**) and tryprostatin B (**5**) (Scheme 1) are seco-analogs of FTC (**1**) that lack the bridging C-ring between the indole unit and the diketopiperazine ring. They were isolated from the marine *Aspergillus fumigatus* strain BM939 by Osada and co-workers in 1996.^32^ Despite its less rigid structure, tryprostatin A (**4**) was found to resensitize ABCG2-overexpressing cancer cells whose growth had been inhibited by the anticancer drug (and ABCG2 substrate) mitoxantrone,^33^ albeit with lower potency than **1**.^32,33^ Tryprostatin A was also found to inhibit the efflux of topotecan and doxorubicin from ABCG2-expressing myeloma cells.^34^ On the other hand, it was unable to reverse ABCG2-mediated resistance to SN-38 in human leukemia cells.^35^ No structure–activity relationship (SAR) data have been published with respect to inhibition of ABCG2 or reversal of ABCG2-mediated MDR by tryprostatin A (**4**); more importantly, the structural basis of the interaction of tryprostatin A (**4**) or related analogs with ABCG2 has not been elucidated.

Here, we report the synthesis of several selected analogs of Ko143 (**2**), including the new tetracyclic structure AZ99 (**7**), the natural fumitremorgin congeners tryprostatin A (**4**) and dihydrotryprostatin A (**8**), and the synthetic tryprostatin A analogs **9**-**11** (Scheme 1). We evaluated the ability of these compounds to inhibit ABCG2-mediated substrate transport in proteoliposomes and compared their activity with that of the previously reported MZ82 (**6**). We also determined high-resolution cryo-EM structures of MZ82 (**6**) and AZ99 (**7**) and analyzed their binding dynamics using computational methods. Our results provide insight into the modulation of human ABCG2 by small molecules and may indicate new avenues for the development of diagnostic or therapeutic ABCG2 modulators and inhibitors.

## Results and Discussion

### Total Synthesis of Tryprostatin A (4) and Fumitremorgin C Analogs

A number of total syntheses of tryprostatin A (**4**) have been reported in the literature,^36−41^ with step numbers for the longest-linear sequence (LLS) from commercial starting materials ranging from 7 to 13 and overall yields between 18% and 55% (for a 7 step LLS).^38^ Previous synthetic work has also been expended on analogs of **4**;^42,43^ however, multidrug-reversal data have not been reported for any of these compounds.

**Scheme 2:**
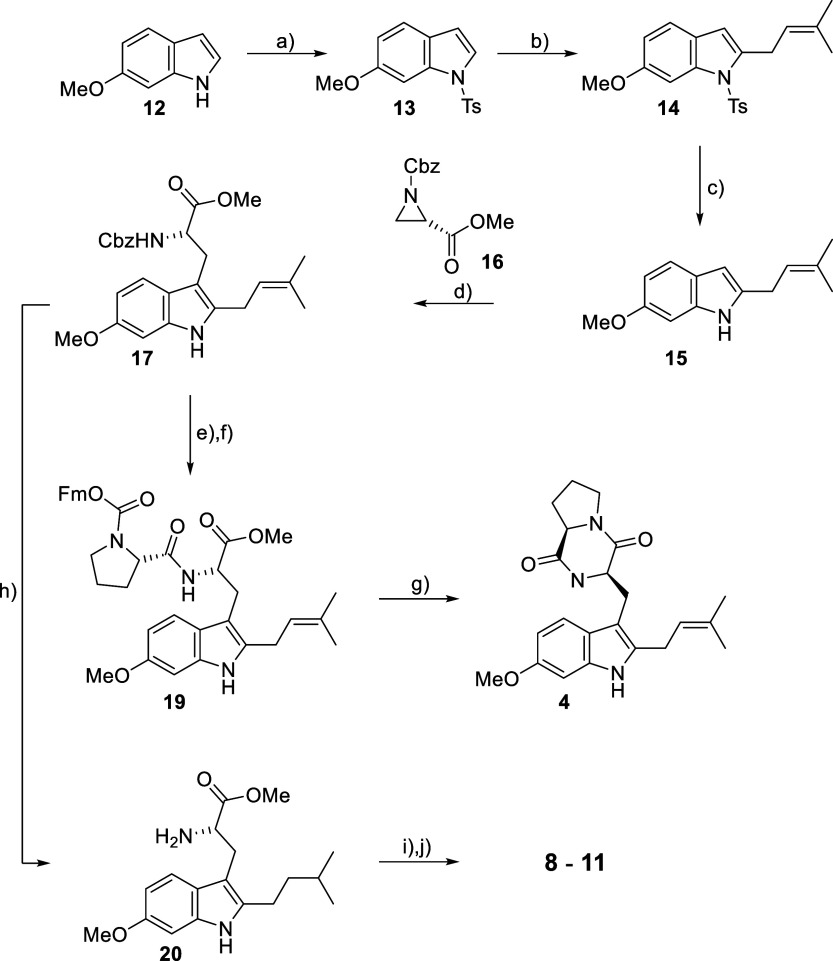
Lewis Acid-Catalyzed Opening of Chiral Aziridine **16** with Prenylated Indole **15** Reagents and conditions: a) TsCl, NaH, THF, rt, 16 h, 80%; b) prenyl bromide, *n*BuLi, THF, −78 °C to rt, 16 h, 78%; c) Mg, MeOH, rt, 1.5 h, 97%; d) Yb(OTf)_3_, CH_2_Cl_2_, rt, 20 h, 96%; e) Et_3_SiH, Pd(OAc)_2_, Et_3_N, CH_2_Cl_2_, rt, 20 h, 90%; f) Fmoc-Pro-OH (**18**), DMTMM, CH_2_Cl_2_, rt, 16 h, 48%; g) piperidine, CH_2_Cl_2_, rt, 1 h, then NH_3_/MeOH, rt, 16 h, 56%; h) H_2_, Pd/C, MeOH, rt, 1 atm, 3 h, 92%; (i) Fmoc-AA–OH, DMTMM, CH_2_Cl_2_, rt, 16 h, 48%; j) piperidine, CH_2_Cl_2_, rt, 1 h, then NH_3_/MeOH, rt, 16 h, 56% (**8**), 28% (**9**), 30% (**10**), 57% (**11**) (three steps each). Ts = tosyl, Cbz = benzyloxycarbonyl, Fm = 9-fluorenylmethyl, DMTMM = 4-(4,6-dimethoxy-1,3,5-triazin-2-yl)-4-methyl-morpholinium chloride.

In the context of the biochemical and structural work reported here, we have developed an alternative access to **4**, which relies on the Lewis acid-catalyzed opening of chiral aziridine **16** with prenylated indole **15** (Scheme 2).^44,45^ The latter was obtained from 6-methoxy indole (**12**) by tosyl protection of the indole nitrogen (TsCl/NaH), prenylation of the 2-position of the indole system with *n*BuLi/prenyl bromide, and tosyl removal with Mg/MeOH^46^ in 40% overall yield. While the prenylation of N-tosyl indole with LDA as the base gave 2-prenyl indole in 57% yield, as has been reported in the literature,^47^ applying these conditions to the 6-methoxy derivative **13** did not yield any product.

The opening of aziridine **16** with **15** in the presence of Yb(OTf)_3_^44^ furnished protected tryptophan derivative **17** in excellent yield (96%). Selective Cbz-cleavage from **17** with Et_3_SiH, Pd(OAc)_2_ and Et_3_N in CH_2_Cl_2_, followed by 4-(4,6-dimethoxy-1,3,5-triazin-2-yl)-4-methyl-morpholinium chloride (DMTMM)^48^-mediated coupling of the ensuing free amino ester with Fmoc-l-proline (**18**) then gave the Fmoc-protected dipeptide ester **19** in 38% overall yield. Treatment of **19** with piperidine in CH_2_Cl_2_ led to Fmoc-cleavage, but the resulting free dipeptide ester did not cyclize to the desired diketopiperazine under these conditions; however, treatment of the cleavage product with NH_3_/MeOH finally furnished **4** in acceptable yield. Overall, tryprostatin A (**4**) was obtained from 6-methoxy indole (**12**) in 7 linear steps in a total yield of 14%.

Analogs **8**-**11** were prepared following the general approach for the successful synthesis of tryprostatin A (**4**), except that the removal of the Cbz-protecting group from **17** was carried out with H_2_ over Pd/C, thus leading to concomitant reduction of the double bond in the isoprenyl side chain (Scheme 2). DMTMM-mediated coupling of the resulting amine **20** with the respective Fmoc-amino acids followed by Fmoc-removal and cyclization then led to analogs **8**-**11** (for details, see Supporting Information).

The synthesis of AZ99 (**7**) followed the same overall approach we had previously developed for other analogs of this type, including Ko143 (**4**), MZ29 (**5**),^12^ and MZ82 (**6**)^28^ (for details, see the Supporting Information).

### Functional Characterization of New Fumitremorgin C Congeners

**Figure 1:**
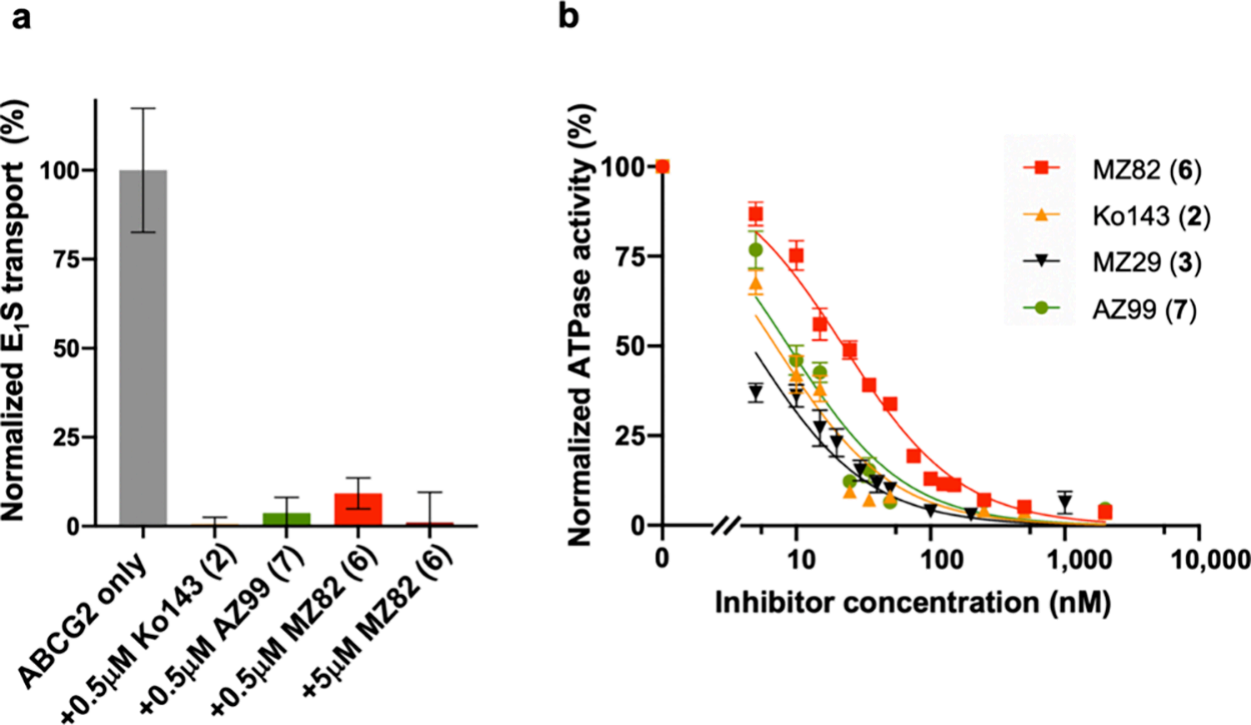
Inhibitory characteristics of tetracyclic Ko143 derivatives. (**a**) Normalized ABCG2-catalyzed E_1_S transport in proteoliposomes without inhibitors or in the presence of 0.5 μM Ko143 (**2**), MZ82 (**6**), AZ99 (**7**) or 5 μM MZ82 (**6**). (**b**) Normalized ATPase activities of nanodisc-reconstituted ABCG2 at varying concentrations of Ko143 (**2**), MZ29 (**3**), MZ82 (**6**) and AZ99 (**7**). The E_1_S transport rate and ATPase activities are normalized relative to the rate/activity in the absence of Ko143 derivatives. Data is presented as mean values ± s.d. Experiments were performed three times independently with the same batch of liposomes or nanodiscs (*n* = 3).

Tryprostatin A (**4**), tryprostatin A analogs **8**-**11,** and Ko143 analogs MZ82 (**6**) and AZ99 (**7**) were tested *in vitro* for their ability to inhibit estrone-3-sulfate (E_1_S)-stimulated ATPase activity of purified human ABCG2 reconstituted in proteoliposomes, using Ko143 (**2**) and MZ29 (**3**) as reference compounds (Supplementary Figures 1b and c). We found that neither **4** nor any of its congeners **8**-**11** significantly reduced the ATPase activity of ABCG2. In contrast, the tetracyclic analogs MZ82 (**6**) and AZ99 (**7**) inhibited ABCG2 ATPase activity at submicromolar concentrations, as did Ko143 (**2**) and MZ29 (**3**). These observations are consistent with previously reported results obtained with other (tetracyclic) Ko143 analogs.^12^ AZ99 (**7**) was also found to fully abolish ABCG2-mediated transport of E_1_S into proteoliposomes at a concentration of 0.5 μM, as did Ko143 (**1**) (Figure 1a). Inhibition of ABCG2-mediated transport of E_1_S into proteoliposomes in the presence of MZ29 (**3**) was shown previously.^12^ Intriguingly, MZ82 (**6**) did not fully inhibit ABCG2-mediated transport of E_1_S at 0.5 μM, but only at 5 μM. We next determined dose–response curves and IC_50_ values for MZ82 (**6**), AZ99 (**7**), Ko143 (**2**) and MZ29 (**3**) by titrating the inhibitors and measuring the ATPase activity of ABCG2 reconstituted in lipid nanodiscs. While nanodiscs are somewhat more artificial than proteoliposomes due to the presence of a membrane scaffold protein, they also contain a patch of lipid bilayer, providing a physiologically more relevant system than detergent micelles. Moreover, nanodiscs can be used for high-resolution structure determination by cryo-EM. Of the four inhibitors tested (**2, 3, 6, 7**), MZ82 (**6**) was the least potent, with an estimated IC_50_ of ∼23 nM (Figure 1b). Precise IC_50_ values could not be determined for Ko143 (**2**), MZ29 (**3**), and AZ99 (**7**) due to the limited sensitivity of the assay system; however, the values are below 10 nM, which is significantly lower than the IC_50_ for MZ82 (**6**). While the replacement of the C-3-substituent in Ko143 with a benzyl group thus led to a weaker inhibitor, the activity could be recovered by simultaneously substituting a larger cyclopentoxy moiety for the C-9-methoxy group. Thus, the new Ko143 analog AZ99 (**7**) is almost equipotent with MZ29 (**3**).

Literature data indicate that the natural product tryprostatin A (**4**) can reverse resistance to mitoxantrone in ABCG2-overexpressing cancer cells but not to SN-38.^32,33,35^ Our data demonstrated that tryprostatin A (**4**) does not inhibit the E_1_S-stimulated ATPase activity of ABCG2, and neither do any of the tryprostatin A analogs **8**-**11**. In contrast, all tetracyclic analogs show significant inhibitory activity. This suggests that the structural rigidity of the compounds with a C-ring is essential for their inhibitory capacity. The fact that tryprostatin A (**4**) has been reported to reverse ABCG2-mediated multidrug resistance to mitoxantrone^33^ at first sight appears to contradict our observed lack of inhibition of ABCG2 by **4**. However, one could speculate that **4** and other Ko143 derivatives with an opened C-ring may still interact with the substrate binding cavity in ABCG2 and compete with mitoxantrone for binding, thereby reducing the expulsion of mitoxantrone from cancer cells. However, the interaction may not be sufficiently strong to exert conformational trapping and prevent the closing of the NBDs of ABCG2, which is essential for ATP hydrolysis. We conclude that the rigid tetracyclic scaffold of Ko143 derivatives matches the shape of the binding cavity of ABCG2, facilitates high affinity binding, and prevents the closure of the NBDs upon ATP binding.

### Cryo-EM Structures of ABCG2 with Ko143 and Three Analogs

**Figure 2:**
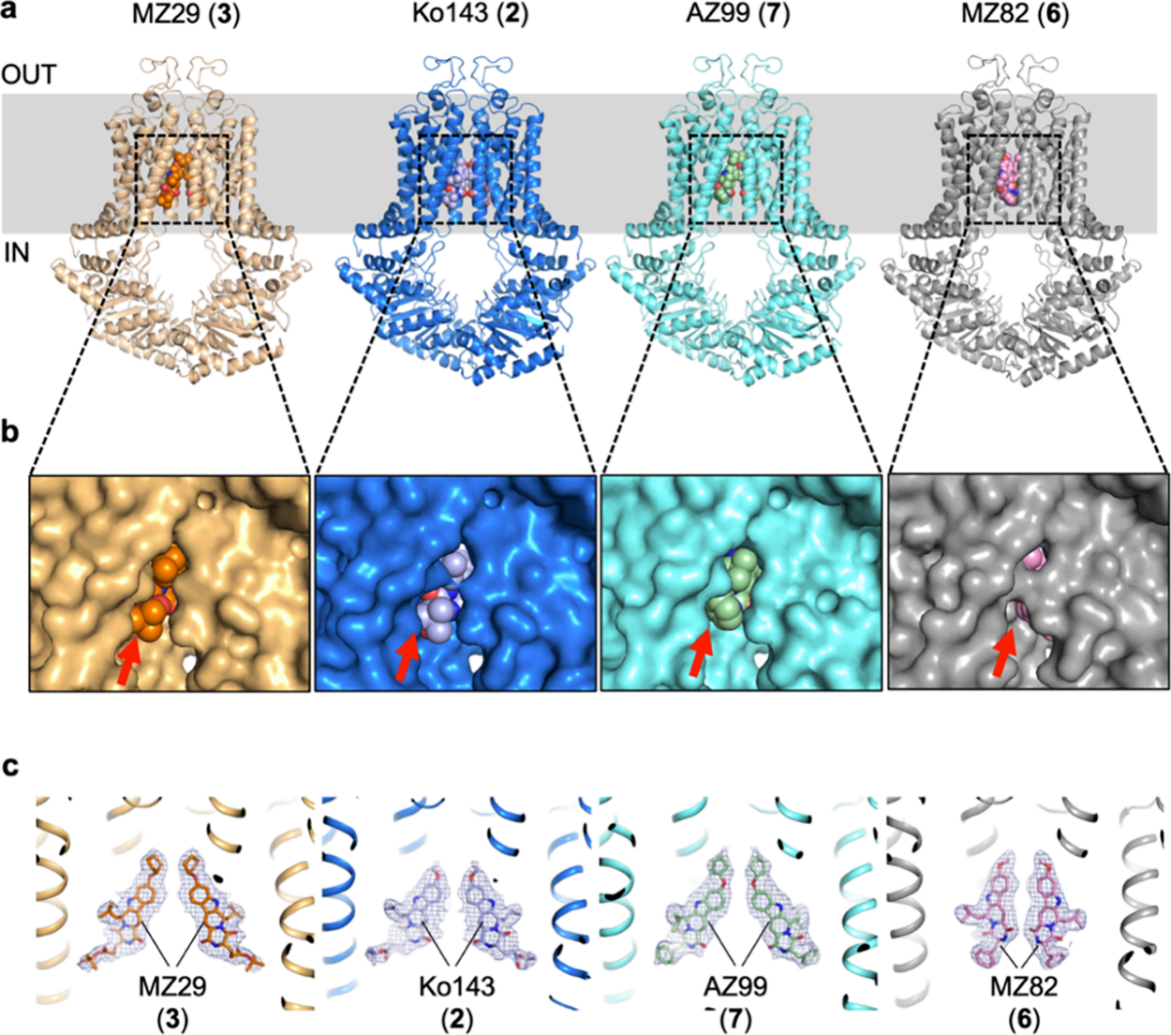
Structures of ABCG2 bound to Ko143 (**2**), MZ29 (**3**), MZ82 (**6**), and AZ99 (**7**). (**a**) Ribbon diagrams of ABCG2, with inhibitors shown as spheres. 5D3-Fab is omitted for clarity. The color scheme is maintained throughout all figures and panels. PDB models used: 8Q7B, 8PY4, 8QCM and 8PXO. (**b**) Close-up of surface representations of side entrance to Cavity 1 viewed from within the membrane. Red arrows point to bound inhibitors. (**c**) EM densities (blue mesh) of bound inhibitors (shown as sticks), with adjacent TM helices of ABCG2 shown as ribbons. The thresholds of the EM maps (EMD-18210, EMD-18016, EMD-18003, EMD-18330) were adjusted such that densities of nearby residues (not shown for clarity) are well-resolved.

To investigate the interaction of ABCG2 with tightly bound tetracyclic Ko143 analogs, we determined high-resolution structures of nanodisc-reconstituted ABCG2 by cryo-EM (Figure 2). The antigen-binding fragment of the ABCG2-specific 5D3 antibody (5D3-Fab), previously shown not to interfere with inhibitor binding,^12^ was also added to increase the size and thereby the resolution of the obtained structures. The structures containing Ko143 (**2**), MZ82 (**6**), and AZ99 (**7**) are new. The structure containing MZ29 (**3**) is based on a previously published data set that has been reprocessed to higher resolution using improved cryo-EM data analysis software.^49^ For MZ29-bound ABCG2, we achieved an overall resolution of 2.56 Å (Figure 2 yellow cartoon, Supplementary Figure 2b, Supplementary Figure 5), which is significantly higher than our previously published structure.^12^ The structures of ABCG2 bound to Ko143 (**2**), MZ82 (**6**), and AZ99 (**7**) were determined at overall resolutions of 3.0 Å, 3.0 Å, and 2.39 Å, respectively (Figure 2, Supplementary Figures 2b, 3, 4, 5). All four structures revealed an inward-open conformation of ABCG2 with two copies of the inhibitors bound to the binding cavity. The observed conformations of ABCG2 bound to Ko143 (**2**), MZ29 (**3**), and AZ99 (**7**) are similar and resemble those previously reported for MZ29 (**3**) at lower resolution and for ABCG2 bound to the tariquidar derivative MB136.^12^ In contrast, the structure of ABCG2 bound to MZ82 (**6**) revealed differences both in the conformation and in the pose of the two copies of the inhibitor. The cryo-EM map for MZ82-bound ABCG2 was obtained at 2.39 Å resolution using C1 symmetry. It revealed two MZ82 (**6**) molecules that were closer together than the corresponding inhibitor pairs of the other tetracyclic analogues. The two MZ82 (**6**) molecules contact each other through the carbonyl oxygens of the scaffold close to the C-3 position (Supplementary Figure 8) whereas the contacts between the two bound molecules of Ko143 (**2**), MZ29 (**3**), and AZ99 (**7**) mainly involve moieties close to the C-9 position. Notably, the conformation of the TMDs in MZ82-bound ABCG2 is different when compared to the other three structures, as evidenced in a narrowing of the cavity entrance and a reduction in its volume (Figure 2b).

### MZ82 Is Lodged Deeper inside the ABCG2 Cavity

**Figure 3:**
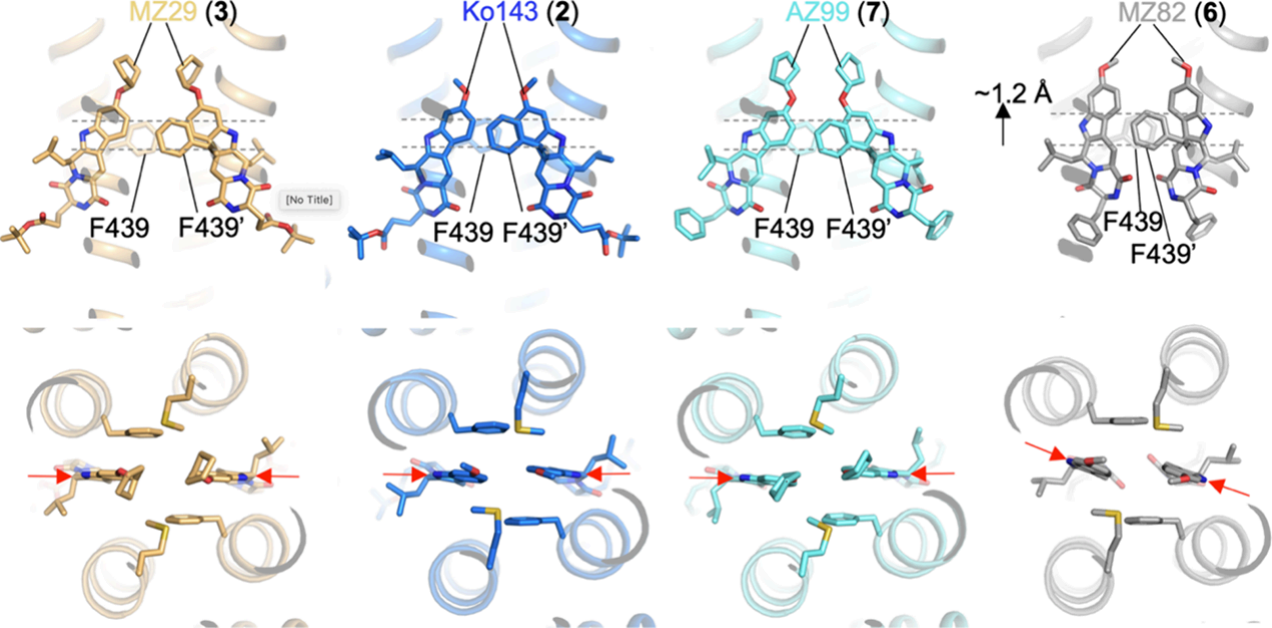
Position of MZ29 (**3**), Ko143 (**2**), AZ99 (**7**), and MZ82 (**6**) in the binding cavity of ABCG2 relative to the two phenylalanines F439 (one from each protomer) that sandwich the polycyclic scaffolds. The view is from the membrane side (top) and the extracellular side (bottom). Selected elements of ABCG2 are shown in ribbon representation, bound inhibitors are shown as sticks. Dashed lines indicate the level of the phenyl rings of F439. A ∼ 1.2 Å shift of the polycyclic scaffold of MZ82 (**6**) relative to AZ99 (**7**), Ko143 (**2**) and MZ29 (**3**) is indicated with a black arrow. The orientation of the scaffolds of the four inhibitors is indicated with red arrows, demonstrating a rotation of MZ82 (**6**) relative to AZ99 (**7**), Ko143 (**2**) and MZ29 (**5**).

Ko143 and its tetracyclic derivatives are all oriented such that the C-9 substituent of the scaffold points toward the apex of the binding cavity, whereas the C-3 substituent points toward its cytoplasmic opening and interacts with the internal cavity surface. The two copies of Ko143 (**2**), MZ29 (**3**), and AZ99 (**7**) in all three cases form a “triangular wedge”, occupying almost the entire cavity volume (Figure 3). The phenyl rings of two F439 side chains, one from each ABCG2 protomer, stack against ring A of the inhibitors. Intriguingly, MZ82 (**6**) is lodged deeper in the cavity than the other three inhibitors, with the polycyclic scaffold shifted by ∼1.2 Å. As a result, ring B instead of ring A is now sandwiched between the F439 side chains (Figure 3 and Supplementary Figure 1a). The two MZ82 (**6**) molecules are rotated ∼15° relative to Ko143 (**2**), MZ29 (**3**), and AZ99 (**7**), resulting in a shape that resembles more of a “rectangular wedge” rather than a “triangular wedge”. This is probably due to the narrow space at the apex of the cavity, forcing MZ82 (**6**) to rotate.

### Conformational Changes in MZ82-Bound ABCG2

**Figure 4:**
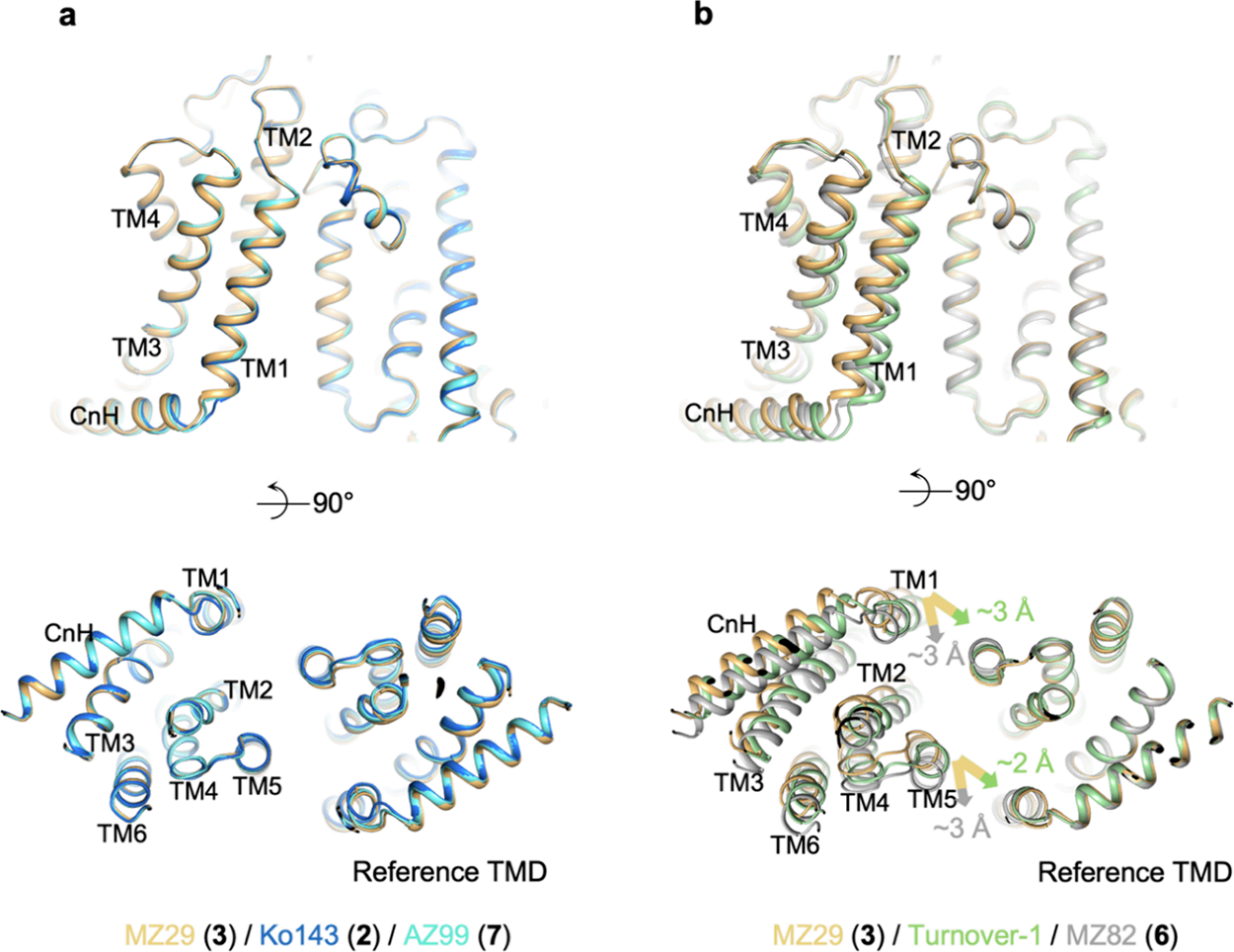
Conformational flexibility of the TMD of MZ82-bound ABCG2 and its comparison with other ABCG2 structures. ABCG2 structures (color coded) are shown upon superimposition of one TMD (right side in panels a and b) to visualize the magnitude of the conformational differences in unaligned TMDs (transmembrane domain). (**a**) Side view (top) and intracellular view (bottom) of the TMDs after superimposition of the ABCG2-MZ29, ABCG2-Ko143 and ABCG2-AZ99 structures. Transmembrane (TM) helices and connecting helices (CnH) are numbered and labeled. NBDs are omitted for clarity. (**b**) Similar to **a** but with the superimposition of ABCG2-MZ29, ABCG2-MZ82 and topotecan Turnover-1 (PDB 7OJH) structures of ABCG2. The color-coded arrows indicate the shift direction of the TMD of MZ82-bound ABCG2 and Turnover-1 relative to that of MZ29-bound ABCG2.

The unique binding pose of MZ82 (**6**) is associated with conformational changes in ABCG2. The TMDs of MZ82-bound ABCG2 adopt a more closed conformation than those observed in the structures containing Ko143 (**2**), MZ29 (**3**), or AZ99 (**7**). To visualize these changes, we superimposed the structures by using one TMD (termed ‘reference’) as an anchor and observing the structural differences to the other TMD (Figure 4). This revealed a very good match between the TMDs of ABCG2 bound to Ko143 (**2**), MZ29 (**3**), and AZ99 (**7**) (Figure 4a), consistent with the similar binding poses of these inhibitors. In contrast, the conformations of the TMDs in MZ82-bound ABCG2 showed pronounced differences (Figure 4b). Transmembrane helices TM1 and TM5 are shifted by 2–3 Å toward the opposite TMD, resulting in partial closure of the binding cavity. The resulting conformation is intermediate between a fully inward-open state and the ‘Turnover-1’ state, but without bound ATP (Figure 4b, green cartoon). This suggests that ABCG2 may initiate conformational changes associated with its transport cycle in the presence of molecules that are inhibitory but compatible with a certain degree of cavity closure.

### Free Energy Calculation of Ligand Binding Affinity

**Figure 5:**
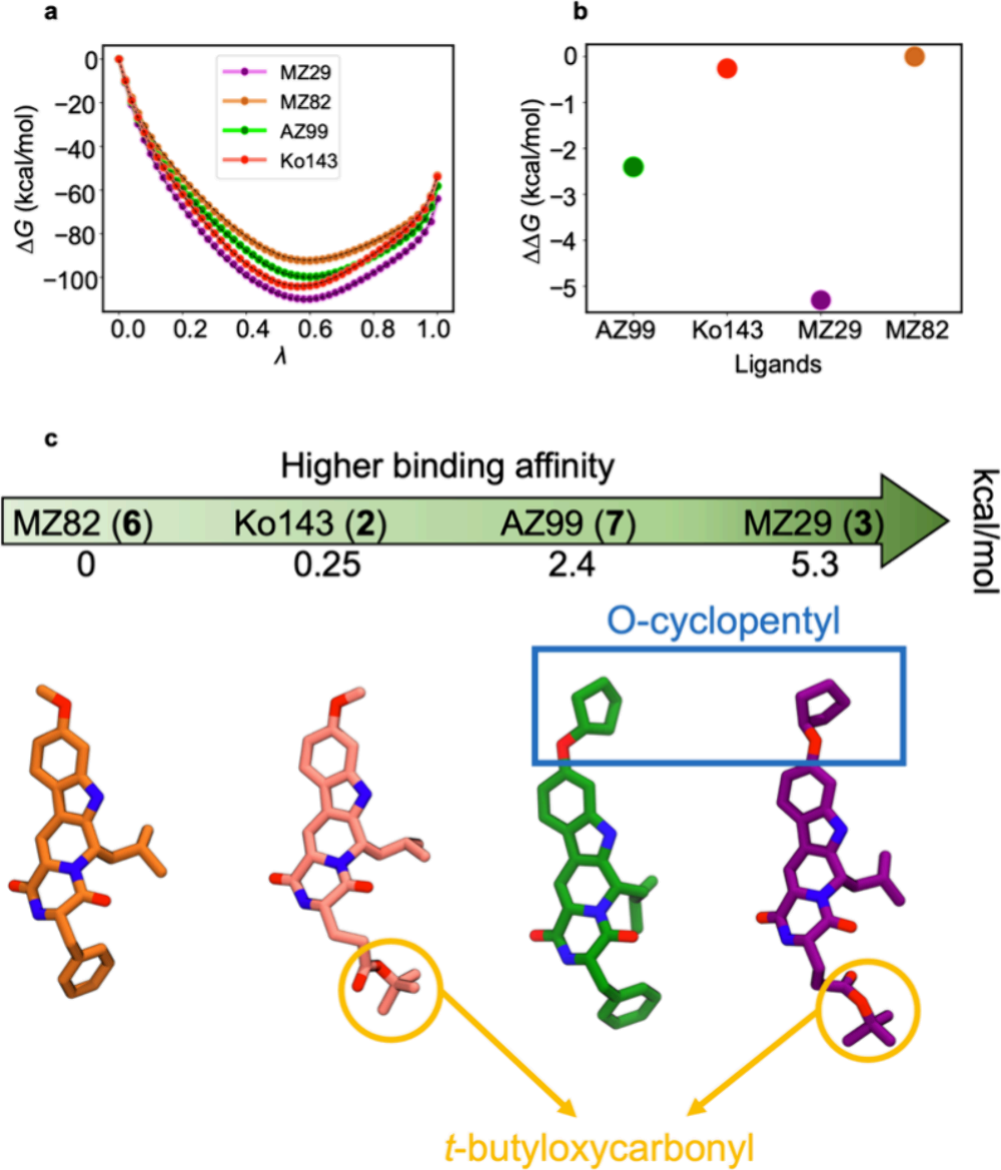
Binding free energies of the inhibitors Ko143 (**2**), MZ29 (**3**), AZ99 (**7**), and MZ82 (**6**), to the binding pocket of ABCG2. **a**. The free energies of binding (Δ*G*) from λ = 0 (ligand-free) to λ = 1 (inhibitor-bound) are shown in purple, brown, green, and salmon, respectively. The forward direction FEP results are shown using the colored symbols, whereas black dashed lines represent the backward FEP (from λ = 1 to λ = 0) simulations. **b**. The relative binding free energies (ΔΔ*G*) of a single inhibitor with respect to MZ82 (**6**). MZ29 (**3**) and MZ82 (**6**) have the highest and lowest binding affinities, respectively. **c**. Important functional groups, namely O-cyclopentyl and t-butyloxycarbonyl, in binding free energies of the four inhibitors.

To quantify the binding affinity of the ABCG2 inhibitors Ko143 (**2**), MZ29 (**3**), AZ99 (**7**), and MZ82 (**6**), we employed Free Energy Perturbation (FEP), a method based on the principles of statistical thermodynamics, to calculate the relative binding free energies for these compounds. The free energy changes (Δ*G*) from λ = 0 (ligand-free) to λ = 1 (inhibitor-bound) were calculated for each compound. Error bars were estimated for each λ, employing the Bennett Acceptance Ratio (BAR) method.^50^ The small size of the estimated errors indicated convergence in the molecular dynamics simulations. Relative to MZ82 (**6**), the calculated binding affinity of Ko143 (**2**) is 0.25 kcal/mol higher; for AZ99 (**7**), it is 2.40 kcal/mol higher; and for MZ29 (**3**), it is 5.30 kcal/mol higher. This indicates tighter binding of Ko143 (**2**), MZ29 (**3**), AZ99 (**7**) compared to MZ82 (**6**), with MZ29 (**3**) having the highest and MZ82 (**6**) the lowest affinity among the four inhibitors (Figure 5a, 5b, and 5c). Our structural findings are in line with the functional observations that MZ29 (**3**) is the most potent and MZ82 (**6**) is the least potent inhibitor of ABCG2. The calculated relative binding free energies suggest that a cyclopentoxy group at the C-9 position and a *tert*-butyloxycarbonylethyl group at the C-3 position both increase the binding affinity relative to a methoxy group at C-9 and a benzyl group at C-3, with the cyclopentoxy group having a stronger effect. Of the four tetracylic ABCG2 inhibitors investigated here, AZ99 (**7**) stands out, as it combines high inhibitory potency (in particular, higher than for MZ82 (**6**)) with the absence of an ester group, which represents a metabolic weak spot of Ko143.^28−30^ Our cryo-EM structures confirm that AZ99 (**7**) binds to ABCG2 in a similar way as Ko143 (**2**). While the calculated binding affinity by FEP simulations is higher for AZ99 (**7**) than for Ko143 (**2**), the experimentally determined inhibition of ABCG2 by AZ99 (**7**) is slightly lower. (Figure 1 a, b). This minor discrepancy between the experimental and computational results might be because FEP calculations only consider the binding step for each inhibitor and ignore other steps of the transport or ATPase cycle of ABCG2. In addition, ABCG2 adopts a different conformation in the presence of MZ82 (**6**) compared to the conformation in the presence of other analogs. This difference in conformation could potentially affect the estimated binding values for MZ82 (**6**).

### MZ82 (**6**) as an Inhibitor and a Substrate

Specific hydrogen bonds with amino acid side chains on the inner surface of the binding cavity appear essential for the binding of the tetracyclic Ko143 (**2**). MZ29 (**3**) forms two hydrogen bonds with ABCG2, one between the side chain hydroxyl of T435 and the oxygen atom of the cyclopentyloxy substituent at the C-9 position, and another between N436 and the NH group of the indole ring (Supplementary Figure 8b). AZ99 (**7**) and Ko143 (**2**) each form only one hydrogen bond involving the NH group of the indole ring and the oxygen of the side chain carboxamide group of N436 (Supplementary Figure 8a and c). The analogous contact in MZ82 (**6**) is weaker, as indicated by a longer distance, which may explain its lower binding affinity and reduced inhibitory potency (Supplementary Figure 8d).

One of the most intriguing observations of this study is the fact that MZ82 (**6**) combines the properties of both an inhibitor and of a substrate. On one hand, it inhibits the transport and ATPase activities of ABCG2, albeit with lower potency than the other tetracyclic Ko143 analogs investigated. On the other hand, it captures ABCG2 in an intermediate conformation between a fully inward-open and the previously observed ‘Turnover-1’ state, which is thought to represent an early stage of a productive transport cycle. This is possible because the two MZ82 molecules are lodged deeper inside the binding cavity and are closer to the 2-fold molecular symmetry axis of ABCG2 than the other three compounds. Extrapolating from these findings, it is tempting to speculate that the smaller the (hydrophobic) substituent groups at the C-9 and C-3 positions of the Ko143 scaffold, the more likely it is that the resulting compounds exhibit characteristics of substrates rather than inhibitors. We propose that among compounds that do fit and interact with the ABCG2 substrate binding cavity, there exists a continuum from substrate to inhibitor structures rather than a sharp distinction between these categories. MZ82 (**6**) is an example of a compound that appears to be closer to the middle of this substrate-inhibitor continuum than the other Ko143 derivatives studied. This concept, which is unprecedented to our knowledge, may extend to other MDR ABC transporters.

## Conclusions

Our results show that computational studies can help evaluate and analyze the interactions of compounds with multidrug transporters. A key prerequisite for such analyses is high-resolution structural insight as provided by our cryo-EM analyses. The example of MZ82 (**6**) is a stark reminder that extrapolating drug-transporter interactions from previous inhibitor-bound states may be misleading, and that experimental structure determination is essential for meaningful computational analyses.

Our findings not only enhance our understanding of small molecule modulation of ABCG2 function but also offer a valuable toolbox for rational structure-based drug design and *in silico* drug discovery of ABCG2 inhibitors in the future. AZ99 (**7**) is a potent ABCG2 inhibitor with structural features (absence of an ester bond) that make it suitable and interesting for *in vivo* investigations, especially in light of promising *in vivo* activity of the inherently less potent MZ82 (**6**).^28^ The compound may have potential for both diagnostic and therapeutic applications.

## Data Availability

Atomic coordinates of the all ABCG2-Fab-MZ29, ABCG2-Fab-Ko143, ABCG2-Fab-MZ82 and ABCG2-Fab-AZ99 models have been deposited in The Protein Data Bank (PDB) under accession IDs: 8Q7B, 8PY4, 8QCM and 8PXO, respectively. The cryo-EM postprocessed, masked maps and half-maps have been deposited in the Electron Microscopy Data Bank (EMDB) under accession number EMD-18210, EMD-18016, EMD-18330 and EMD-18003. All other data are available from the corresponding author upon request.

## Supporting Information Available

Figure S1: Functional characterization of new Ko143 derivatives; Figure S2: Sample preparation and cryo-EM maps; Figures S3–S6: Cryo-EM data processing and model validation of ABCG2-Ko143-Fab, ABCG2-AZ99-Fab, ABCG2-MZ29-Fab, and ABCG2-MZ82-Fab; Figure S7: The comparison of Fab-ABCG2-MZ82 structure with inhibitor bound structures and with topotecan bound structures; Figure S8: Interactions of inhibitors and ABCG2 in Cavity 1; Method Section; NMR data of compounds (PDF)
