# Supplementary material for: Modulation of ABCG2 Transporter Activity by Ko143 Derivatives
Source: ACS Chem Biol. 2024 Oct 24;19(11):2304–13. doi: 10.1021/acschembio.4c00353 (PMC11574751; doi:10.1021/acschembio.4c00353)
Supplement: Supplementary file 1 — cb4c00353_si_001.pdf [file cb4c00353_si_001.pdf]

Supplementary Material

Modulation of ABCG2 Transporter Activity by Ko143 Derivatives

Qin Yu‡[a], Sepehr Dehghani-Ghahnaviyeh‡[b], Ali Rasouli‡[b], Anna Sadurni‡[c], Julia Kowal‡[a], Rose Bang-Soerensen[a], Po-Chao Wen[b], Melanie Tinzl-Zechner [c], Rossitza N. Irobalieva[a], Dongchun Ni[d], Henning Stahlberg[d], Karl-Heinz Altmann*[c], Emad Tajkhorshid*[b], Kaspar P. Locher *[a]

[a] Institute of Molecular Biology and Biophysics, Department of Biology, ETH Zurich, 8093, Zurich, Switzerland

E-mail: locher@mol.biol.ethz.ch

[b] Theoretical and Computational Biophysics Group, NIH Center for Macromolecular Modeling and Visualization, Beckman Institute for Advanced Science and Technology, Department of Biochemistry, and Center for Biophysics and Quantitative Biology, University of Illinois Urbana-Champaign, Urbana, IL 61801, USA

E-mail: emad@illinois.edu

[c] Institute of Pharmaceutical Sciences, Department of Chemistry and Applied Biosciences, ETH Zurich, 8093, Zurich, Switzerland

E-mail: karl-heinz.altmann@pharma.ethz.ch

[d] Laboratory of Biological Electron Microscopy, Institute of Physics, SB, EPFL, Lausanne, Switzerland

‡ Equal contribution

Supplementary Figures

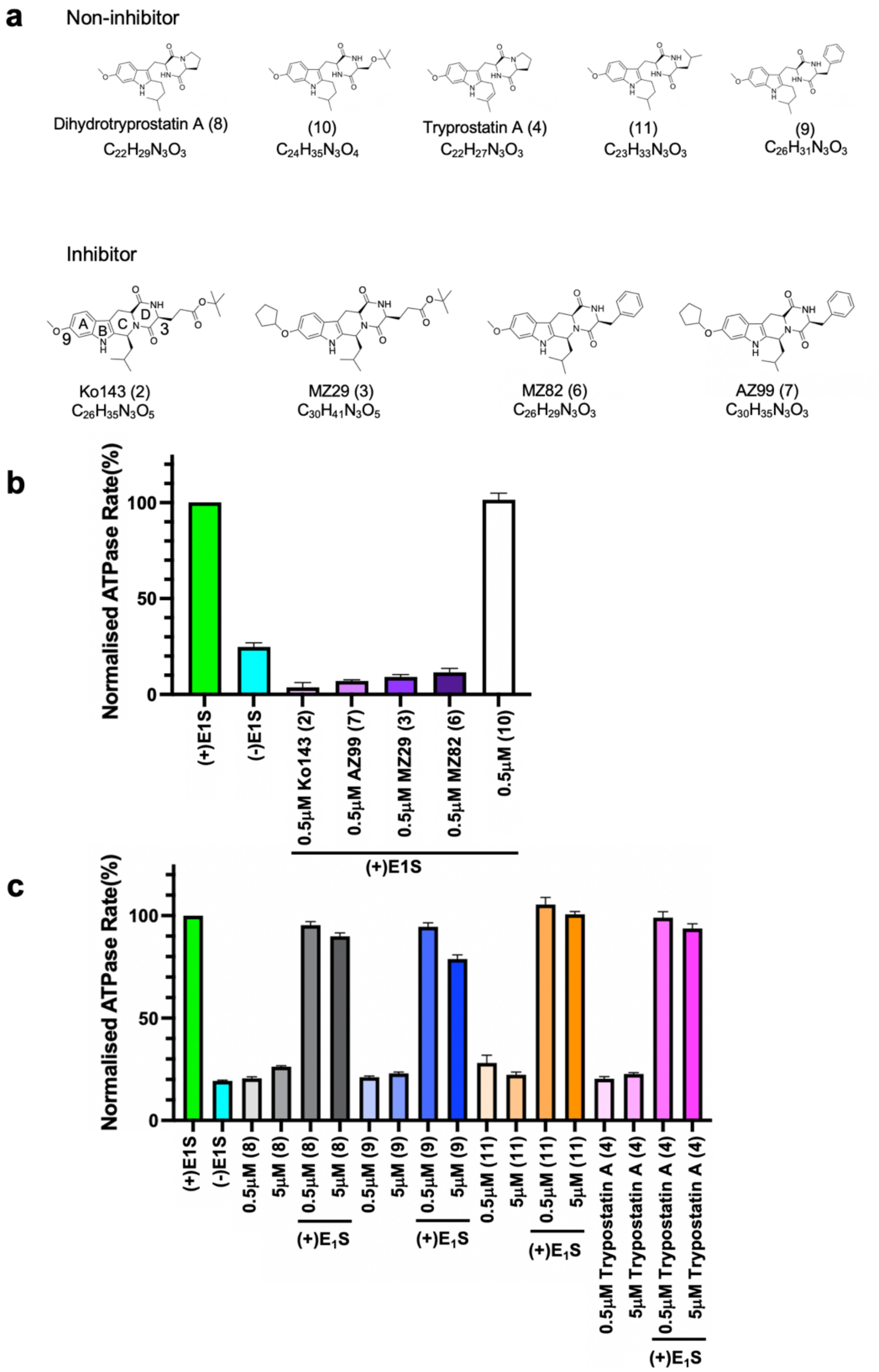

Supplementary Figure 1. Functional characterisation of new Ko143 derivatives **a** Chemical structures of open-ring scaffold and closed-ring Ko143 derivatives. The C-3 and C-9 positions and rings A–D of the Ko143 scaffold are labeled. The chemical formulae are indicated. **b** Normalized ATPase activity of liposome-reconstituted ABCG2 in the presence or absence of 50 μM E_1S, and in the presence of 0.5 μM Ko143, AZ99, MZ29, MZ82 and 10. ATPase rate is normalized with respect to the activity of ABCG2 liposome in presence of 50 μM E_1S. The ATPase rate of ABCG2 liposome in presence of 50 μM E_1S is set to 100%. **c** Normalized ATPase activity of liposome-reconstituted ABCG2 in the presence or absence of 50 μM E_1S, and in the presence or absence of 8, 9, 11 and 4. The ATPase rate of ABCG2 liposome in presence of 50 μM E_1S is set to 100%.

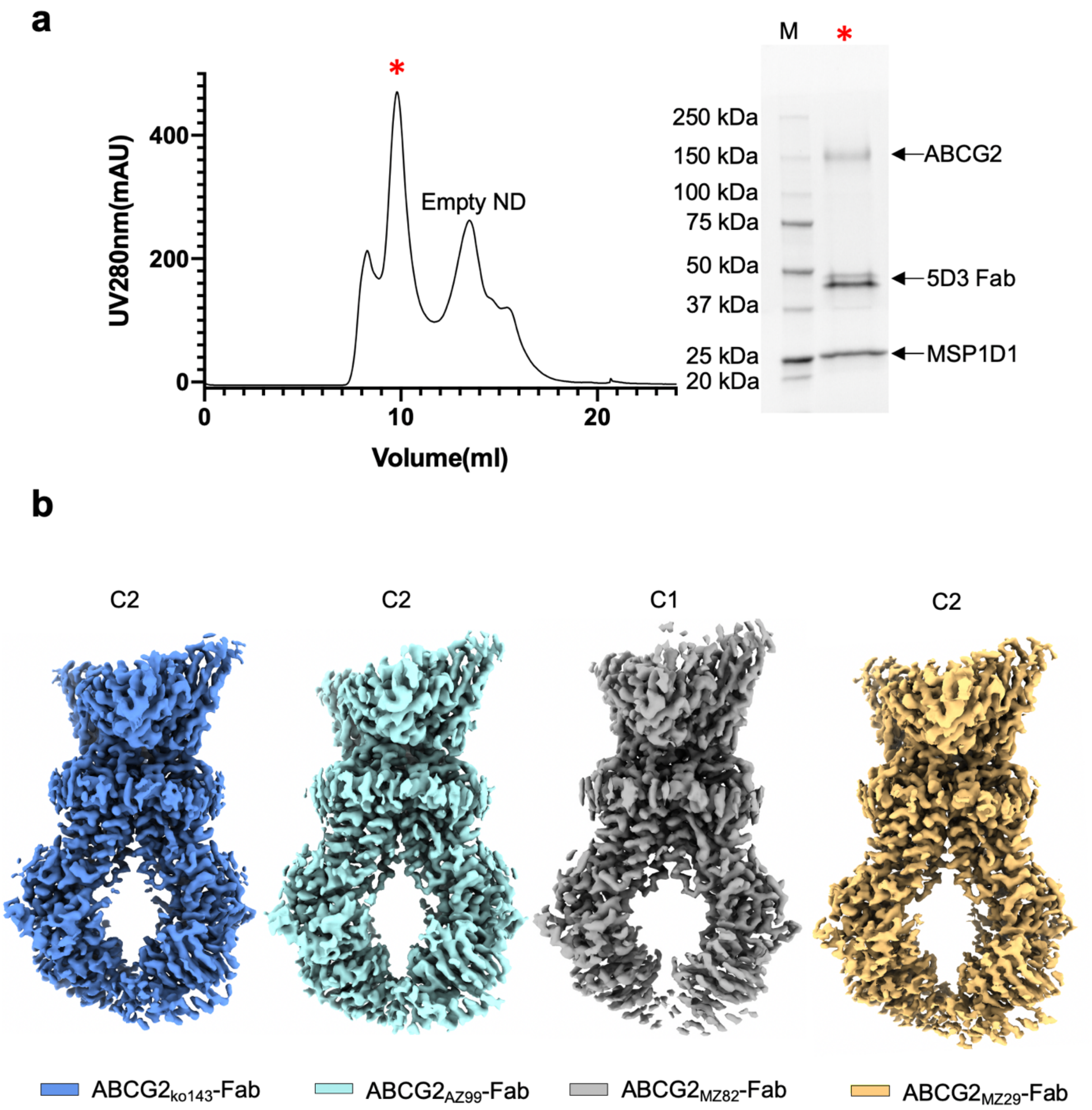

Supplementary Figure 2. Sample preparation and cryo-EM maps **a** Left, the preparative gel filtration profile of nanodisc-reconstituted ABCG2 with Fab. Empty ND indicates the peak for empty nanodisc. Right, representative SDS–PAGE analysis of main peak obtained in gel filtration. No reducing agent was used and ABCG2 runs as a disulfide-linked dimer. M shows marker proteins, with masses indicated on the left. The red asterisk shows the peak fraction analyzed in the gel and used for EM grid preparation. Source data are provided in the related Source Data file. Experiment was repeated 3 times with similar results. **b** Cryo-EM maps of ABCG2-Fab with inhibitors (Ko143, AZ99, MZ82, MZ29). The color code is shown below.

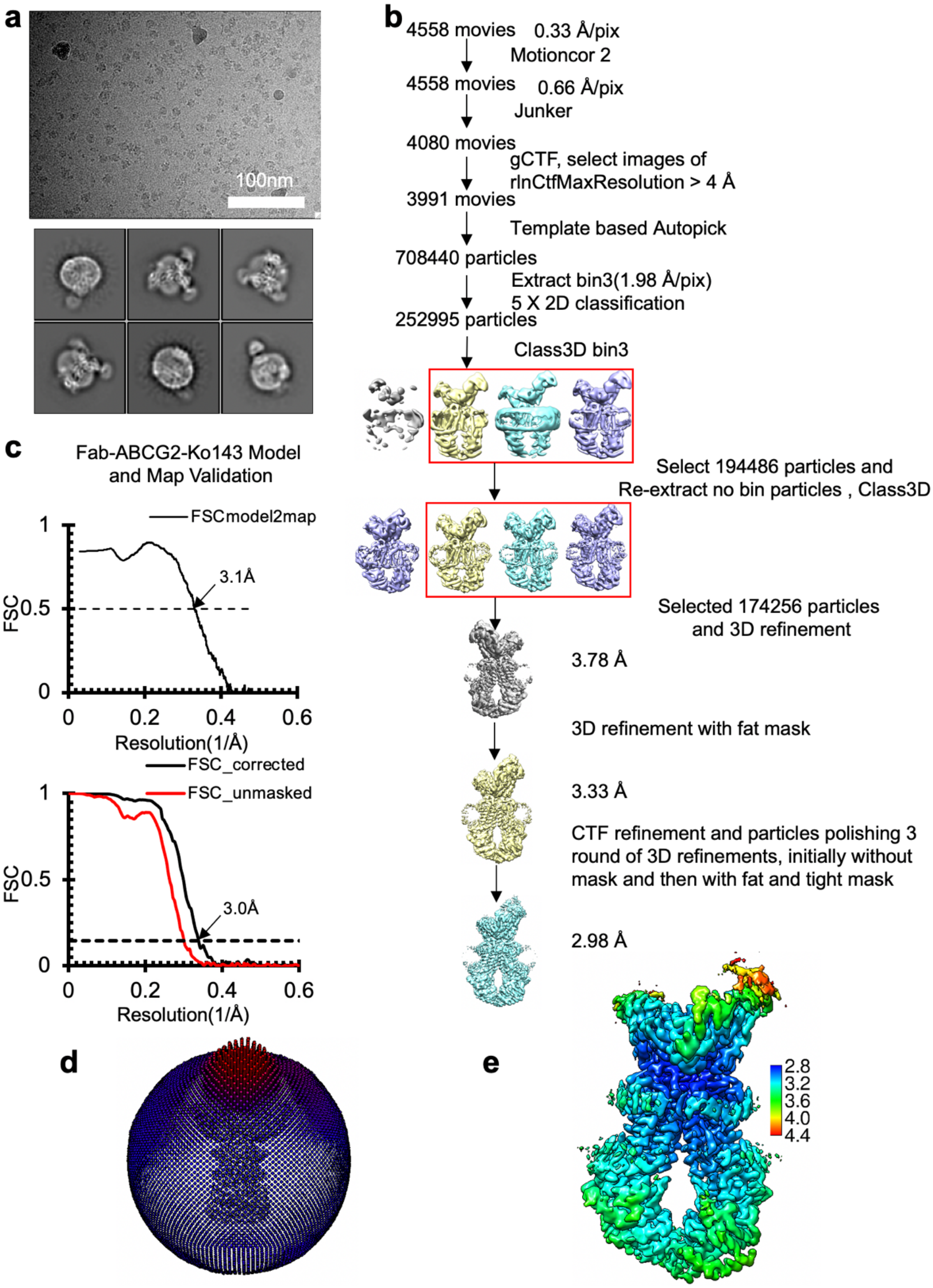

Supplementary Figure 3. Cryo-EM data processing and model validation of ABCG2-Ko143-Fab. **a** Top, a representative motion-corrected 2D micrograph of ABCG2-Ko143-Fab sample among 4558 images collected. White scale bar, 1000 Å. Bottom, Representative 2D classes. **b** Flowchart of data processing of ABCG2-Ko143-Fab sample. Red boxes indicate classes of particles selected for the next round. **c** Map and model validation of ABCG2-Ko143-Fab dataset. **d** Angular distribution plot for ABCG2-Ko143-Fab from RELION. **e** Local resolution of ABCG2-Ko143-Fab structure. FSC corresponds to Fourier shell correlation.

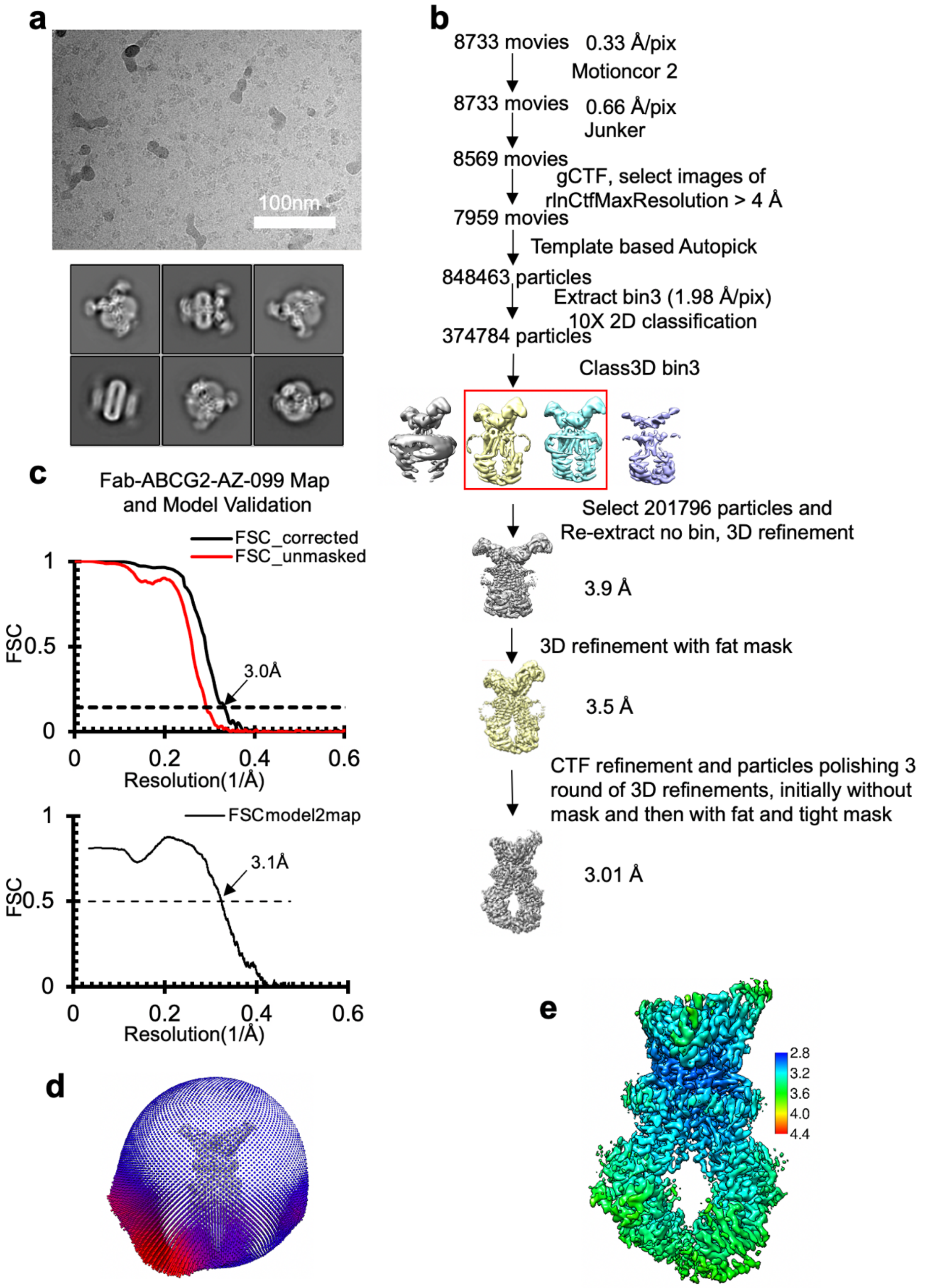

Supplementary Figure 4. Cryo-EM data processing and model validation of ABCG2-AZ99-Fab. **a** Top, a representative motion-corrected 2D micrograph of ABCG2-AZ99-Fab sample among 8733 images collected. White scale bar, 1000 Å. Bottom, Representative 2D classes. **b** Flowchart of data processing of ABCG2-AZ99-Fab sample. Red boxes indicate classes of particles selected for the next round. **c** Map and model validation of ABCG2-AZ99-Fab dataset. **d** Angular distribution plot for ABCG2-AZ99-Fab from RELION. **e** Local resolution of ABCG2-AZ99-Fab structure. FSC corresponds to Fourier shell correlation.

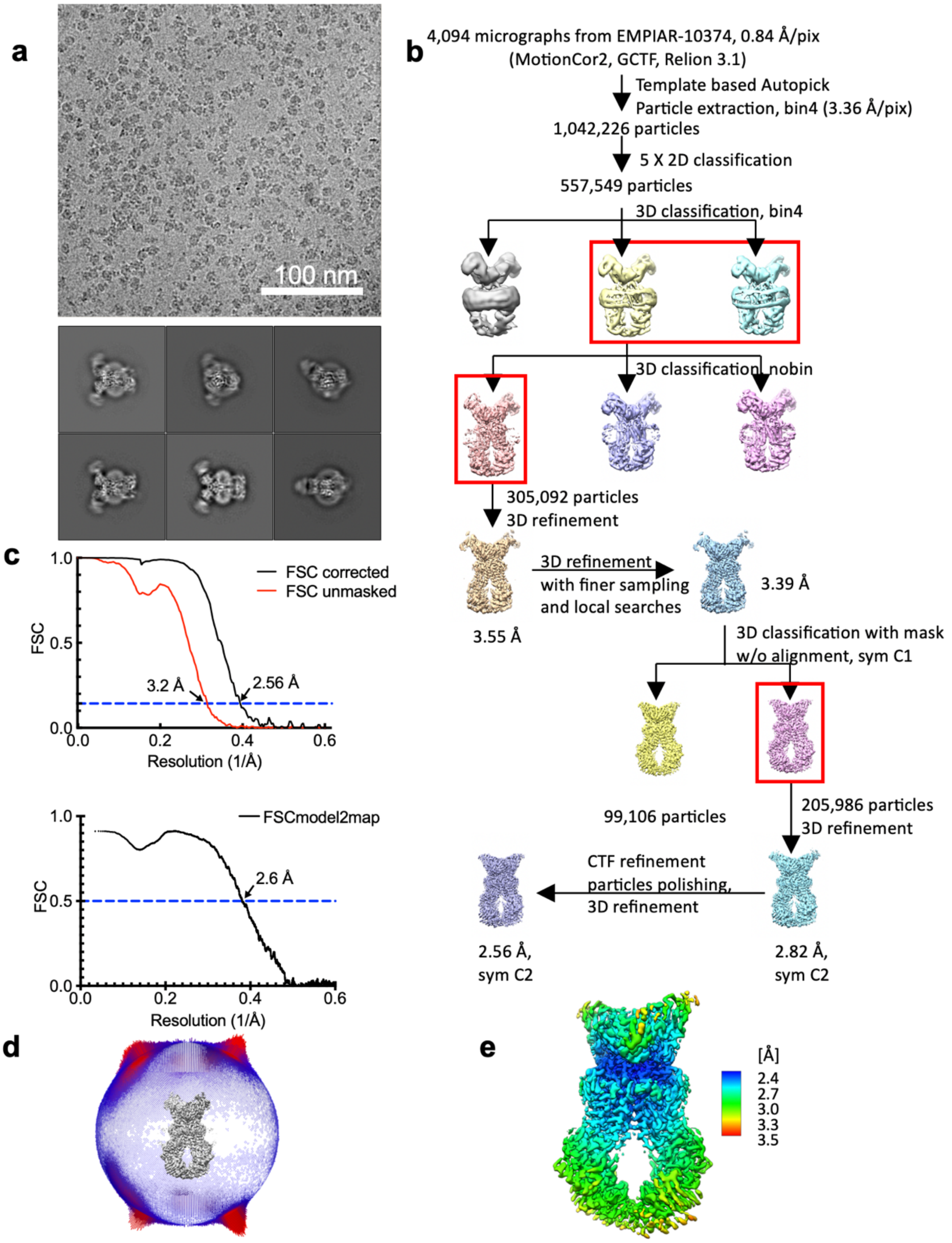

Supplementary Figure 5. Cryo-EM data processing and model validation of ABCG2-MZ29-Fab. **a** Top, a representative motion-corrected 2D micrograph of ABCG2-MZ29-Fab sample among 4,094 images collected. White scale bar, 1000 Å. Bottom, Representative 2D classes. **b** Flowchart of data processing of ABCG2-MZ29-Fab sample. Red boxes indicate classes of particles selected for the next round. **c** Map and model validation of ABCG2-MZ29-Fab dataset. **d** Angular distribution plot for ABCG2-MZ29-Fab from RELION. **e** Local resolution of ABCG2-MZ29-Fab structure. FSC corresponds to Fourier shell correlation.

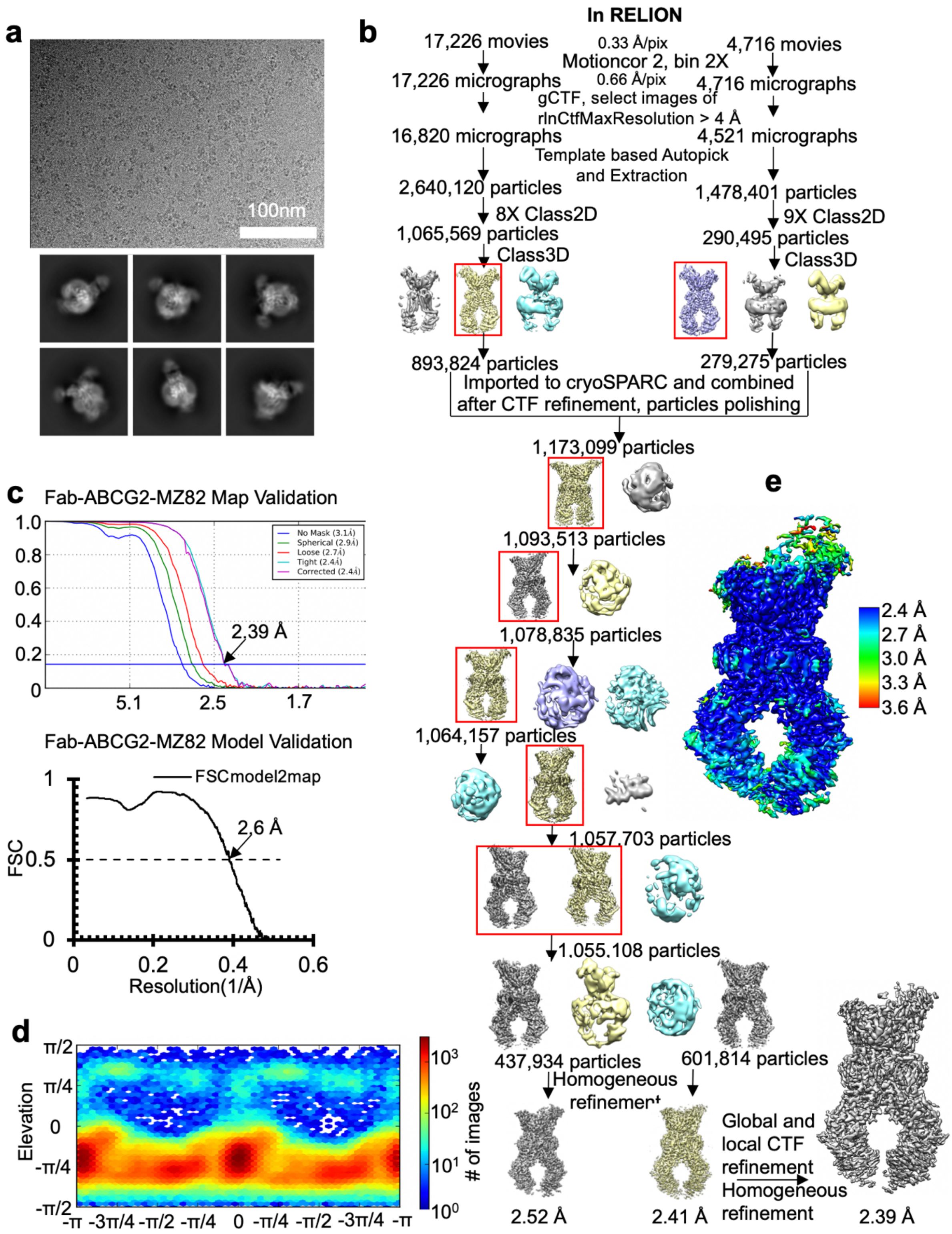

Supplementary Figure 6. Cryo-EM data processing and model validation of ABCG2-MZ82-Fab. **a** Top, a representative motion-corrected 2D micrograph of ABCG2-MZ82-Fab sample among 21,942 images collected. White scale bar, 100 nm. Bottom, Representative 2D classes. **b** Flowchart of data processing of ABCG2-MZ82-Fab sample. Red boxes indicate classes of particles selected for the next round. **c** Map and model validation of ABCG2-MZ82-Fab dataset. **d** Angular distribution plot for ABCG2-MZ82-Fab. e Local resolution of ABCG2-MZ82-Fab structure. FSC corresponds to Fourier shell correlation.

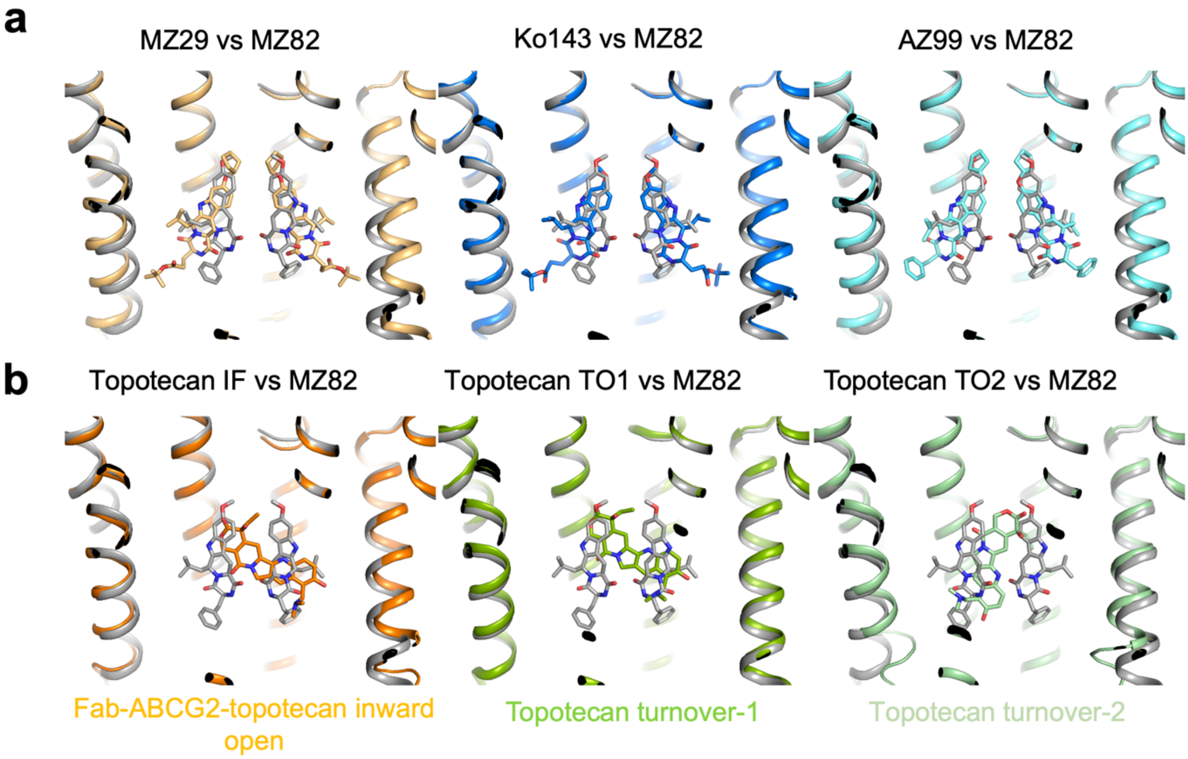

Supplementary Figure 7. The comparison of Fab-ABCG2-MZ82 structure with inhibitor bound structures and with topotecan bound structures. **a** Superposition of TMD of ABCG2-MZ82-Fab structure with TMDs of ABCG2 inhibitor bound structures. **b** Superposition of TMD of ABCG2-MZ82-Fab structure with TMDs of topotecan bound structures. ABCG2-topotecan-Fab inward open is shown in orange (PDB ID: 7NEZ). Topotecan turnover-1 is shown in green (PDB ID: 7OJH). Topotecan turnover-2 is shown in light green (PDB ID: 7OJI). ABCG2 is shown in ribbon and substrates and inhibitors are shown in sticks. The superposition shows the conformation of TMH1 and TMH5' in ABCG2-MZ82-Fab is close to that of topotecan turnover-1 structure.

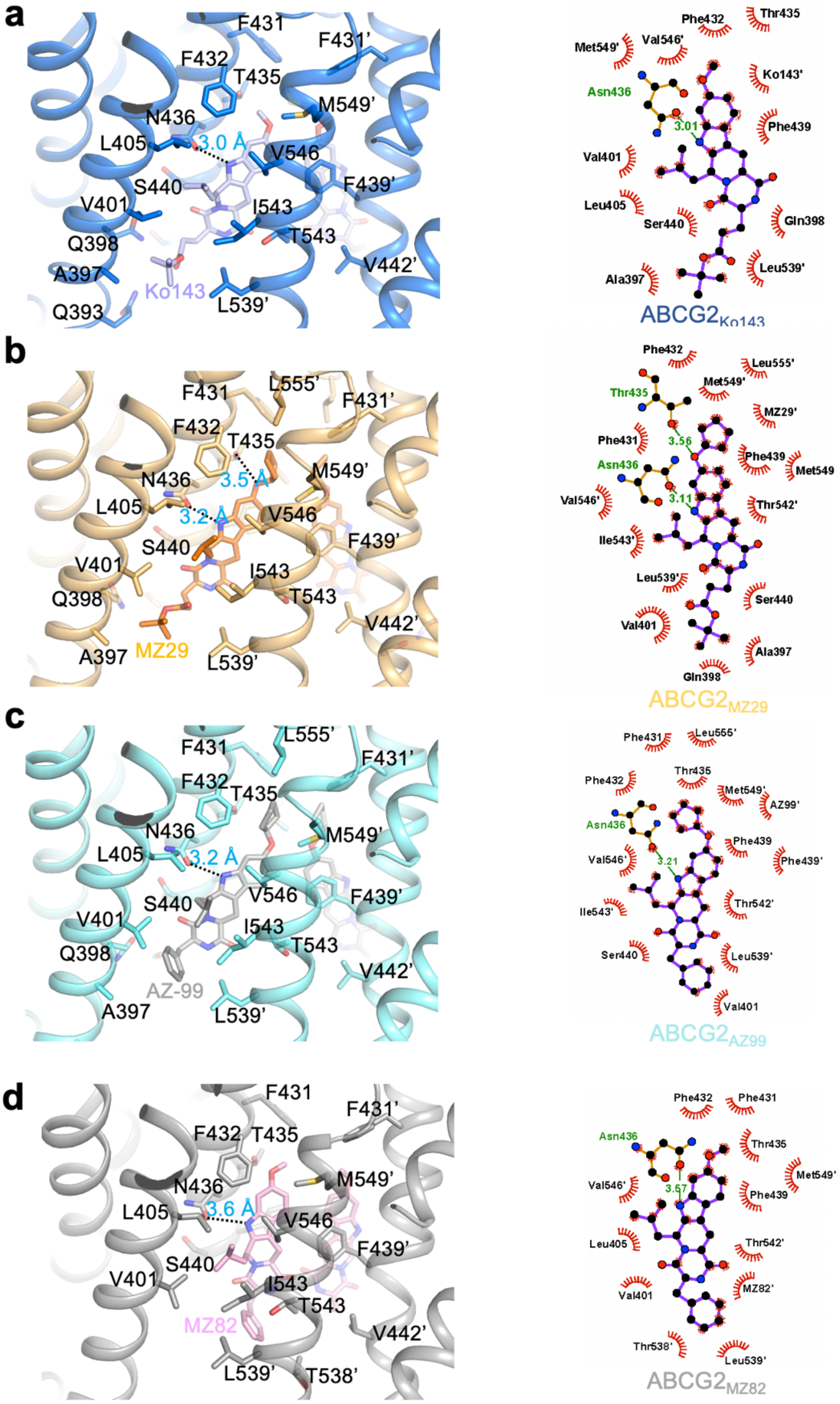

Supplementary Figure 8. Interactions of inhibitors and ABCG2 in Cavity 1. **a** Specific interactions between Ko143 and ABCG2 residues in cavity 1 (left) and "road kill plot" of a Ko143 molecule interacting with the surrounding side chains (right). Spoked arcs represent non-bonding interactions. ABCG2 is shown in ribbons, inhibitors and interacting residues are shown as sticks and labeled, and hydrogen bonds are shown as blue dashed lines with distance indicated. **b c** and **d** Similar to **a** but showing the interaction of MZ29 (**b**), AZ99 (**c**) and MZ82 (**d**), respectively.

Methods

Expression and purification of ABCG2

As we have reported previously, human wild-type ABCG2 (Uniprot: Q9UNQ0) with an N-terminal Flag-tag was expressed in HEK293-EBNA (Thermo Fisher Scientific) cells by transient transfection[1]. Cells were incubated at 37°C for 48-60 hours before harvesting. Harvested Cell were lysed using a Dounce homogenizer and solubilized with 1% DDM, 0.1% CHS (cholesteryl hemisuccinate) (w/v) (Anatrace), 40 mM HEPES buffer pH 7.5, 150 mM NaCl, 10% (v/v) glycerol, 1 mM PMSF (phenylmethylsulfonyl fluoride), 2 µg ml^{-1} DNaseI (Roche), and protease inhibitor cocktail (Sigma). Lysed cells were centrifuged at 100,000*g* and the supernatant was incubated with anti-Flag M2 affinity agarose gel (Sigma). ABCG2 was eluted with Flag peptide (Sigma) and applied to a Superdex 200 increase 10/300 column (GE Healthcare) in 40 mM HEPES, pH 7.5, 150 mM NaCl, 0.026% DDM and 0.0026% CHS (w/v). Peak fractions were collected for further use[2].

5D3Fab-ABCG2-nanodisc preparation

Membrane scaffold protein (MSP) 1D1 was expressed in *E. coli* and purified as described[3]. ABCG2 reconstitution in nanodiscs was performed as following methods[1]. In brief, BPL (brain polar lipid, Avanti Polar Lipids) and CHS (cholesteryl hemisuccinate) were mixed at a 4:1 (w/w) ratio. Lipids were solubilized with a 3x molar excess of sodium cholate using a sonic bath.
Detergent-purified ABCG2 was first incubated with a three-fold molar excess of Fab and then 30 µM inhibitor (MZ82, AZ99 or Ko143). Lipids were then mixed with detergent-purified ABCG2-Fab and MSP1D1 at a molar ratio of 100:5:0.2 (lipid:MSP: ABCG2). The detergent was removed by the addition of Bio-Beads SM-2 (Biorad) and incubated at 4 °C overnight. After the removal of Biobeads, the sample was centrifuged at 100,000*g* for 30min. The supernatant was loaded on a Superdex 200 increase column equilibrated with buffer containing 40mM HEPES PH7.5 and 150mM NaCl. Fractions containing nanodisc-reconstituted ABCG2 were collected.

ABCG2-liposome preparation

ABCG2-containing proteoliposomes were prepared as described[1, 4]. In brief, brain polar lipid (BPL) was mixed with cholesterol (Chol) at a 4:1 (w/w) ratio. Liposomes were resuspended in transport buffer (25 mM HEPES pH 7.5, 150 mM NaCl) and extruded using a 400nm polycarbonate filter (Avanti Polar Lipids). To reconstitute ABCG2, liposomes were destabilized with 0.17% (v/v) Triton X-100. Detergent-purified ABCG2 was mixed with liposomes at a 100:1 (w/w) lipid: protein ratio. The detergent was then removed using Bio-Beads added in multiple batches. Proteoliposomes were collected using centrifugation at 100,000*g*. The proteoliposome pellet was resuspended in transport buffer at a final lipid concentration of 10 mg ml^{-1}.

ATPase and transport assays

ATPase assays with nanodiscs or proteoliposomes were performed at 37°C in the presence of 2 mM ATP and 10 mM $MgCl_2$. For ATPase assay with proteoliposomes, E_1S was added at 50 µM. The concentration of inorganic phosphate released by the hydrolysis of ATP was measured by tracking absorbance at 850 nm following the classical molybdate method[5]. ATPase rates were determined using linear regression in GraphPad Prism v8 and v9.
For transport assays, proteoliposomes in transport buffer (25 mM HEPES pH 7.5, 150 mM NaCl) were extruded through a 400 nm polycarbonate filter. $MgCl_2$ (5 mM) and E_1S (50 µM, containing mixtures of ^3H-E_1S and ^1H-E_1S) were added in the presence or absence of inhibitors and the samples were incubated for 5 min at 30°C. Transport reactions were initiated by adding ATP (2 mM) and stopped by adding an aliquot to ice-cold transport buffer containing unlabelled E_1S (100 µM). The samples were filtered with a Multiscreen vacuum manifold (MSFBN6B filter plate, Millipore) and washed three times. Radioactivity trapped on the filters

was measured with the microplate scintillation counter (Perkin Elmer 2450 Microbeta2). The initial E_1S transport rates were determined using linear regression in GraphPad Prism.

Cryo-EM sample preparation

All grids were prepared using a Vitrobot Mark IV (Thermo Fisher Scientific), with the environmental chamber set at 100% humidity and 4°C. Nanodisc-reconstituted 5D3-Fab-ABCG2 (0.5 mg ml^{-1}) was incubated with 30 µM MZ82, AZ99 or Ko143, respectively. 3.5 µl sample was applied on glow-discharged Quantifoil carbon grids (300 mesh, R 1.2/1.3 copper) 10 min after incubation. Grids were blotted for 2.5 s with blot force 1 and flash-frozen in a mixture of liquid ethane and propane.

Cryo-EM data acquisition

CryoEM data was collected with a 300 keV Titan Krios (Thermo Fisher Scientific) transmission electron microscope (TEM) equipped with a Gatan BioQuantum 1967 filter and a Gatan K3 camera. Images were recorded with 3 exposures per hole using *EPU* 2 in super-resolution mode with a 20 eV slit width of the energy filter and at a nominal magnification of 130,000 x, resulting in a calibrated super-resolution pixel size of 0.33 Å. Defocus was set to vary from −0.6 to −2 µm. Each image was dose fractionated to 40 frames.

The dose was 1.45 e^-/Å²/frame (total dose 58 e^-/Å²) with 1.01s total exposure time for 5D3-Fab-ABCG2-ko143 and Fab-ABCG2-AZ99 sample. 4,558 movies were collected for 5D3-Fab-ABCG2-Ko143 and 8,733 movies were collected for 5D3-Fab-ABCG2-AZ99.

For the 5D3-Fab-ABCG2-MZ82 sample, 17,226 movies with dose 1 e^-/Å²/frame (Total dose 42 e^-/Å and 1.25 s total exposure time) and 4,716 movies with 1 e^-/Å²/frame and CDS (correlated-double sampling mode) mode (Total dose 42 e^-/Å and 2.5 s total exposure time) were collected and combined.

The super-resolution micrographs were binned twice and motion-corrected by Fourier cropping and drift-corrected and dose-weighted using *MotionCor2*[6]. Micrographs were visually inspected, and bad micrographs were removed manually if processed in *RELION3.1*. Cryo-EM data collection statistics in this study are presented in Supplementary table 1.

Image processing

The data processing details are presented in Supplementary Figure 2-4. The Contrast transfer function (CTF) parameters were estimated with *Gctf*[7] integrated with *RELION*3.1. Micrographs with an estimated resolution lower than 4 Å were excluded from further processing.

For the 5D3-Fab-ABCG2-Ko143 dataset, 3,991 micrographs with estimated MaxResolution ≤4 Å were selected for particle picking. 708,440 particles were picked with template-based autopick and extracted with three-fold bin which result in a pixel size of 1.98 Å/pix. After 5 rounds of classifications, 252,995 particles belonging to 'good' 2D classes were selected. The map of 5D3-Fab-ABCG2-MZ29 (EMD-3953) was rescaled and used as an initial model for the first round of 3D classification with 4 classes. Three good 3D classes with194,486 particles were selected and re-extracted with no bin and a pixel size of 0.66 Å/pix. The second round of 3D classification was performed with 4 classes for unbinned particles. Three 3D classes (174,256 particles) with well-defined features were selected and used for further 3D refinement. The selected particles were refined to 3.78 Å. Then a soft mask was generated based on the refined map and used for further 3D refinement, which led to a 3.33 Å map. The good particles were further Bayesian polished, and CTF refined. The polished particles underwent 3 rounds of 3D refinement. The first round was without a mask, the second round with a very soft 'fat' mask and the third round with a soft mask around the entire complex, masking out the nanodisc and Fc (constant domain of the Fabs). The final 3D refinement led to a 3 Å resolution map after postprocessing with an automatically determined B factor of -50 Å². The 5D3-Fab-ABCG2-AZ99 dataset and the published 5D3-Fab-ABCG2-MZ29 were processed similarly[8].

For the 5D3-Fab-ABCG2-MZ82 dataset, 16,838 micrographs collected with non-CDS mode and 4,558 micrographs with CDS mode were selected after CTF estimation. 2,640,120

particles from the non-CDS mode subset and 1,478,401 particles from the CDS mode subset were extracted and subjected to 8 or 9 rounds of 2D classification, respectively. 1,065,569 particles from the 2D classification of the non-CDS subset and 290,495 particles from the 2D classification of the CDS subset were selected and used for further 3D classification separately. 893,824 good particles from the non-CDS subset and 279,275 particles from the CDS subset were selected from 3D classification respectively. CTF refinement and Bayesian particle polishing were performed for each subset. Polished particles were further exported into *CryoSPARC* v2 and combined. 7 rounds of 3D classifications were performed, in each round, the 3D class with the highest resolution was selected. After the final 3D classification, 601,814 particles were selected for homogeneous refinement, which resulted in a 2.41 Å map in C1 symmetry. The particles further underwent Global CTF refinement, Local CTF refinement and Homogeneous refinement and the final and best map was 2.39 Å resolution in C1 symmetry.

Both C1 and C2 symmetry were applied for 3D refinement of the maps and in both cases, two copies of ligands were found in Cavity 1 of ABCG2.

Model building and refinement

Coot 0.9 was used for all model-building steps[9]. 5D3-Fab-ABCG2-MZ29 model was docked into 5D3-Fab-ABCG2-AZ99 or 5D3-Fab-ABCG2-ko143 maps and used as the reference for manual rebuilding[8]. Topotecan turnover-1 model was docked into the 5D3-Fab-ABCG2-MZ82 map and used for manual building. The coordinates and restraint files of AZ-99, MZ82 and Ko143 were generated in *eLBOW* of *Phenix* [10]. All structures were refined against their final maps respectively in real space refinement of Phenix[11]. In the final refinement, reciprocal-space refinement of the B factors and minimization global refinements were applied together with standard geometry, rotamer, Ramachandran plot, Cβ, non-crystallographic symmetry (NCS) and secondary structure restraints. The quality of the final model was assessed by *MolProbity*[12]. The refinement statistics are in Supplementary table 1.

For model validation, we applied 0.3 Å random shifts to the final models using phenix_pdb_tools[13]. The scrambled model was refined against one of the unfiltered half maps (half map A). The Fourier shell correlation between the refined scrambled model and half map A was plotted as FSC_{work}. The Fourier shell correlation between the refined scrambled model and half map B was plotted as FSC_{free}. The overlay between the FSC_{work} and FSC_{free} indicated no over-fitting presence.

System Preparation for Molecular Dynamics Simulations

MZ29-, MZ82-, AZ99-, and Ko143-bound ABCG2 structures were prepared for molecular dynamics (MD) simulations, following the protocol described below. The ABCG2 cryo-EM structures were captured in complexes with two inhibitory antibody fragments. Prior to the MD simulations, these fragments were removed. Missing side-chains and hydrogen atoms were added to the structures, with *PSFGEN* (https://www.ks.uiuc.edu/Research/vmd/plugins/psfgen/) in VMD[14].

The experimental models contain two identical monomers and miss three disordered regions: residues 47-60, 301-328, and 354-368. The first and third missing regions (47-60 and 354-368) were modeled into the structure employing Modeller[15]. The second region was not modeled, dividing each monomer into two polypeptide segments. Neutral N- and C-terminal caps were added to the beginning and end of each segment, with *PSFGEN*. The coordinates of each ligand at the binding pocket were obtained from the cryo-EM models. The protonation state and pKa values of all titratable residues were estimated using PROPKA[16, 17]. The protein structures were internally hydrated using *DOWSER*[18, 19]. The lipid bilayer used to embed the protein was constructed in *CHARMM-GUI*[20]. The orientation of the protein in the bilayer was obtained from orientation of proteins in membranes (OPM)[21]. The ligand-bound proteins each was then inserted into a lipid bilayer composed of palmitoyl-oleoyl-phosphatidyl-choline (POPC), palmitoyl-oleoyl-phosphatidyl-ethanolamine (POPE), palmitoyl-oleoyl-phosphatidyl-inositol (POPI), palmitoyl-oleoyl-phosphatidyl-serine (POPS) palmitoyl-sphingomyelin (PSM), palmitoyl-oleoyl-phosphatidic-acid (POPA), and cholesterol (CHOL) at a

POPC:POPE:POPI:POPS:PSM:POPA:CHOL molar ratio of 39:6:0:0:21:0:34 and 17:25:8:11:9:1:29 for outer and inner leaflets, respectively, followed by removing steric clashes between lipids and the protein. The protein-membrane complex was then solvated with water including 150 mM NaCl in VMD (system size: ~359,000 atoms).

Simulation Conditions
All MD simulations were performed employing the *NAMD* simulation package[22, 23] and the fully atomistic CHARMM36m[24] and CHARMM36[25] force fields for the protein and lipids, respectively. The TIP3P model was used for water molecules[26]. All the ligands were parameterized using the CHARMM general force field (CGenFF) webserver (https://cgenff.paramchem.org) [27, 28]. A 12-Å cutoff was used for short-range, non-bonded interactions, with switching starting at 10 Å. Long-range electrostatic interactions were calculated employing the particle mesh Ewald (PME) algorithm[29] with a grid density of 1 $Å^{-1}$, and a PME interpolation order of 6. All bonds involving hydrogen atoms were constrained using the SHAKE algorithm[30]. Temperature was maintained at 310 K using Langevin thermostat with a damping coefficient of 1 ps^{-1}. Pressure was kept at 1 atm using the Nosé-Hoover Langevin piston barostat with period and decay of 100 and 50 fs, respectively[31, 32]. All systems were simulated in a flexible cell allowing the dimensions of the periodic cell to change independently while maintaining the cell aspect ratio in the *x-y* plane (membrane plane). The simulation timestep was set to 2 fs. Lennard-Jones and PME forces were updated every and every other timestep, respectively. Atomic coordinates were saved every 10 ps.
Before calculating the binding free energy, each system was equilibrated using the procedure described below: (1) 10,000 steps of minimization, followed by 5 ns of equilibration, in which positional harmonic restraints (k = 10 $kcal.mol^{-1}.Å^{-2}$) were applied to the protein's heavy atoms in the cryo-EM model, as well as to the heavy atoms of the ligand in the binding pocket. Furthermore, the *z* position (normal to the membrane plane) of the phosphorus atoms of phospholipids and the oxygen atoms of cholesterol molecules were also restrained with a harmonic potential (k = 5 $kcal.mol^{-1}.Å^{-2}$); (2) 40 ns of equilibration with only the protein backbone and heavy atoms of the ligand restrained harmonically (k = 10 $kcal.mol^{-1}.Å^{-2}$).

Free energy calculation of ligand binding affinity
Free energy perturbation (FEP) simulations[33, 34] were performed on the MZ29-, MZ82-, AZ99-, and Ko143-bound structures to compare the relative binding affinities of the four ligands. During the FEP protocol, two copies of ligands were created at the binding pocket while annihilated from two different locations in bulk solution far from the binding pocket and from each other. The FEP calculations were initiated from the last frame of Step 2 of the equilibration protocol (see above). During the FEP calculations, the protein backbone heavy atoms were restrained (k = 10 $kcal.mol^{-1}.Å^{-2}$) to the experimental model to avoid undesired structural deviations. All other simulation parameters were the same as in the equilibrium simulations described above.
The alchemical transformations were performed in both forward (λ increasing from 0 to 1; ligand creation in the binding pocket and annihilation from solution) and backward (λ decreasing from 1 to 0; ligand annihilation from the binding pocket and creation in solution) directions to assess the reversibility and convergence. A soft-core van der Waals radius-shifting coefficient of 5 Å was used. In the forward transformation, the van der Waals and electrostatic interactions of the annihilated particles were linearly decoupled to the simulation over a λ range of 0-1 and 0-0.5, respectively, whereas in the backward transformation these interactions were linearly coupled to the simulation in the corresponding λ range. Each FEP calculation along the λ reaction path was divided into 50 equally-spaced consecutive windows in the λ space to ensure gradual transformation. Within each window, 5000 steps of minimization and 100 ps of MD relaxation were followed by 1 ns of data collection at a frequency of once every 20 fs, resulting in an accumulated simulation time of 110 ns for each system. To prevent the dissociation of the ligand during the FEP calculation and alchemical transformation, harmonic positional restraints were applied to the created and annihilated ligands (k = 10 $kcal.mol^{-1}.Å^{-2}$). All the simulations were performed employing the FEP module

of NAMD[35]. The outputs were analyzed using the ParseFEP[36] plugin in VMD, with the statistical error estimated using the implemented Bennett Acceptance Ratio (BAR) method[37].

Figure Preparation
Molecular images were prepared with PyMOL (The PyMOL Molecular Graphics System, Version 2.4.0 Schrödinger, LLC) and VMD[14]. Data plots were prepared with GraphPad Prism 9 and UCSF ChimeraX[38]. Movies were prepared with UCSF Chimera[39].

Synthetic protocols, analytical data, and copies of ^{1}H- and ^{13}C-NMR spectra for final products and intermediates

General. All solvents used for reactions were purchased as anhydrous grade from Acros (puriss. dried over molecular sieves; H_2O <0.005%) and used without purification. Solvents for extractions, flash column chromatography (FC) and thin layer chromatography (TLC) were purchased as commercial grade and distilled prior to use. All non-aqueous reactions were performed under an argon atmosphere using flame-dried glassware and standard syringe/septa techniques. Commercially available reagents were used without further purification. In general, reactions were magnetically stirred and monitored by TLC performed on Merck TLC aluminum sheets (silica gel 60 F254). Spots were visualized with UV light (λ = 254 nm) or through staining with $Ce_2(SO_4)_3$/phosphomolybdic acid/H_2SO_4 (CPS) or $KMnO_4$/K_2CO_3. Purification of products by FC was performed using Fluka silica gel 60 (particle size 40–63 µm). **Melting points** were obtained in open capillary tubes using a Büchi melting point apparatus B-540 and are uncorrected. **^{1}H- and ^{13}C-NMR spectra** were recorded in $CDCl_3$, DMSO-*d*$_6$ or CD_3OD on a Bruker AV-400 400 MHz or on a Bruker AV-500 500 MHz spectrometer at room temperature. Chemical shifts (δ) are reported in ppm and are referenced to chloroform (δ 7.26 ppm for ^{1}H, δ 77.16 ppm for ^{13}C), methanol (δ 3.31 ppm for ^{1}H, δ 49.00 ppm for ^{13}C) or dimethylsulfoxide (δ 2.50 ppm for ^{1}H, δ 39.52 ppm for ^{13}C), respectively. All ^{13}C-NMR spectra were measured with complete proton decoupling. Data for NMR spectra are reported as follows: s = singlet, d = doublet, t = triplet, m = multiplet, br = broad signal, J = coupling constant in Hz. **Infrared spectra (IR)** were recorded on a Jasco FT/IR-6200 instrument. Resonance frequencies are given as wavenumbers in cm^{-1}. **Optical rotations** were measured on a Jasco P-1020 polarimeter and are reported as follows: $[\alpha]_D^{20}$, concentration (g/100 ml) and solvent. **High resolution mass spectra** (HRMS) were recorded by the ETH Zürich MS service; HRMS (ESI) spectra were obtained on a Varian IonSpec spectrometer.

The synthesis of MZ82 (**6**) has been described previously:
M Zechner, C A. Castro Jaramillo, N. S. Zubler, M F. Taddio, L. Mu, K.-H Altmann, S. D. Krämer. In Vitro and In Vivo Evaluation of ABCG2 (BCRP) Inhibitors Derived from Ko143. *J. Med. Chem.* **2023,** *66*, 6782-6797. DOI: 10.1021/acs.jmedchem.3c00168

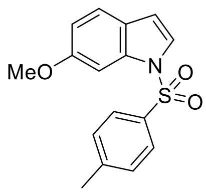

13

6-Methoxy-1-tosyl-1*H*-indole (13): NaH (60% in oil, 163 mg, 4.08 mmol, 1.20 eq.) was added at 0 °C to a solution of 6-methoxyindole (**12**) (500 mg, 3.40 mmol, 1.00 eq.) in 25 mL of DMF. The resulting mixture was stirred at 0 °C for 30 min (during which period it turned green) before adding *p*-TsCl (970 mg, 5.10 mmol, 1.50 eq.). The reaction mixture was allowed to warm to rt overnight and sat. aq. NH_4Cl was added until the pH was neutral. The precipitate formed was collected by filtration, washed with H_2O and dried *in vacuo* to give indole **14** as a beige solid (820 mg, 80%).

R_f = 0.62 (hexane/AcOEt 2:1); **mp** = 122-125 °C; **^{1}H NMR** (400 MHz, $CDCl_3$) δ (ppm) 7.77 – 7.71 (m, 2H), 7.53 (d, *J* = 2.3 Hz, 1H), 7.44 (d, *J* = 3.6 Hz, 1H), 7.38 (d, *J* = 8.6 Hz, 1H), 7.24 – 7.20 (m, 2H), 6.85 (dd, *J* = 8.6, 2.4 Hz, 1H), 6.57 (dd, *J* = 3.7, 0.8 Hz, 1H), 3.87 (s, 3H), 2.34 (s, 3H); **^{13}C NMR** (101 MHz, $CDCl_3$) δ (ppm) 158.0, 145.0, 136.1, 135.5, 130.0, 126.9, 125.3, 124.6, 121.9, 112.7, 109.1, 98.0, 55.9, 21.7; **v_{max} (neat)/cm^{-1}** = 2924, 2853, 1615, 1597, 1582, 1527, 1488, 1464, 1431, 1368, 1363, 1316, 1288, 1270, 1214, 1187, 1170, 1117, 1092, 1077, 1031, 999, 928, 811, 751, 716, 703, 672, 627, 581, 540; **HRMS** (ESI): m/z calcd for $C_{16}H_{16}N_1O_3S_1$ $[M+H]^+$: 302.0845, found: 302.0847.

^{1}H NMR ($CDCl_3$, 400 MHz)

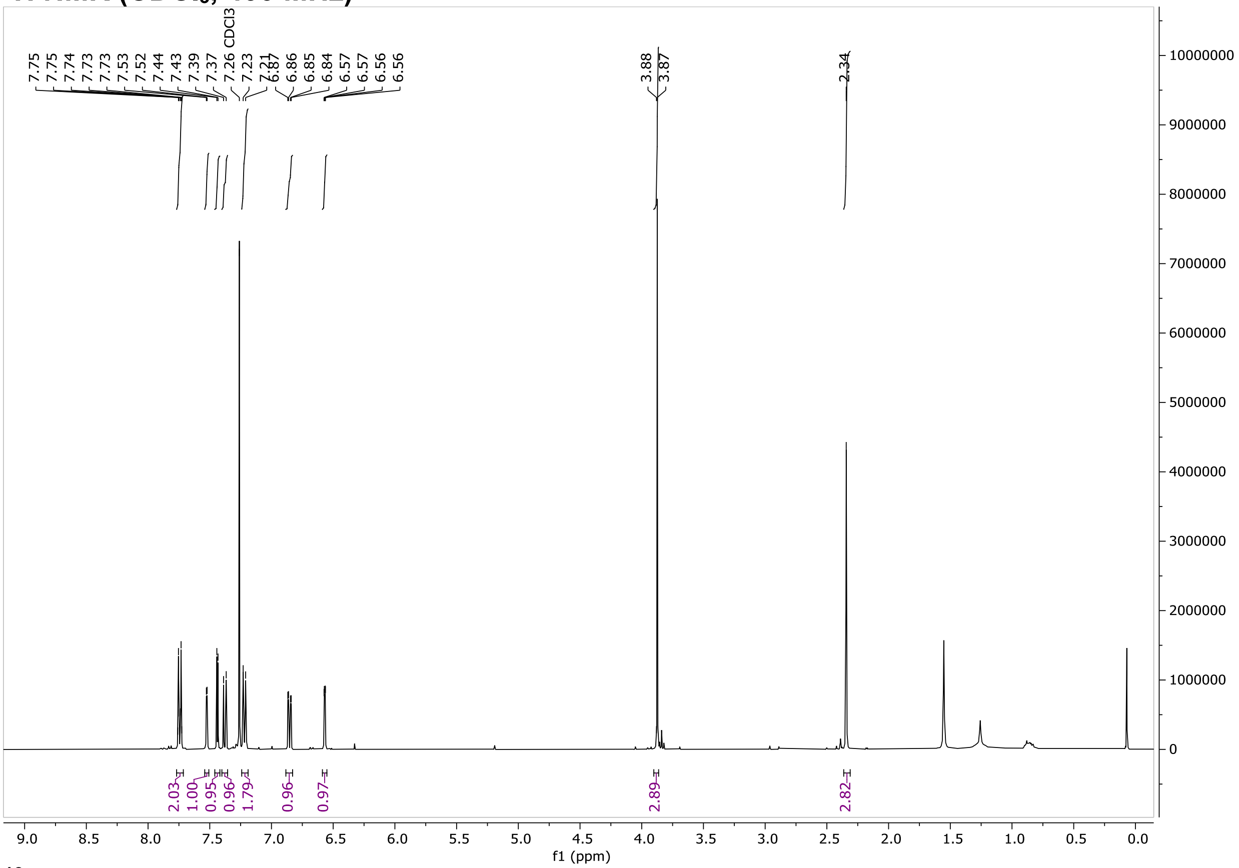

^{13}C NMR ($CDCl_3$, 101 MHz)

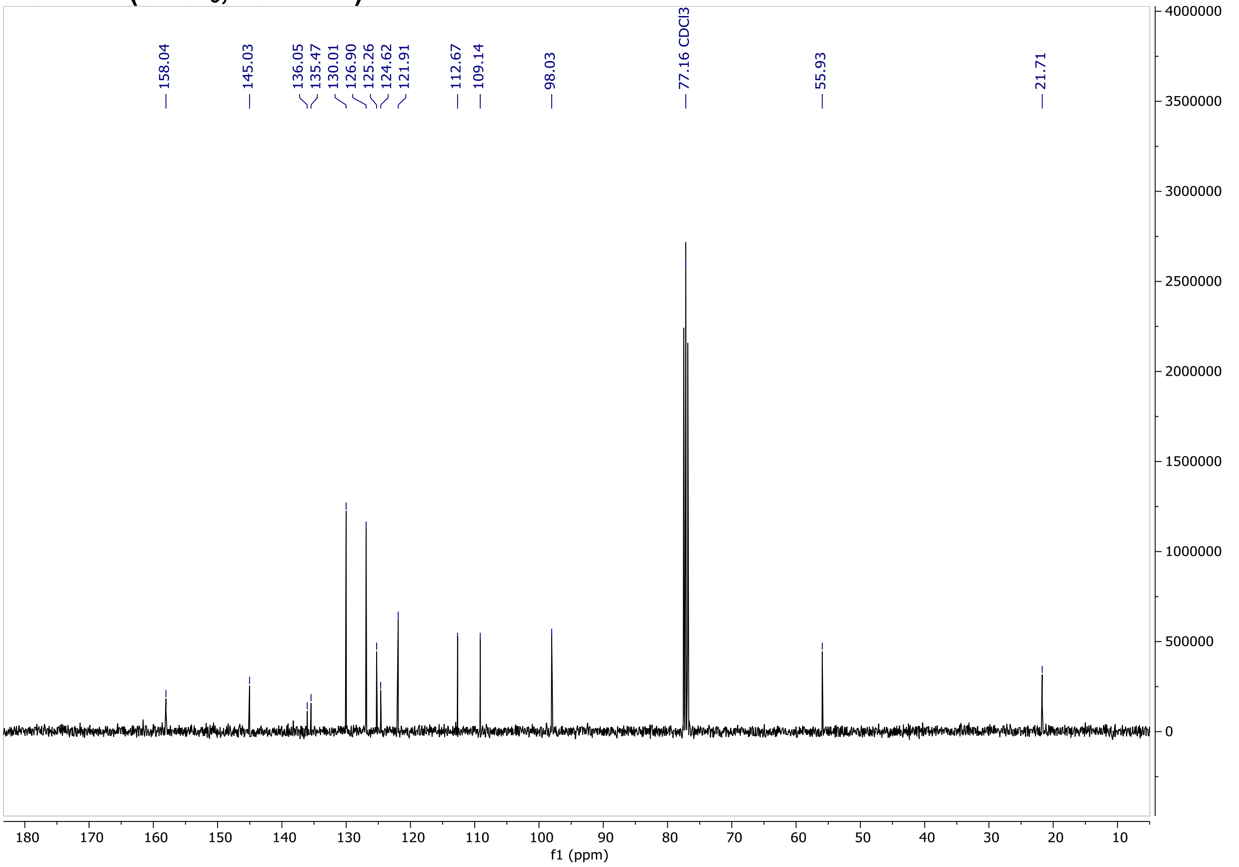

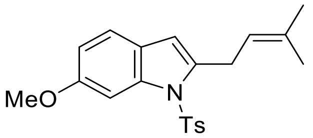

14

6-Methoxy-2-(3-methylbut-2-en-1-yl)-1-tosyl-1*H*-indole (14): To a solution of **13** (4.00 g, 13.27 mmol, 1.00 eq.) in THF (142 mL) was added dropwise a solution of *n*BuLi in hexanes (9.1 mL, 14.60 mmol, 1.10 eq.) at -78 °C. The reaction mixture was stirred at this temperature for 45 min, prenyl bromide (4.6 mL, 39.82 mmol, 3.00 eq.) was added and the mixture was stirred at -78 °C for another 15 min. It was then allowed to warm to rt slowly and stirred for 16 h. Sat. aq. NH_4Cl (4 mL) was added and the mixture was extracted with CH_2Cl_2 (3 x 5 mL). The combined organic layers were washed with brine (2 x 5 mL), dried over $MgSO_4$, filtered and the volatiles were removed *in vacuo*. The residue (a liquid brown oil) was purified by FC (hexane/AcOEt 15:1) to give **14** as a yellow oil (3.80 g, 78%).

R_f = 0.8 (hexane/AcOEt 2:1); **^{1}H NMR** (400 MHz, $CDCl_3$) δ (ppm) 7.76 (d, *J* = 2.3 Hz, 1H), 7.65 – 7.61 (m, 2H), 7.26 (d, *J* = 8.5 Hz, 1H), 7.19 (d, *J* = 8.2 Hz, 2H), 6.83 (dd, *J* = 8.5, 2.3 Hz, 1H), 6.26 (q, *J* = 1.2 Hz, 1H), 5.36 (dddd, *J* = 8.6, 5.8, 2.9, 1.4 Hz, 1H), 3.88 (s, 3H), 3.62 (d, *J* = 7.2 Hz, 2H), 2.35 (s, 3H), 1.77 (d, *J* = 1.4 Hz, 3H), 1.61 (d, *J* = 1.2 Hz, 3H); **^{13}C NMR** (101 MHz, $CDCl_3$) δ (ppm) 157.4, 144.8, 140.1, 138.5, 136.5, 134.8, 129.9, 126.4, 123.7, 120.6, 120.0, 112.4, 108.8, 99.7, 56.0, 28.1, 25.9, 21.7, 17.9; **v_{max} (neat)/cm^{-1}** = 2956, 1726, 1498, 1455, 1441, 1378, 1321, 1290, 1222, 1174, 1140, 1084, 1023, 976, 906, 846, 806, 773, 751, 698, 613, 569, 542, 461; **HRMS** (ESI): m/z calcd for $C_{21}H_{24}N_1O_3S_1$ $[M+H]^+$: 370.1471, found: 370.1474.

^{1}H NMR (CDCl$_3$, 400 MHz)

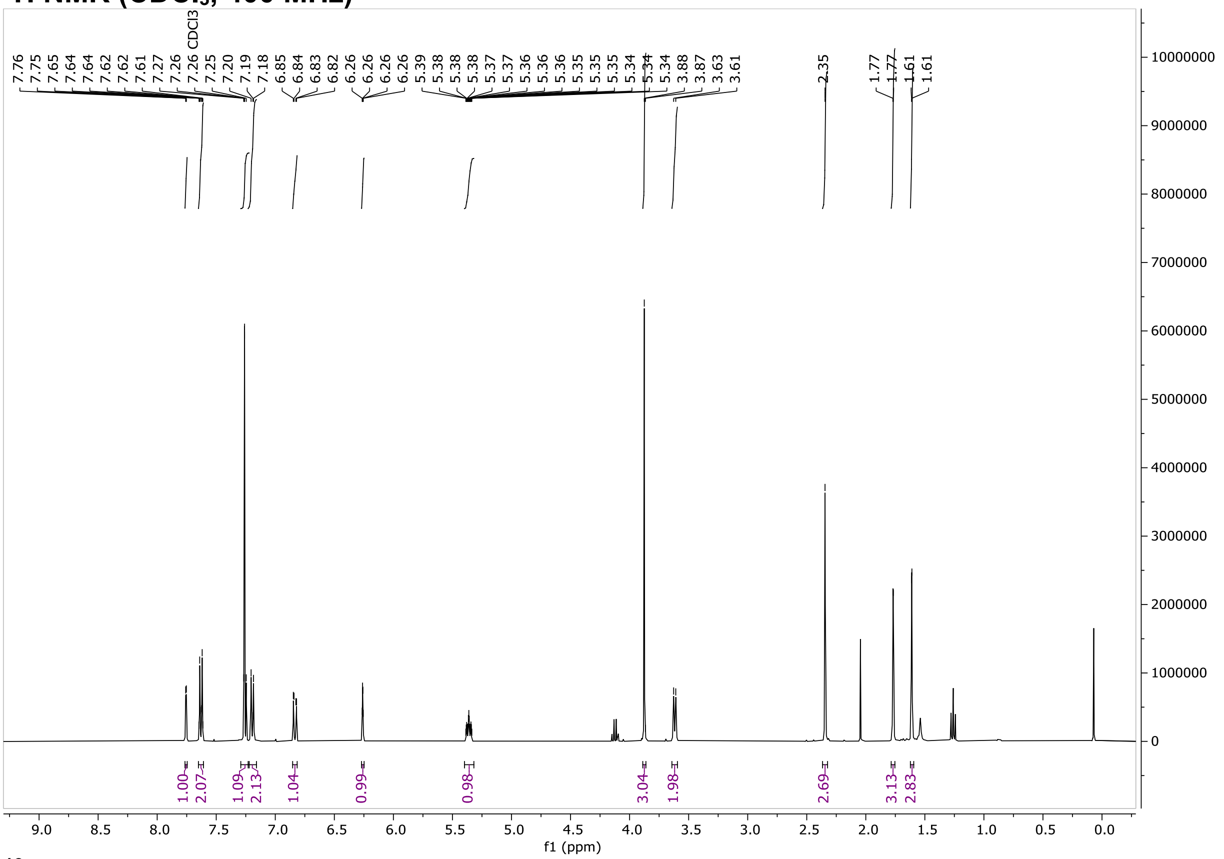

^{13}C NMR (CDCl$_3$, 101 MHz)

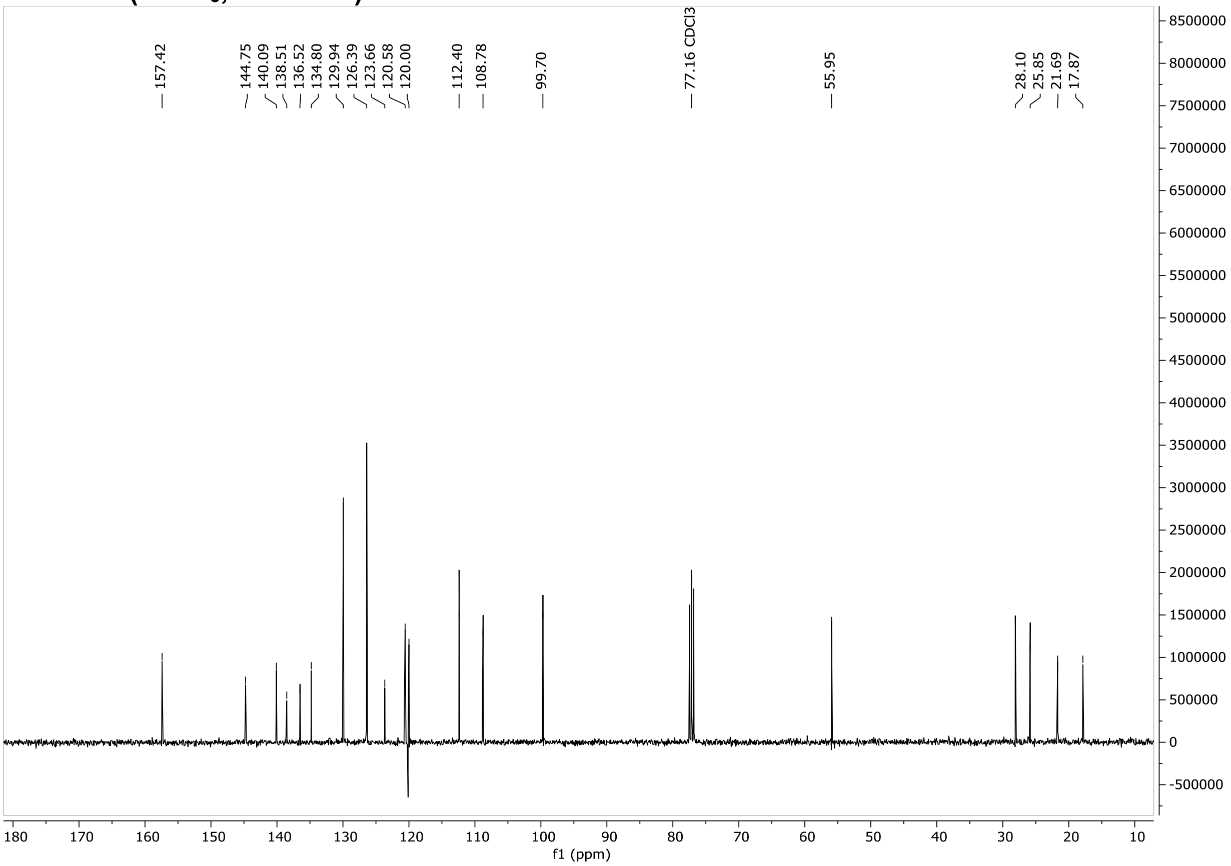

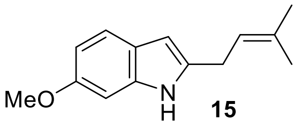
15

6-Methoxy-2-(3-methylbut-2-en-1-yl)-1H-indole (15):[40] Mg (178 mg, 7.30 mmol, 18.0 eq.) was added to a solution of **14** (150 mg, 0.40 mmol, 1.00 eq.) in MeOH (7.5 mL). The reaction mixture was sonicated for 1.5 h and diluted with CH_2Cl_2. The organic layer was washed with HCl (1N), $NaHCO_3$ (aq., sat.), H_2O, dried with $MgSO_4$, filtered and the solvent was evaporated *in vacuo*. The residue was purified by FC (hexane/AcOEt 10:1) to obtain **15** as a pale brownish solid (84.5 mg, 97%).

R_f = 0.74 (hexane/AcOEt 2:1); **mp** = 92 – 93.5 °C (*lit.* 87.0 – 90.2 °C);1 **^{1}H NMR** (400 MHz, $CDCl_3$) δ (ppm) 7.75 (s, 1H), 7.38 (d, *J* = 8.5 Hz, 1H), 6.82 (d, *J* = 2.3 Hz, 1H), 6.74 (dd, *J* = 8.6, 2.3 Hz, 1H), 6.15 (s, 1H), 5.38 (tq, *J* = 7.4, 1.5 Hz, 1H)), 3.83 (s, 3H), 3.46 (d, *J* = 7.3 Hz, 2H), 1.79 (d, *J* = 1.2 Hz, 3H), 1.74 (d, *J* = 1.2 Hz, 3H); **HRMS** (ESI): m/z calcd for $C_{14}H_{18}N_1O_1$ $[M+H]^+$: 216.1383, found: 216.1377.

^1H NMR ($CDCl_3$, 400 MHz)

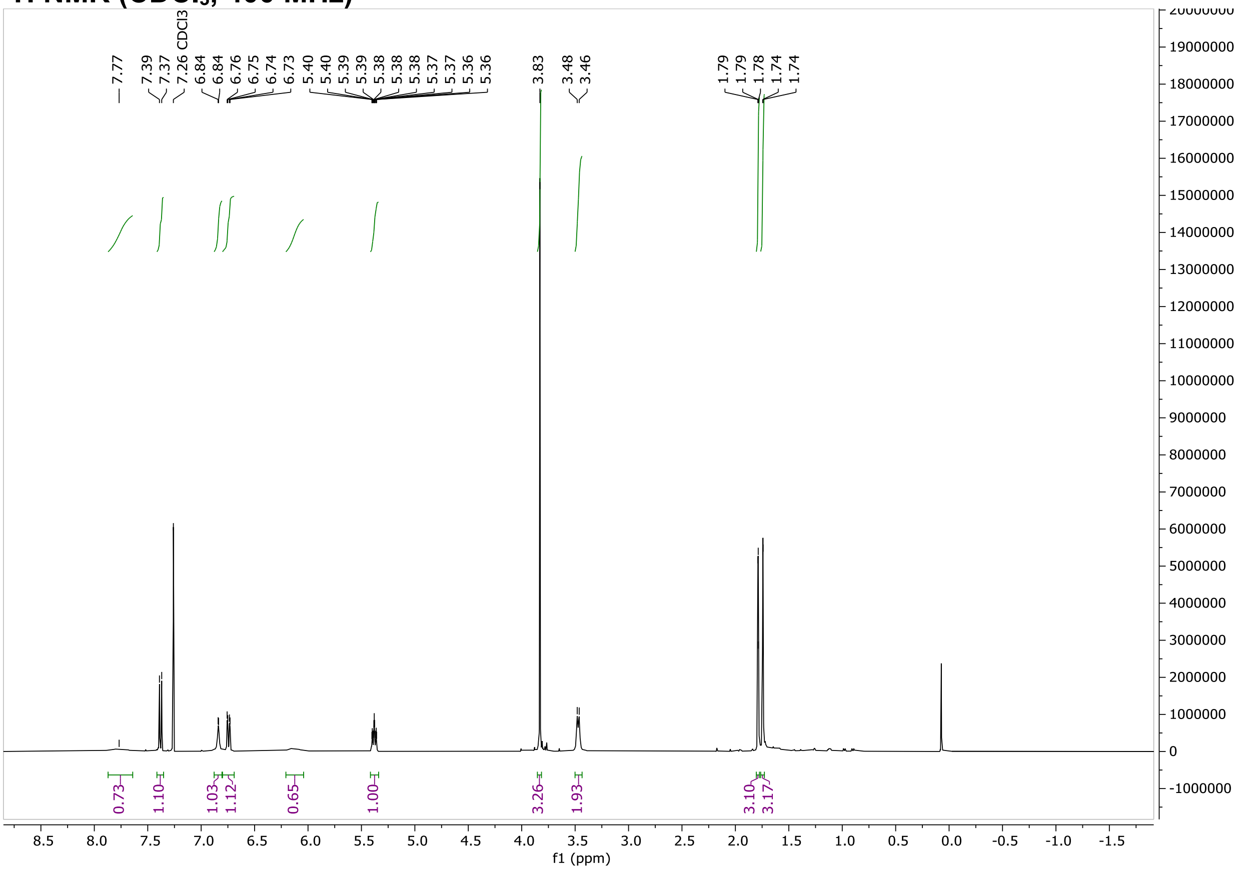

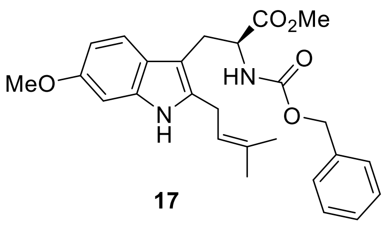

17

(1S,3S)-methyl 1-isobutyl-7-methoxy-2,3,4,9-tetrahydro-1H-pyrido[3,4-b]indole-3-carboxylate (17): To a solution of (*S*)-1-Benzyl 2-methyl aziridine-1,2-dicarboxylate (**16**) (25 mg, 0.11 mmol 1.00 eq.) and **15** (40 mg, 1.75 mmol, 1.75 eq.) in CH_2Cl_2 (0.5 ml) was added $Yb(OTf)_3$ (67 mg, 0.11 mmol, 1.00 eq., dried at at 150 °C overnight under vacuum) under argon. The mixture was stirred at room temperature for 20 h, water (3 ml) was added and the mixture was extracted with CH_2Cl_2 (3 x 3.5 ml). The combined organic layers were dried over $MgSO_4$, filtered and the solvent was evaporated *in vacuo*. The residue was purified by FC (hexane/AcOEt 5:1) to provide **17** as a yellow oil (46 mg, 96%). Upon addition of chloroform, the oil turned pink/red.

R_f = 0.44 (hexane/AcOEt 2:1); **$[\alpha]_D^{20}$**: +36.99 (c 1.0 in $CHCl_3$); **^{1}H NMR** (400 MHz, $CDCl_3$) δ (ppm) 7.69 (s, 1H), 7.38 – 7.24 (m, 6H), 6.78 (d, *J* = 2.2 Hz, 1H), 6.70 (dd, *J* = 8.6, 2.2 Hz, 1H), 5.29 (d, *J* = 8.3 Hz, 1H), 5.24 (tdd, *J* = 5.9, 3.0, 1.5 Hz, 1H), 5.11 (d, *J* = 12.2 Hz, 1H), 5.06 (d, *J* = 12.4 Hz, 1H), 4.65 (q, *J* = 5.9 Hz, 1H), 3.81 (s, 3H), 3.66 (s, 3H), 3.35 (d, *J* = 7.2 Hz, 2H), 3.22 (d, *J* = 5.7 Hz, 2H), 1.75 (s, 3H), 1.72 (s, 3H); **^{13}C NMR** (101 MHz, $CDCl_3$) δ (ppm) 172.7, 156.0, 155.8, 136.4, 136.0, 134.9, 134.8, 128.6, 128.2, 123.3, 120.5, 118.7, 109.1, 104.7, 94.7, 66.9, 55.8, 54.7, 52.5, 27.3, 25.8, 25.1, 17.9; **v_{max} (neat)/cm^{-1}** = 3358, 3034, 2950, 2834, 1710, 1629, 1503, 1462, 1439, 1350, 1284, 1245, 1212, 1159, 1062, 1027, 816, 751, 698; **HRMS** (ESI): m/z calcd for $C_{26}H_{31}N_2O_5$ $[M+H]^+$: 451.2227, found: 451.2234.

^{1}H NMR ($CDCl_3$, 400 MHz)

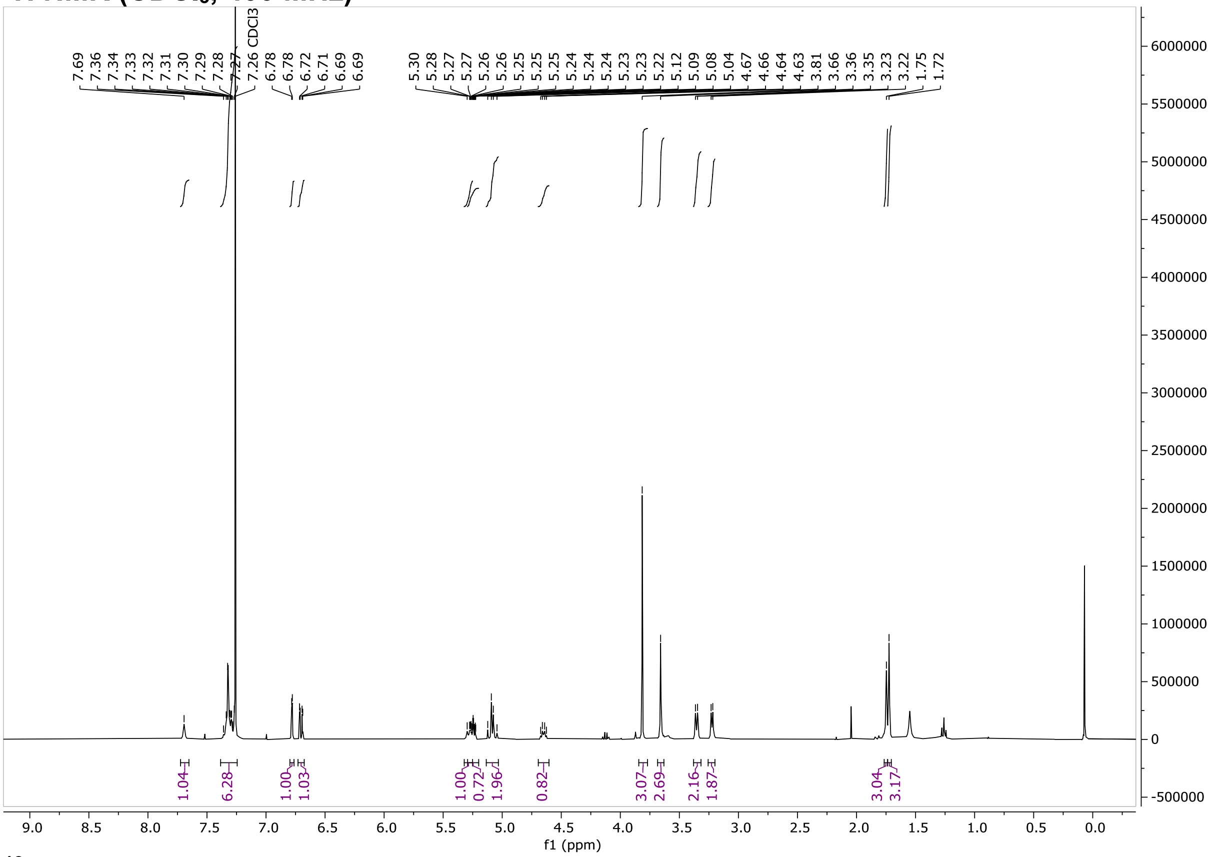

^{13}C NMR ($CDCl_3$, 101 MHz)

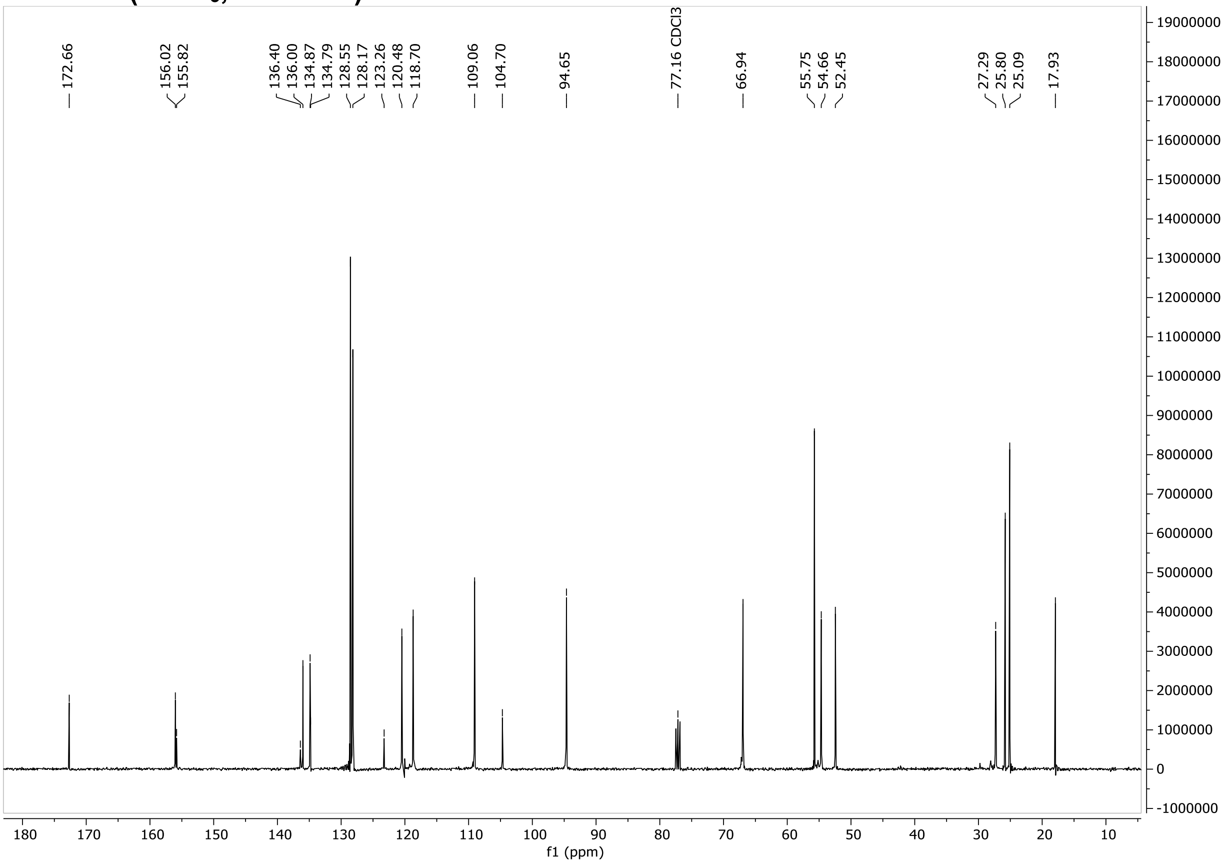

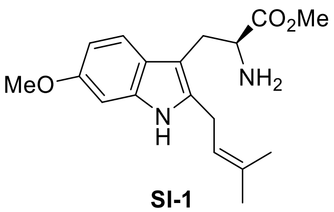

SI-1

Methyl (S)-2-amino-3-(6-methoxy-2-(3-methylbut-2-en-1-yl)-1H-indol-3-yl)propanoate (SI-1): A solution of Et_3SiH (71 µL, 0.44 mmol, 2.0 eq.), $Pd(OAc)_2$ (5 mg, 0.022 mmol, 0.1 eq.) and Et_3N (9 µL, 0.07 mmol, 0.3 eq.) in dry CH_2Cl_2 (1.6 mL) was stirred at rt and under argon for 15 min. To this mixture, a solution of **17** (100 mg, 0.22 mmol, 1.0 eq.) in dry CH_2Cl_2 (1.6 mL) was added and the mixture was stirred for 20 h. Sat. aq. NH_4Cl was added and the mixture was extracted with CH_2Cl_2 (3x). The combined organic layers washed with brine (2x), dried over MgSO4, filtered and evaporated. Purification of the residue by FC (CH_2Cl_2/MeOH 20:1) gave amino ester **SI-1** as a colorless oil (62.9 mg, 90%).

R_f = 0.09 (hexane/AcOEt 2:1); **$[\alpha]_D^{20}$**: +7.00 (c 1.0 in $CHCl_3$); **^{1}H NMR** (400 MHz, $CDCl_3$) δ 7.80 (s, 1H), 7.38 (d, *J* = 8.5 Hz, 1H), 6.80 (d, *J* = 2.1 Hz, 1H), 6.74 (dd, *J* = 8.6, 2.2 Hz, 1H), 5.28 (t, *J* = 7.5 Hz, 1H), 3.81 (s, 3H), 3.80 – 3.72 (m, 1H), 3.70 (s, 3H), 3.44 (d, *J* = 7.3 Hz, 2H), 3.20 (dd, *J* = 14.3, 5.1 Hz, 1H), 2.95 (dd, *J* = 14.3, 8.0 Hz, 1H), 1.77 (s, 3H), 1.75 (s, 3H); **^{13}C NMR** (101 MHz, $CDCl_3$) δ 175.8, 156.1, 136.1, 134.8, 123.3, 120.6, 118.9, 109.0, 106.1, 94.8, 55.8, 55.4, 52.2, 30.1, 25.9, 25.3, 18.0; **v_{max} (neat)/cm^{-1}** = 3371, 2951, 2916, 1731, 1628, 1568, 1499, 1462, 1438, 1376, 1347, 1244, 1200, 1157, 1101, 1027, 952, 819, 628, 441; **HRMS** (ESI): m/z calcd for $C_{18}H_{24}N_2Na_1O_3$ $[M+Na]^+$: 339.1679, found 339.1674.

^{1}H NMR ($CDCl_3$, 400 MHz)

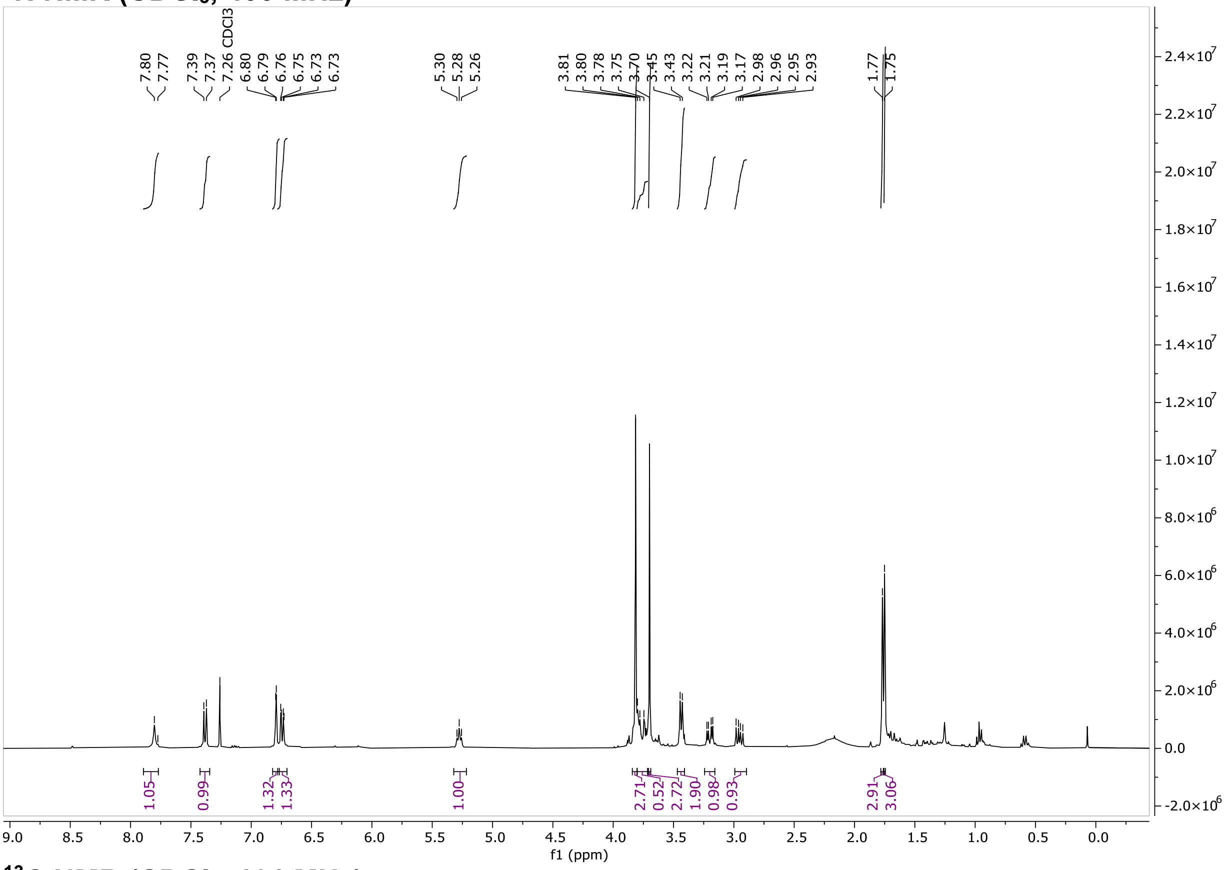

^{13}C NMR ($CDCl_3$, 101 MHz)

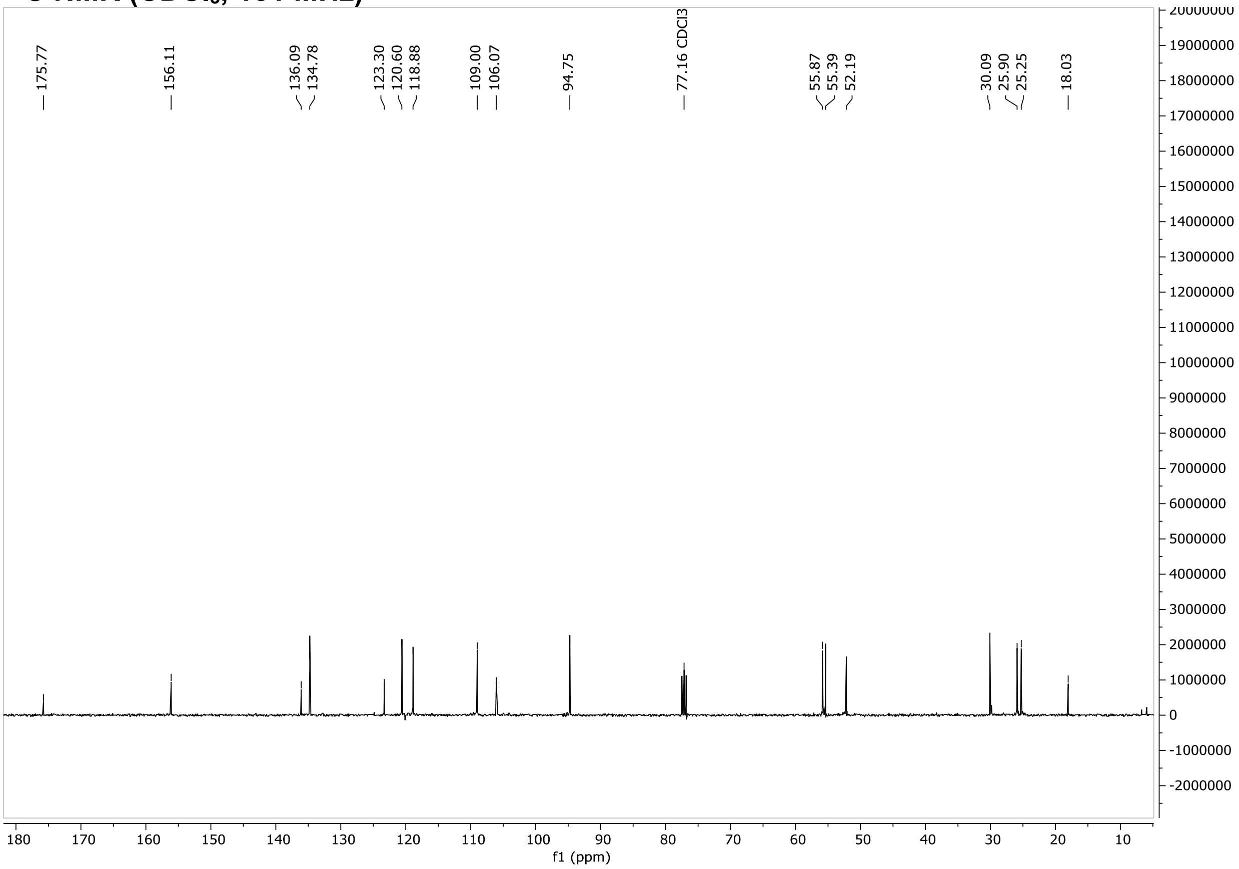

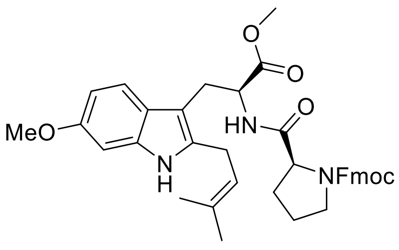

19

Dipeptide 19: A solution of Fmoc-L-Pro-OH (**18**) (30 mg, 0.09 mmol, 1.00 eq.) and **SI-1** (31 mg, 0.10 mmol, 1.10 eq.) in CH_2Cl_2 (0.7 ml) was stirred under argon at rt for 10 min. Then DMTMM (54 mg, 0.19 mmol, 2.20 eq) was added and the reaction mixture was stirred at rt for 24 h. Water was added and the mixture was extracted with CH_2Cl_2 (3 x). The combined organic layers were washed successively with sat. aq. $NaHCO_3$, water, 1M aq. HCl, water and brine, dried over $MgSO_4$, filtered and concentrated *in vacuo*. The residue was purified by FC (hexane/AcOEt 2:1) affording **19** as a white solid (27 mg, 48%). This material was used in the next step without full characterisation; due to the presence of rotamers, the NMR spectra of the compound were complex and uninformative.
On a 4 mg scale of **SI-1** dipeptide **19** had been obtained in 84% yield.

ν_{max} (neat)/cm^{-1} = 3378, 2979, 2936, 1691, 1516, 1457, 1394, 1369, 1306, 1253, 1220, 1164, 1116, 1068, 1002, 873, 850, 773, 669.

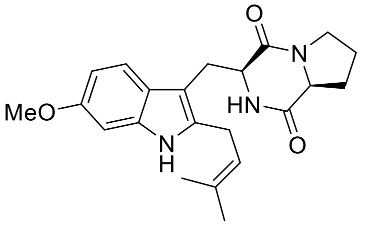

2

Tryprostatin A (4): Piperidine (0.06 mL, 0.65 mmol, 15.9 eq.) was added to a solution of **19** (26 mg, 0.04 mmol, 1.00 eq.) in dry CH_2Cl_2 (0.34 mL) and the solution was stirred under argon at rt for 1 h. The solution was concentrated *in vacuo* and the residue was re-dissolved in NH_3/MeOH (7 N, 0.7 mL) under argon, to promote cyclization. The solution was stirred overnight (ca. 16 h) and the volatiles were removed *in vacuo*. The residue was purified by FC (AcOEt) to give tryprostation A (**4**) as a white solid (8.7 mg, 56%).

R_f = 0.25 (AcOEt EE); **$[\alpha]_D^{20}$**: -61.24 (c 0.8 in $CHCl_3$; *lit*[40]*:* **$[\alpha]_D^{23}$**: -70 (c 0.78 in $CHCl_3$); **^{1}H NMR** (400 MHz, $CDCl_3$) δ 7.84 (s, 1H), 7.34 (d, *J* = 8.6 Hz, 1H), 6.83 (d, *J* = 2.2 Hz, 1H), 6.76 (dd, *J* = 8.6, 2.3 Hz, 1H), 5.63 (s, 1H), 5.30 (tt, *J* = 7.1, 1.5 Hz, 1H), 4.33 (dd, *J* = 11.8, 3.7 Hz, 1H), 4.06 (t, *J* = 8.1 Hz, 1H), 3.83 (s, 3H), 3.72 – 3.54 (m, 3H), 3.43 (t, *J* = 7.1 Hz, 2H), 2.91 (dd, *J* = 15.1, 11.4 Hz, 1H), 2.38 – 2.28 (m, 1H), 2.09 – 1.98 (m, 2H), 1.96 – 1.86 (m, 1H), 1.78 (bs, 3H), 1.75 (bs, 3H); **^{13}C NMR** (101 MHz, $CDCl_3$) δ 169.5, 166.0, 156.5, 136.4, 135.4, 135.2, 122.4, 120.1, 118.5, 109.5, 104.6, 95.0, 59.4, 55.9, 54.7, 45.6, 28.5, 25.9, 25.8, 25.2, 22.8, 18.1; **ν_{max} (neat)/cm^{-1}** 3662, 2987, 2972, 2901, 1670, 1451, 1407, 1394, 1382, 1251, 1242, 1229, 1075, 1066, 1056, 893, 880, 870, 744, 436; **HRMS** (ESI): m/z calcd for $C_{22}H_{27}N_3Na_1O_3$ $[M+Na]^+$: 404.1945, found: 404.1938.
The spectral data match with those reported by Yamakawa *et al.* [40]

^{1}H NMR ($CDCl_3$, 400 MHz)

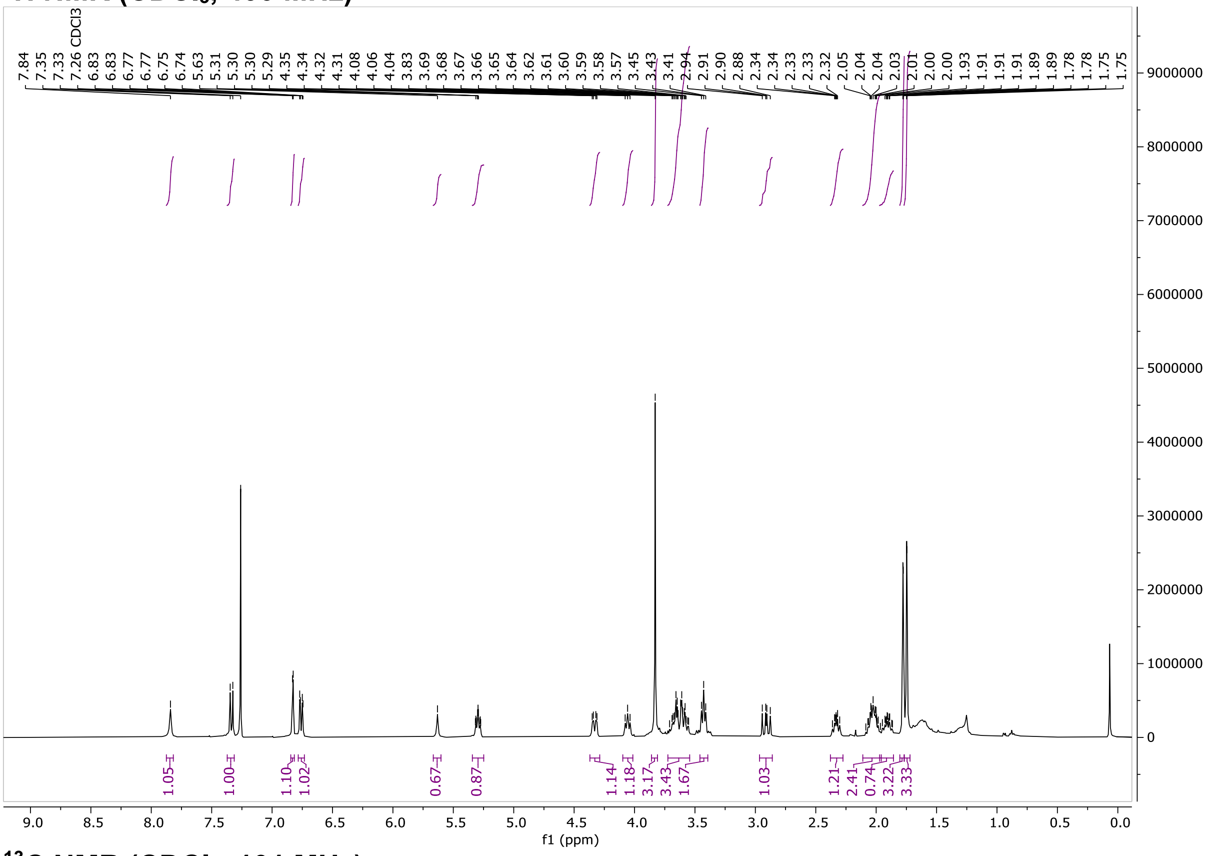

^{13}C NMR ($CDCl_3$, 101 MHz)

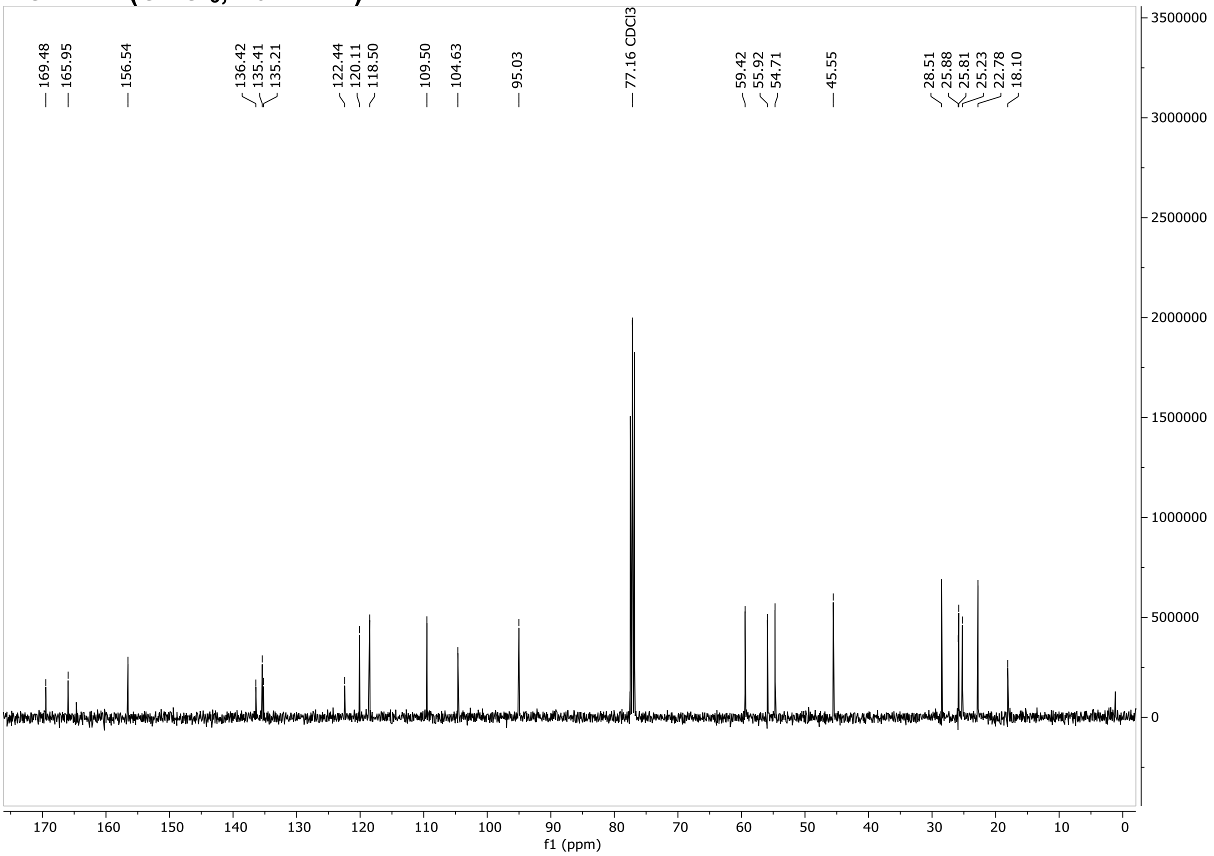

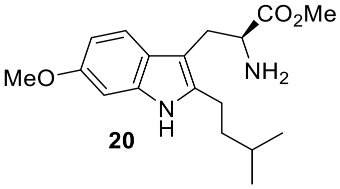

Methyl (S)-2-amino-3-(6-methoxy-1H-indol-3-yl)propanoate (20): To a solution of **17** (1.30 g, 2.89 mmol, 1.00 eq.) in MeOH (55 ml) was added Pd/C (10% w/w, 260 mg, 0.32 mmol, 0.11 eq.) under argon. The flask was flushed with hydrogen and the mixture stirred at rt for 3 h. Hydrogen was then replaced by argon and the mixture was filtered over Celite. The solvent was removed under reduced pressure and the residue was purified by FC (CH_2Cl_2 to CH_2Cl_2/MeOH 95:5) to give the free amino ester **20** (850 mg, 92%) as a yellow oil.

R_f = 0.63 (AcOEt); **$[\alpha]_D^{20}$**: +5.00 (c 1.0 in $CHCl_3$); **^{1}H NMR** (400 MHz, $CDCl_3$) δ (ppm) 7.72 (bs, 1H), 7.39 (d, *J* = 8.5, 1H), 6.80 (d, *J* = 2.2 Hz, 1H), 6.75 (dd, *J* = 8.6, 2.3 Hz, 1H), 3.85 – 3.80 (m, 1H), 3.83 (s, 3H), 3.71 (s, 3H), 3.20 (dd, *J* = 14.5, 5.1 Hz, 1H), 2.93 (dd, *J* = 14.4, 8.3 Hz, 1H), 2.77 – 2.65 (m, 2H), 1.62 (dp, *J* = 12.6, 6.0 Hz, 1H), 1.56 – 1.47 (m, 2H), 0.95 (d, *J* = 6.5 Hz, 6H); **^{13}C NMR** (101 MHz, $CDCl_3$) δ (ppm) 175.5, 156.1, 136.5, 136.2, 123.0, 118.9, 108.9, 105.8, 94.8, 55.9, 55.3, 52.3, 39.1, 29.8, 28.0, 24.2, 22.6; **v_{max} (neat)/cm^{-1}** = 3683, 3675, 3662, 2987, 2972, 2901, 1451, 1407, 1394, 1382, 1251, 1242, 1229, 1075, 1066, 1056, 1028, 985, 892, 879, 871, 417; **HRMS** (ESI): m/z calcd for $C_{18}H_{26}N_2Na_1O_3$ [M+Na]+: 341.1836, found: 341.1828.

^{1}H NMR ($CDCl_3$, 400 MHz)

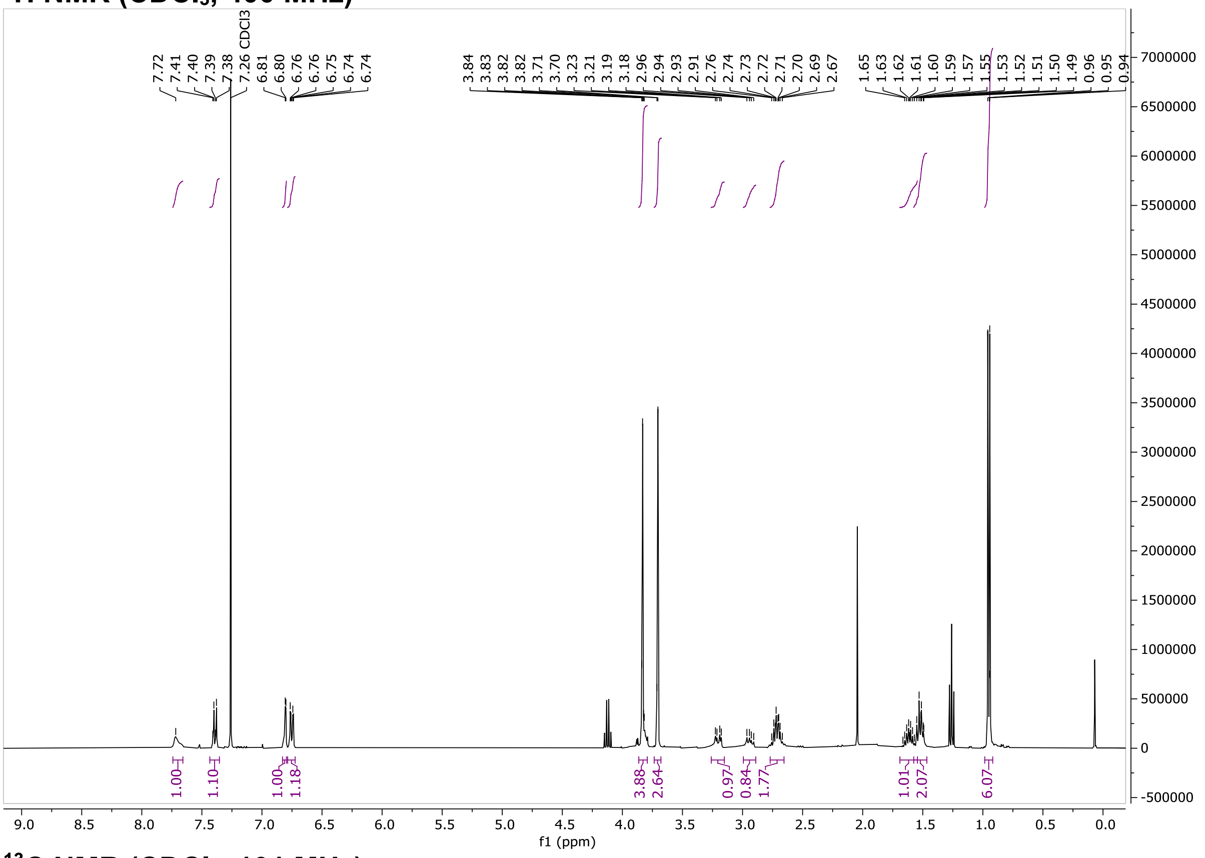

^{13}C NMR ($CDCl_3$, 101 MHz)

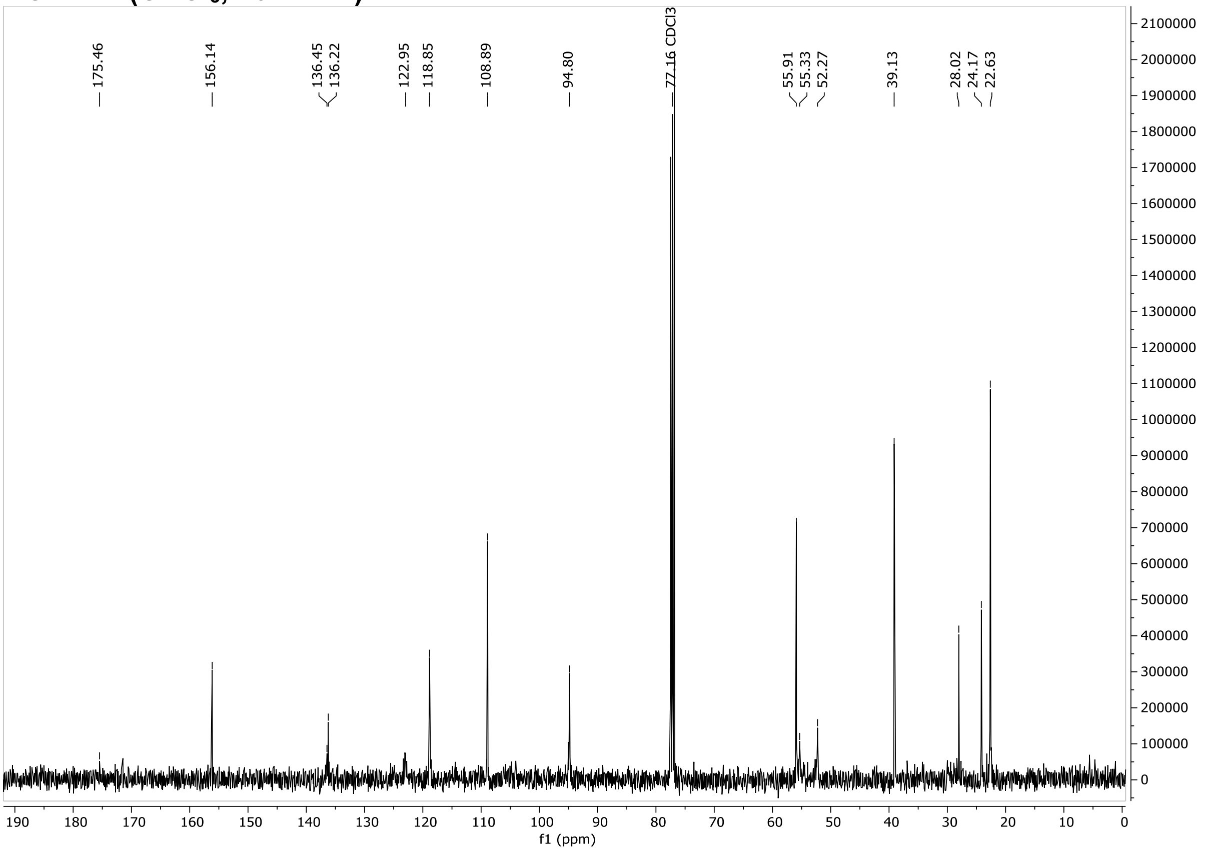

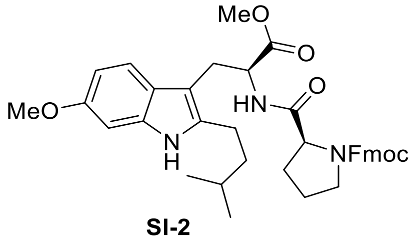

SI-2

Dipeptide ester SI-2: A solution of Fmoc-Pro-OH (**18**) (50 mg, 0.15 mmol, 1.00 eq.) and **20** (51.9 mg, 0.16 mmol, 1.10 eq.) in CH_2Cl_2 (0.7 ml) was stirred at rt for 10 min. Then DMTMM (90.2 mg, 0.33 mmol, 2.20 eq.) was added and the solution was stirred at rt for 24 h. Water was added and the mixture was extracted with CH_2Cl_2 (3x). The combined organic layers were washed successively with sat. $NaHCO_3$, water, 1M HCl, water and brine, dried over $MgSO_4$, filtered and concentrated *in vacuo*. The residue was purified by FC (hexane/AcOEt 2:1) to afford **SI-2** as a white solid (72 mg, 76%). This material was used in the next step without full characterisation; due to the presence of rotamers, the NMR spectra of the compound were complex and uninformative.

R_f = 0.3 (hexane/AcOEt 2:1); **mp** = 78 – 90 °C; **$[\alpha]_D^{20}$**: -25.00 (c 1.0 in $CHCl_3$); **ν_{max} (neat)/cm^{-1}** = 3330, 2953, 1741, 1680, 1629, 1574, 1541, 1519, 1469, 1450, 1419, 1364, 1309, 1245, 1200, 1160, 1120, 1091, 1031, 987, 817, 802, 756, 742, 665, 544; **HRMS** (ESI): m/z calcd for $C_{38}H_{44}N_3O_6$ $[M+H]^+$: 638.3225, found: 638.3230.

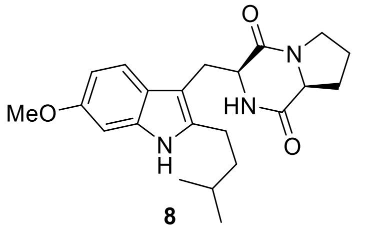

8

(3S,8aS)-3-((2-isopentyl-6-methoxy-1H-indol-3-yl)methyl)hexahydropyrrolo[1,2-a]pyrazine-1,4-dione (8): Piperidine (46 µL, 0.46 mmol, 15.9 eq.) was added to a solution of **SI-2** (18.5 mg, 0.03 mmol, 1.00 eq.) in dry CH_2Cl_2 (0.24 mL) and the reaction mixture was stirred under argon at rt for 1 h. The solution was concentrated *in vacuo* nd the residue was re-dissolved with NH_3/MeOH (7 N, 0.44 mL) under argon to promote cyclization. The solution was stirred overnight under argon at rt (ca. 16 h). The volatiles were removed *in vacuo* and the residue was purified by FC (AcOEt) to give **8** as a white solid (5.0 mg, 72%).

R_f = 0.28 (AcOEt); **$[\alpha]_D^{20}$**: -82.98 (c 1.0 in $CHCl_3$); **^{1}H NMR** (400 MHz, $CDCl_3$) δ (ppm) 7.89 (bs, 1H), 7.33 (d, *J* = 8.6 Hz, 1H), 6.82 (d, *J* = 2.2 Hz, 1H), 6.76 (dd, *J* = 8.6, 2.2 Hz, 1H), 5.60 (s, 1H), 4.35 (dd, *J* = 11.3, 2.7 Hz, 1H), 4.07 (t, *J* = 8.1 Hz, 1H), 3.83 (s, 3H), 3.72 – 3.57 (m, 3H), 2.89 (dd, *J* = 15.1, 11.5 Hz, 1H), 2.69 (td, *J* = 7.5, 3.8 Hz, 2H), 2.39 – 2.29 (m, 1H), 2.14 – 1.97 (m, 2H), 1.97 – 1.87 (m, 1H), 1.60 (dp, *J* = 12.9, 6.5 Hz, 1H), 1.58 – 1.47 (m, 2H), 0.94 (d, *J* = 6.3 Hz, 6H); **^{13}C NMR** (101 MHz, $CDCl_3$) δ 169.5, 166.0, 156.5, 136.8, 136.5, 122.2, 118.5, 109.4, 104.7, 95.0, 59.4, 55.9, 54.6, 45.6, 39.2, 28.5, 28.0, 25.8, 24.2, 22.8, 22.6, 22.5; **ν_{max} (neat)/cm^{-1}** = 3303, 2954, 2870, 1660, 1463, 1424, 1305, 1253, 1201, 1160, 1119, 1104, 1032, 819, 753, 665; **HRMS** (ESI): m/z calcd for $C_{22}H_{29}N_3Na_1O_3$ $[M+Na]^+$: 406.21.01, found: 406.2103.

^{1}H NMR ($CDCl_3$, 400 MHz)

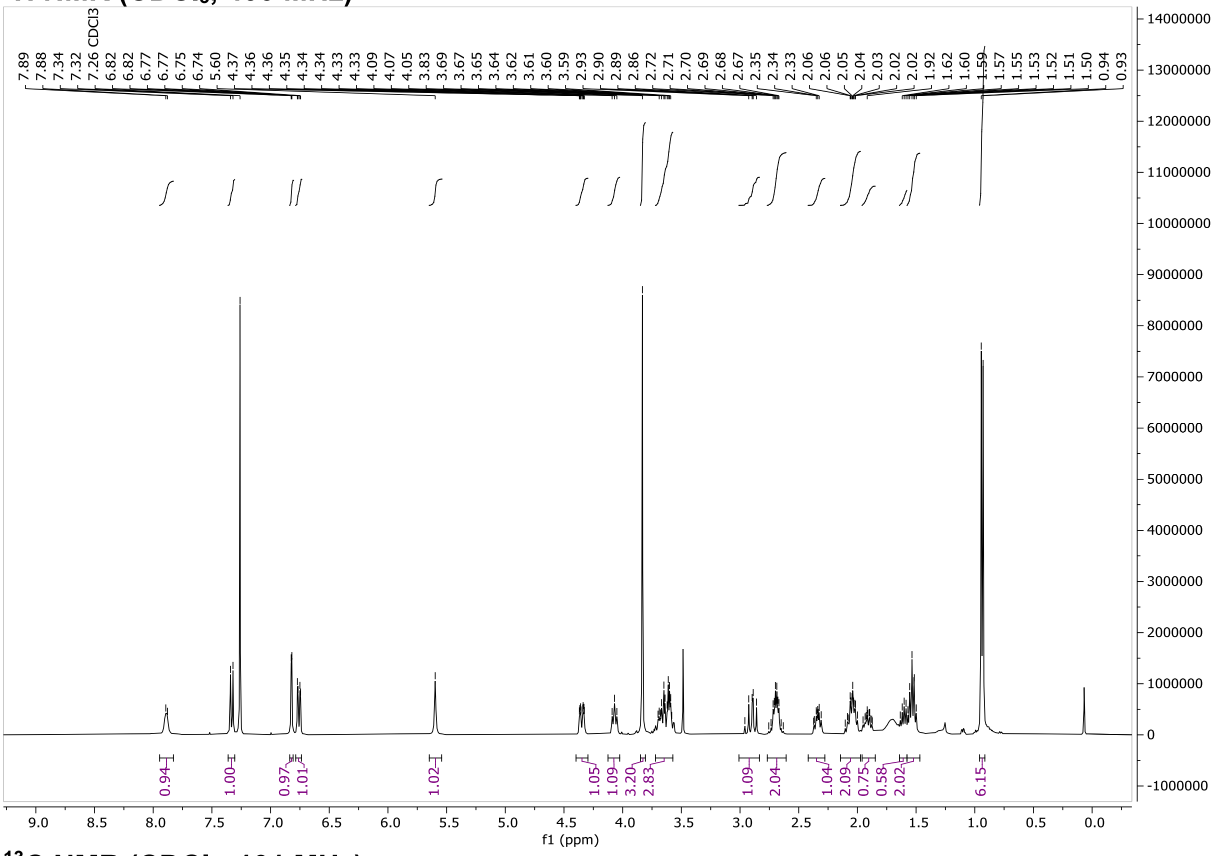

^{13}C NMR ($CDCl_3$, 101 MHz)

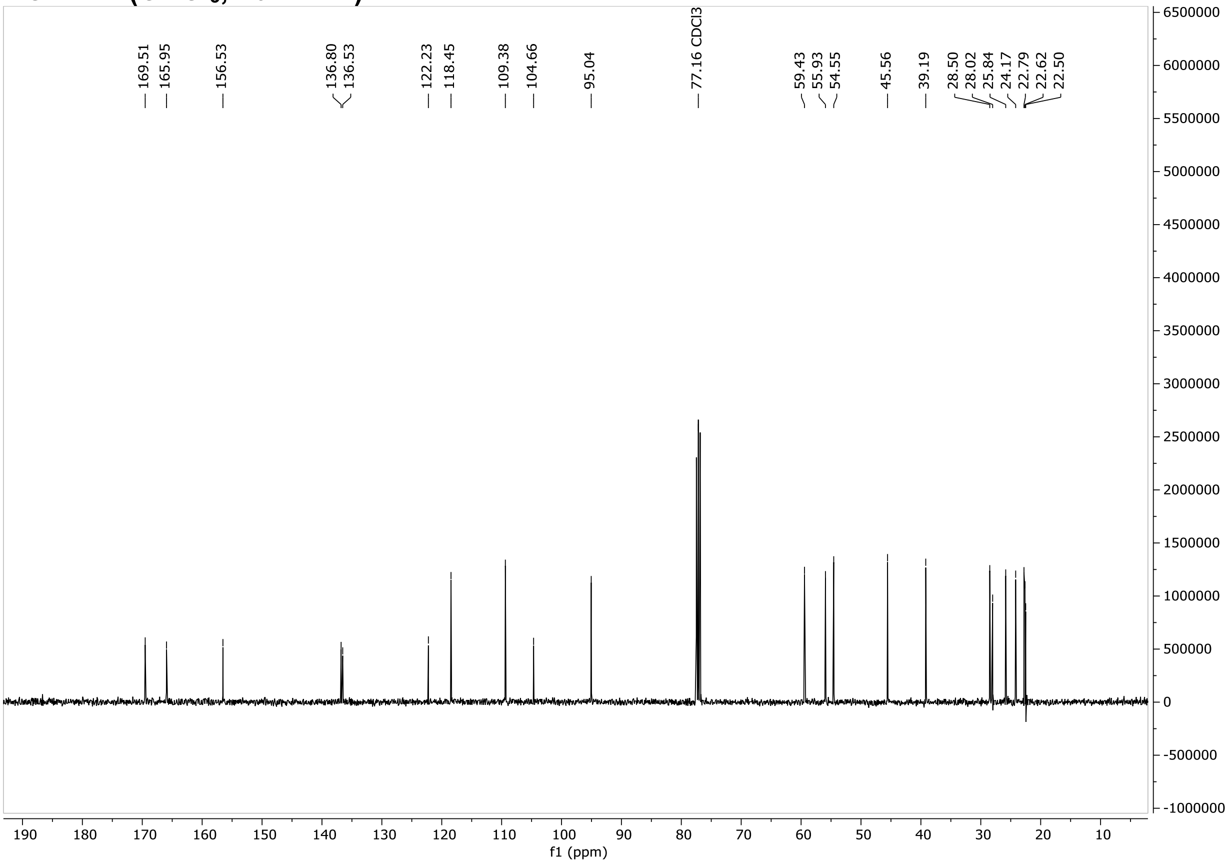

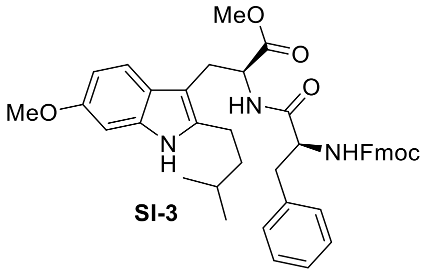

SI-3

Methyl (S)-2-((R)-2-((((9H-fluoren-9-yl)methoxy)carbonyl)amino)-3-phenylpropanamido)-3-(2-isopentyl-6-methoxy-1H-indol-3-yl)propanoate (SI-3): A solution of Fmoc-Phe-OH (40 mg, 0.10 mmol, 1.00 eq.) and **20** (36.2 mg, 0.11 mmol, 1.10 eq.) in CH_2Cl_2 (0.5 ml) was stirred at rt for 10 min. Then DMTMM (62.9 mg, 0.227 mmol, 2.20 eq.) was added and the solution was stirred at rt for 17 h. Water was added and the mixture was extracted with CH_2Cl_2 (3 x). The combined organic layers were washed successively with sat. aq. $NaHCO_3$, water, 1M aq. HCl, water and brine, dried over $MgSO_4$, filtered and evaporated *in vacuo*. The residue was purified by FC (hexane/AcOEt 2:1) to afford **SI-3** as a white solid (62 mg, 87%).
The product was used in the next step without full characterisation.

R_f = 0.36 (hexane/AcOEt 2:1); **$[\alpha]_D^{20}$**: +4.00 (c 0.5 in $CHCl_3$); **v_{max} (neat)/cm^{-1}** = 3324, 2953, 2927, 1714, 1660, 1630, 1500, 1450, 1323, 1247, 1219, 1160, 1108, 1032, 771, 742, 701; **HRMS** (ESI): m/z calcd for $C_{42}H_{45}N_3Na_1O_6$ $[M+Na]^+$: 710.3201, found: 710.3210.

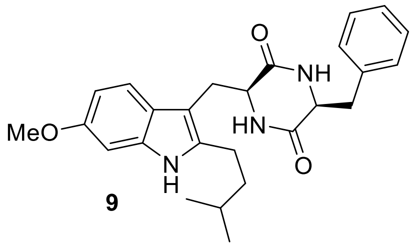

9

(3S,6S)-3-Benzyl-6-((2-isopentyl-6-methoxy-1H-indol-3-yl)methyl)piperazine-2,5-dione (9): Piperidine (0.11 mL, 1.16 mmol, 15.9 eq.) was added to a solution of **S-3** (50mg, 0.07 mmol, 1.00 eq.) in dry CH_2Cl_2 (0.6 mL) and the mixture was stirred under argon at rt for 1 h. The solution was concentrated *in vacuo* and the residue was re-dissolved in NH_3/MeOH (7 N, 1.35 mL) to promote cyclization. The reaction mixture was stirred overnight under argon at rt (ca. 16 h). The volatiles were removed *in vacuo* and the residue was purified by FC (AcOEt) to give **9** as a white solid (10.0 mg, 32%).

R_f = 0.34 (AcOEt); **mp** = 290 – 293 °C; **$[\alpha]_D^{20}$**: -20.00 (c 0.3 in $CHCl_3$); **^{1}H NMR** (400 MHz, DMSO-d_6) δ (ppm) 10.60 (s, 1H), 7.94 (s, 1H), 7.54 (s, 1H), 7.32 (d, J = 8.7 Hz, 1H), 7.15 – 7.07 (m, 3H), 6.73 (s, 1H), 6.61 (d, J = 8.6 Hz, 1H), 6.56 – 6.46 (m, 2H), 4.00 – 3.90 (m, 1H), 3.73 (s, 3H), 3.69 (bs, 1H), 2.84 – 2.78 (m, 2H), 2.73 – 2.52 (m, 2H), 2.38 (dd, J = 13.5, 4.3 Hz, 1H), 1.59 – 1.47 (m, 3H), 1.42 (dd, J = 13.2, 8.7 Hz, 1H), 0.93 (d, J = 5.7 Hz, 6H); **^{13}C NMR** (101 MHz, DMSO-d_6) δ (ppm) 166.9, 166.0, 155.1, 137.2, 136.8, 136.0, 129.6, 127.9, 126.3, 123.1, 119.1, 108.0, 104.2, 94.2, 56.0, 55.9, 55.3, 40.6, 38.1, 29.0, 27.5, 23.5, 22.6, 22.4; **v_{max} (neat)/cm^{-1}** = 3050, 2364, 1661, 1453, 1323, 1158, 837, 810, 702; **HRMS** (ESI): m/z calcd for $C_{26}H_{31}N_3Na_1O_3$ $[M+Na]^+$: 456.2258, found: 456.2256.

^{1}H NMR (DMSO-d_6, 400 MHz)

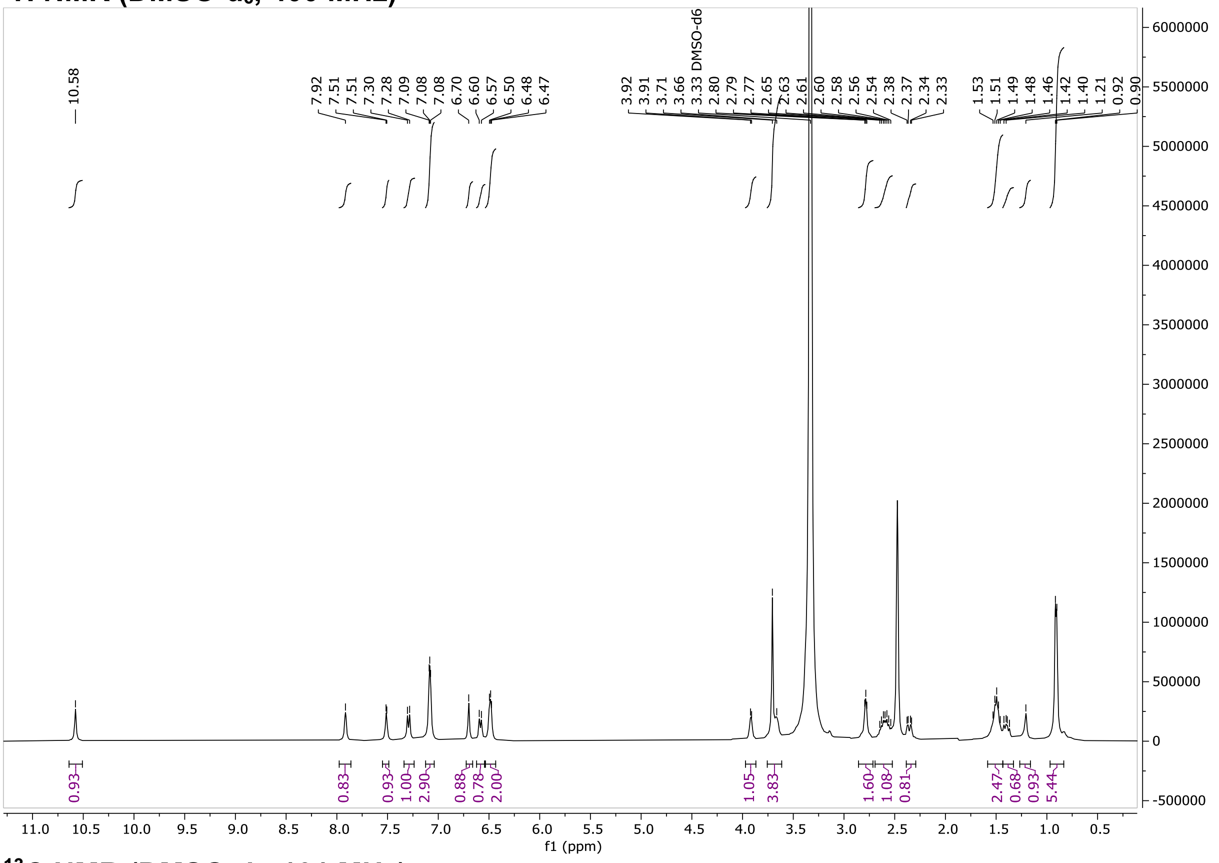

^{13}C NMR (DMSO-d_6, 101 MHz)

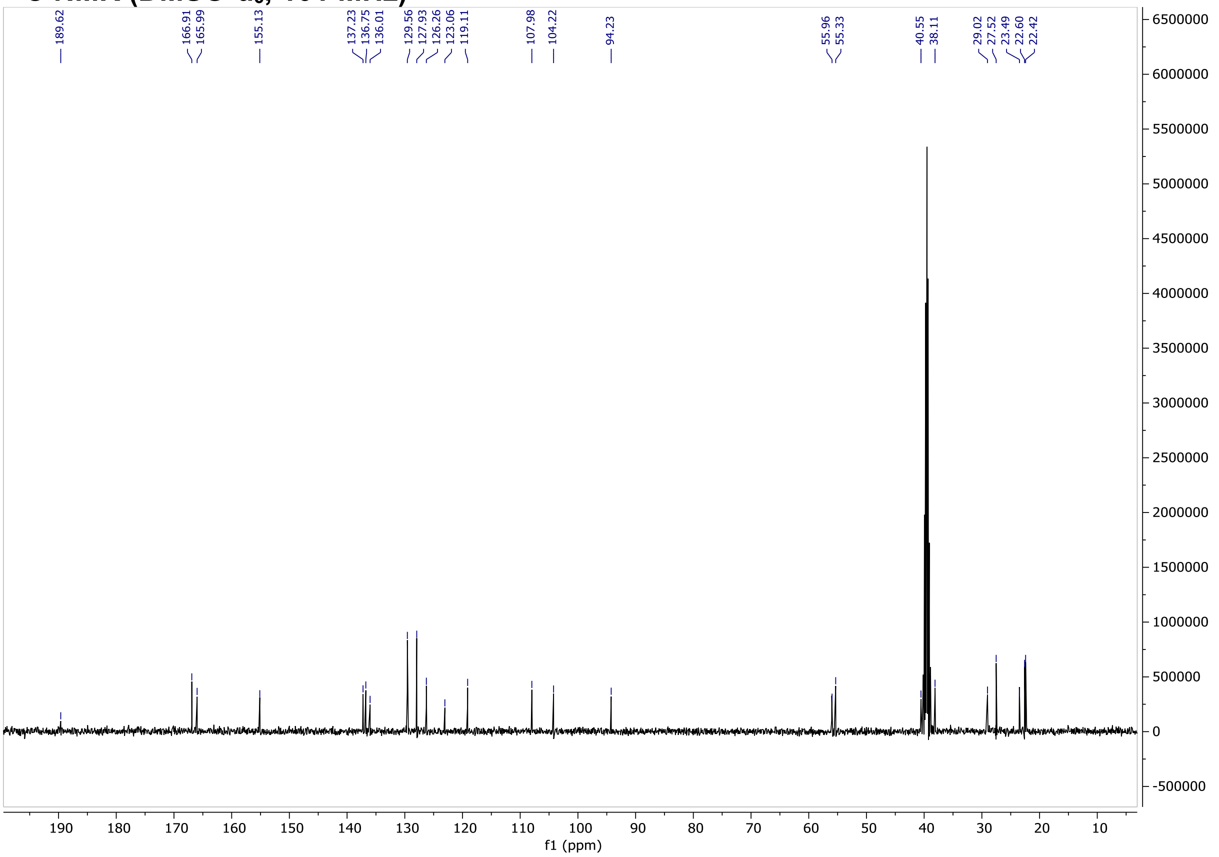

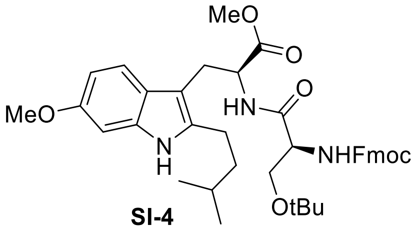

SI-4

Methyl (S)-2-((R)-2-((((9H-fluoren-9-yl)methoxy)carbonyl)amino)-3-(tert-butoxy)propanamido)-3-(2-isopentyl-6-methoxy-1H-indol-3-yl)propanoate (SI-4): A solution of Fmoc-Ser(tBu)-OH (40 mg, 0.10 mmol, 1.00 eq.) and **20** (36.5 mg, 0.12 mmol, 1.10 eq.) in CH_2Cl_2 (0.68 ml) was stirred at rt for 10 min. Then DMTMM (63.5 mg, 0.23 mmol, 2.20 eq.) was added and the solution was stirred at rt for 17 h. Water was added, and the mixture was extracted with CH_2Cl_2 (3 x). The combined organic layers were washed successively with sat. $NaHCO_3$, water, 1M HCl, water and brine, dried over $MgSO_4$, filtered and evaporated *in vacuo*. The residue was purified by FC (hexane/AcOEt 2:1) to afford **SI-4** as a white solid (61.5 mg, 86%).
The product was used in the next step without full characterisation.

R_f = 0.43 (hexane/AcOEt 2:1); **v_{max} (neat)/cm^{-1}** = 3336, 2954, 1726, 1667, 1630, 1500, 1464, 1450, 1393, 1365, 1323, 1275, 1240, 1213, 1199, 1160, 1121, 1104, 1084, 1033, 816, 759, 739, 702, 621, 544; **HRMS** (ESI): m/z calcd for $C_{40}H_{49}N_3Na_1O_7$ $[M+Na]^+$: 706.3463, found: 706.3456.

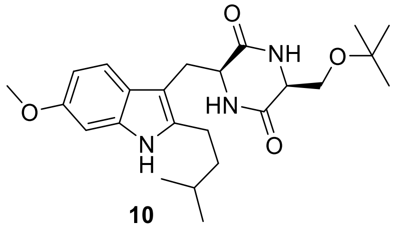

10

(3S,6S)-3-(tert-Butoxymethyl)-6-((2-isopentyl-6-methoxy-1H-indol-3-yl)methyl)piperazine-2,5-dione (10): Piperidine (115 µL, 1.16 mmol, 15.9 eq.) was added to a solution of **SI-4** (50 mg, 0.07 mmol, 1.00 eq.) in dry CH_2Cl_2 (0.6 mL) and the solution was stirred under argon at rt for 1 h. The solution was concentrated *in vacuo* and the residue was re-dissolved in NH_3/MeOH (7 N, 1.26 mL) to promote cyclization. The solution was stirred overnight under argon at rt (ca. 16 h). The volatiles were removed *in vacuo* and the residue was purified by FC (AcOEt) to give **10** as a white solid (11.1 mg, 35%).

R_f = 0.40 (AcOEt); **mp** = 291 – 293 °C; **$[\alpha]_D^{20}$**: -65.61 (c 0.32 in $CHCl_3$); **^{1}H NMR** (400 MHz, DMSO-d_6) δ (ppm) 10.56 (s, 1H), 7.86 (d, J = 2.7 Hz, 1H), 7.76 (d, J = 3.0 Hz, 1H), 7.30 (d, J = 8.5 Hz, 1H), 6.74 (d, J = 2.3 Hz, 1H), 6.56 (dd, J = 8.6, 2.4 Hz, 1H), 3.82 (dt, J = 7.3, 3.7 Hz, 1H), 3.72 (s, 3H), 3.67 (dt, J = 5.6, 3.0 Hz, 1H), 3.21 – 3.14 (m, 1H), 3.15 – 3.01 (m, 2H), 2.81 (dd, J = 9.1, 5.0 Hz, 1H), 2.67 – 2.58 (m, 2H), 1.59 – 1.48 (m, 3H), 1.01 (s, 9H), 0.92 (d, J = 5.9 Hz, 6H); **^{13}C NMR** (101 MHz, DMSO-d_6) δ (ppm) 167.2, 165.0, 154.9, 136.8, 136.0, 122.9, 118.6, 107.8, 104.5, 94.2, 72.8, 63.7, 56.1, 55.8, 55.2, 38.3, 30.5, 27.6, 27.1, 23.6, 22.4; **v_{max} (neat)/cm^{-1} =** 3367, 3187, 3049, 2955, 2927, 2871, 1667, 1628, 1499, 1461, 1365, 1329, 1256, 1198, 1159, 1102, 1022, 927, 838, 810, 743; **HRMS** (ESI): m/z calcd for $C_{24}H_{35}N_3Na_1O_4$ $[M + Na]^+$: 452.2520, found: 452.2521.

^{1}H NMR (DMSO-d_6, 400 MHz)

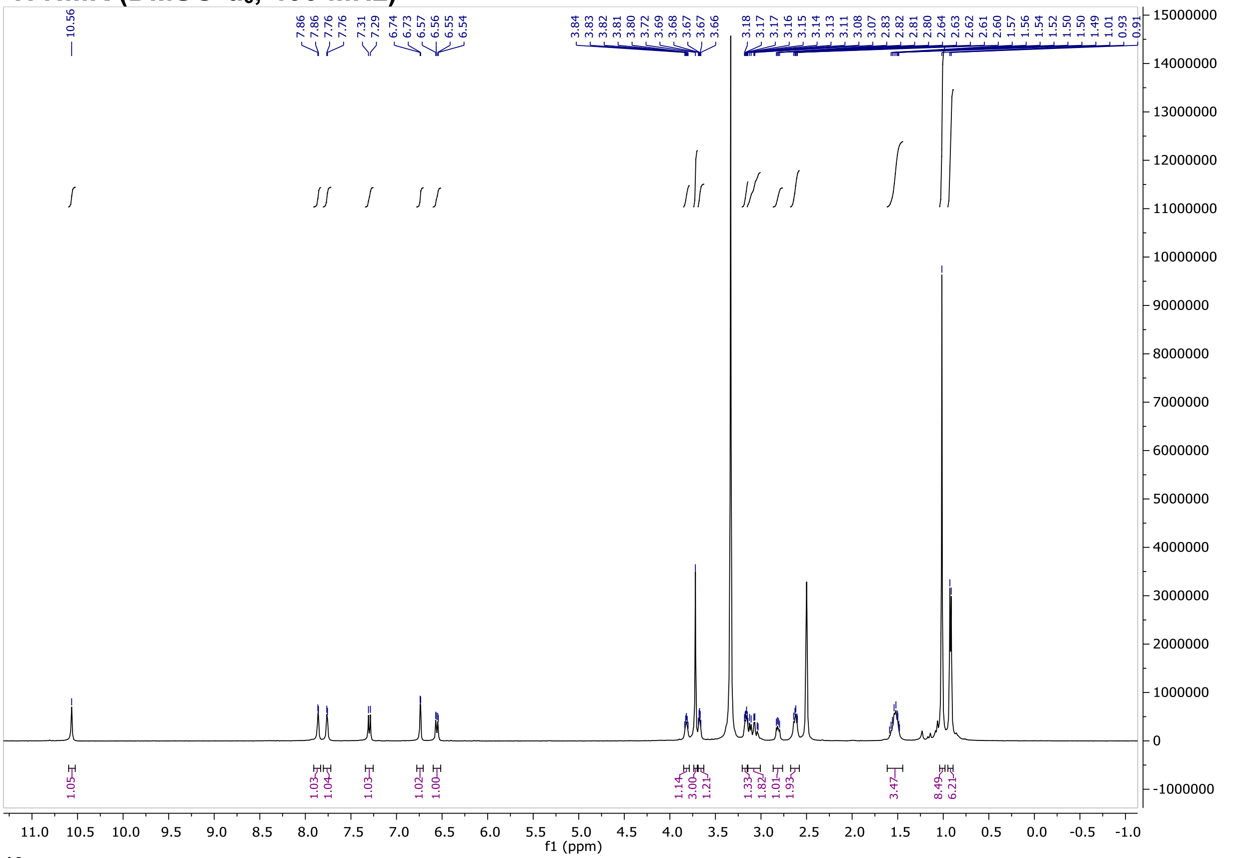

^{13}C NMR (DMSO-d_6, 101 MHz)

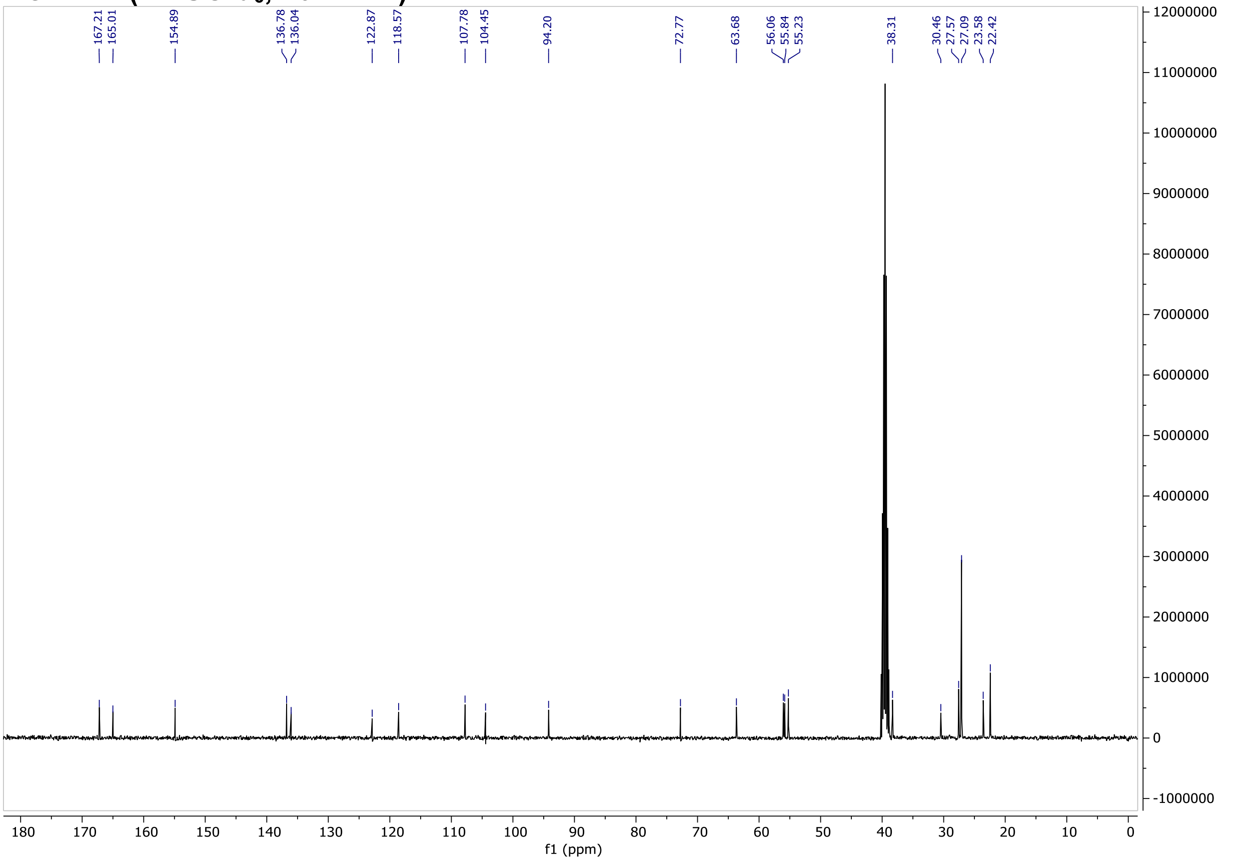

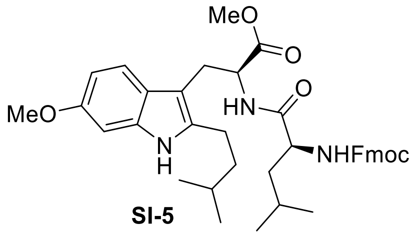

SI-5

Methyl (S)-2-((R)-2-((((9H-fluoren-9-yl)methoxy)carbonyl)amino)-4-methylpentanamido)-3-(2-isopentyl-6-methoxy-1H-indol-3-yl)propanoate (SI-5): A solution of Fmoc-Leu-OH (40 mg, 0.11 mmol, 1.00 eq.) and **20** (39.6 mg, 0.13 mmol, 1.10 eq.) in CH_2Cl_2 (0.51 ml) was stirred at rt for 10 min. Then DMTMM (68.9 mg, 0.25 mmol, 2.20 eq.) was added and the solution was stirred at rt for 17 h. Water was added and the mixture was extracted with CH_2Cl_2 (3 x). The combined organic layers were washed successively with sat. aq. $NaHCO_3$, water, 1M aq. HCl, water and brine, dried over MgSO4, filtered and evaporated *in vacuo*. The residue was purified by FC (hexane/AcOEt 2:1) to affording **SI-5** as a white solid (60 mg, 81%).
The product was used in the next step without full characterisation.

R_f = 0.39 (hexane/AcOEt 2:1); **$[\alpha]_D^{20}$**: +16.00 (c 0.5 in $CHCl_3$); **ν_{max} (neat)/cm^{-1}** = 3334, 2954, 1711, 1661, 1631, 1505, 1465, 1449, 1366, 1324, 1247, 1219, 1201, 1160, 1109, 1032, 771, 741; **HRMS** (ESI): m/z calcd for $C_{39}H_{47}N_3Na_1O_6$ $[M+Na]^+$: 676.3357, found: 676.3358.

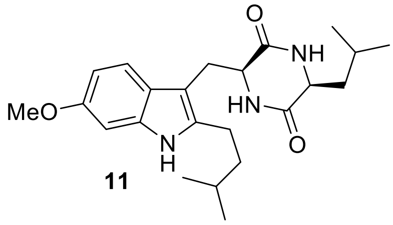

11

(3S,6S)-3-isobutyl-6-((2-isopentyl-6-methoxy-1H-indol-3-yl)methyl)piperazine-2,5-dione (11): Piperidine (0.12 mL, 1.22 mmol, 15.9 eq.) was added to a solution of **SI-5** (50 mg, 0.08 mmol, 1.00 eq.) in dry CH_2Cl_2 (0.6 mL) and the mixture was stirred under argon at rt for 1 h. The solution was concentrated *in vacuo* and the residue was re-dissolved in NH_3/MeOH (7 N, 1.82 mL) to promote cyclization. The solution was then stirred overnight under argon at rt (ca. 16 h). The volatiles were removed *in vacuo* and the residue was purified by FC (AcOEt) to give **11** as a white solid (21.5 mg, 70%).

R_f = 0.39 (AcOEt); **mp** = 240 – 241.7 °C; $[\alpha]_D^{20}$: +5.00 (c 0.5 in $CHCl_3$); **^{1}H NMR** (400 MHz, DMSO-d_6) δ (ppm) 10.57 (s, 1H), 7.80 (d, *J* = 3.0 Hz, 1H), 7.66 (s, 1H), 7.31 (d, *J* = 8.6 Hz, 1H), 6.72 (d, *J* = 2.3 Hz, 1H), 6.53 (dd, *J* = 8.6, 2.3 Hz, 1H), 3.93 (dd, *J* = 4.8, 4.2 Hz, 1H), 3.71 (s, 3H), 3.16 (dd, *J* = 14.5, 4.2 Hz, 1H), 2.92 (dd, *J* = 14.5, 4.8 Hz, 1H), 2.71 – 2.57 (m, 2H), 2.54 (dd, *J* = 7.1, 4.6 Hz, 1H), 1.65 – 1.53 (m, 1H), 1.53 – 1.44 (m, 3H), 1.37 (ddd, *J* = 13.3, 8.2, 4.7 Hz, 1H), 1.17 (dt, *J* = 13.6, 6.6 Hz, 1H), 0.91 (d, *J* = 5.7 Hz, 6H), 0.65 (d, *J* = 6.6 Hz, 3H), 0.52 (d, *J* = 6.6 Hz, 3H); **^{13}C NMR** (101 MHz, DMSO-d_6) δ (ppm) 169.2, 168.3, 154.8, 137.3, 136.1, 122.6, 118.7, 108.0, 103.7, 93.9, 56.2, 55.2, 50.9, 39.7, 38.1, 28.7, 27.4, 23.4, 23.2, 22.6, 22.5, 22.3, 21.5; **ν_{max} (neat)/cm^{-1}** = 3387, 3195, 3057, 2954, 2870, 1667, 1628, 1567, 1498, 1454, 1386, 1366, 1322, 1259, 1202, 1163, 1149, 1110, 1023, 823, 800, 771, 470, 446; **HRMS** (ESI): m/z calcd for $C_{23}H_{33}N_3Na_1O_3$ $[M+Na]^+$: 422.2414, found: 422.2407.

^{1}H NMR (DMSO-d_6, 400 MHz)

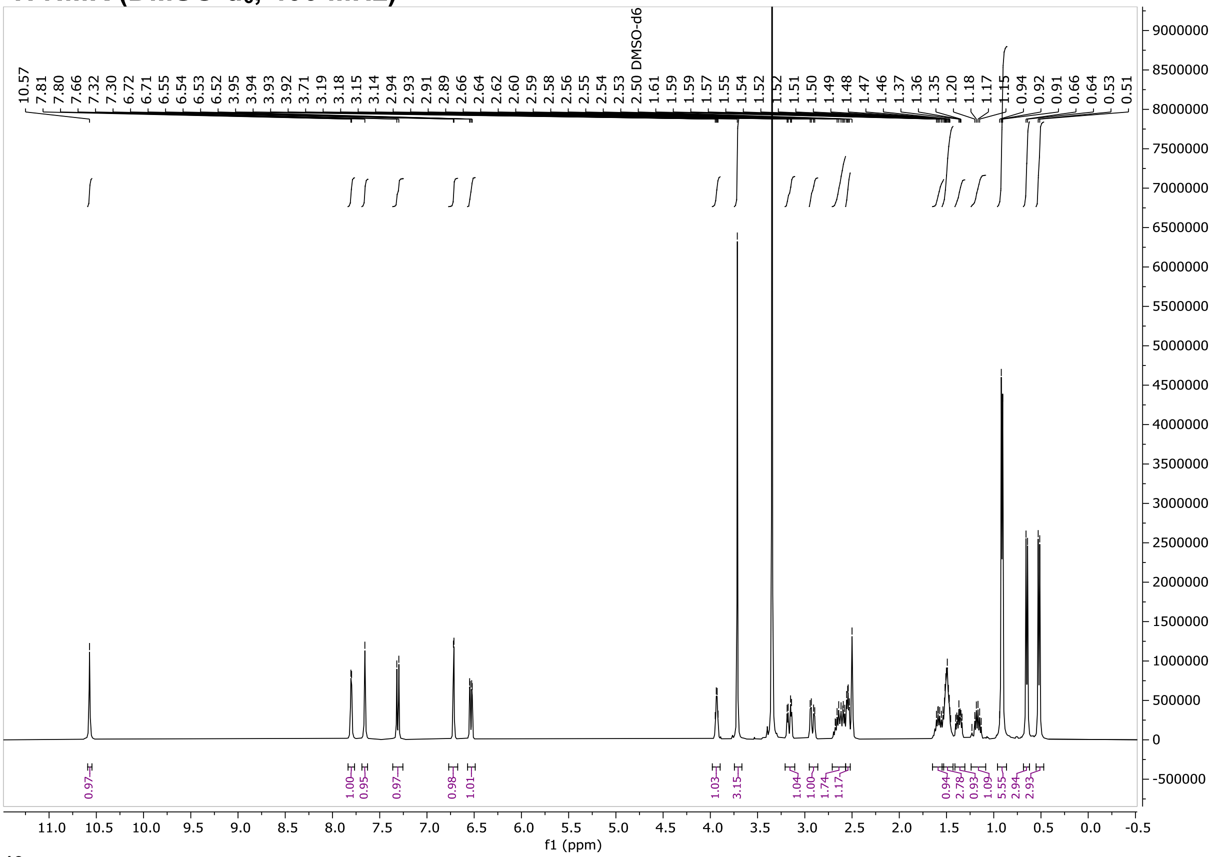

^{13}C NMR (DMSO-d_6, 101 MHz)

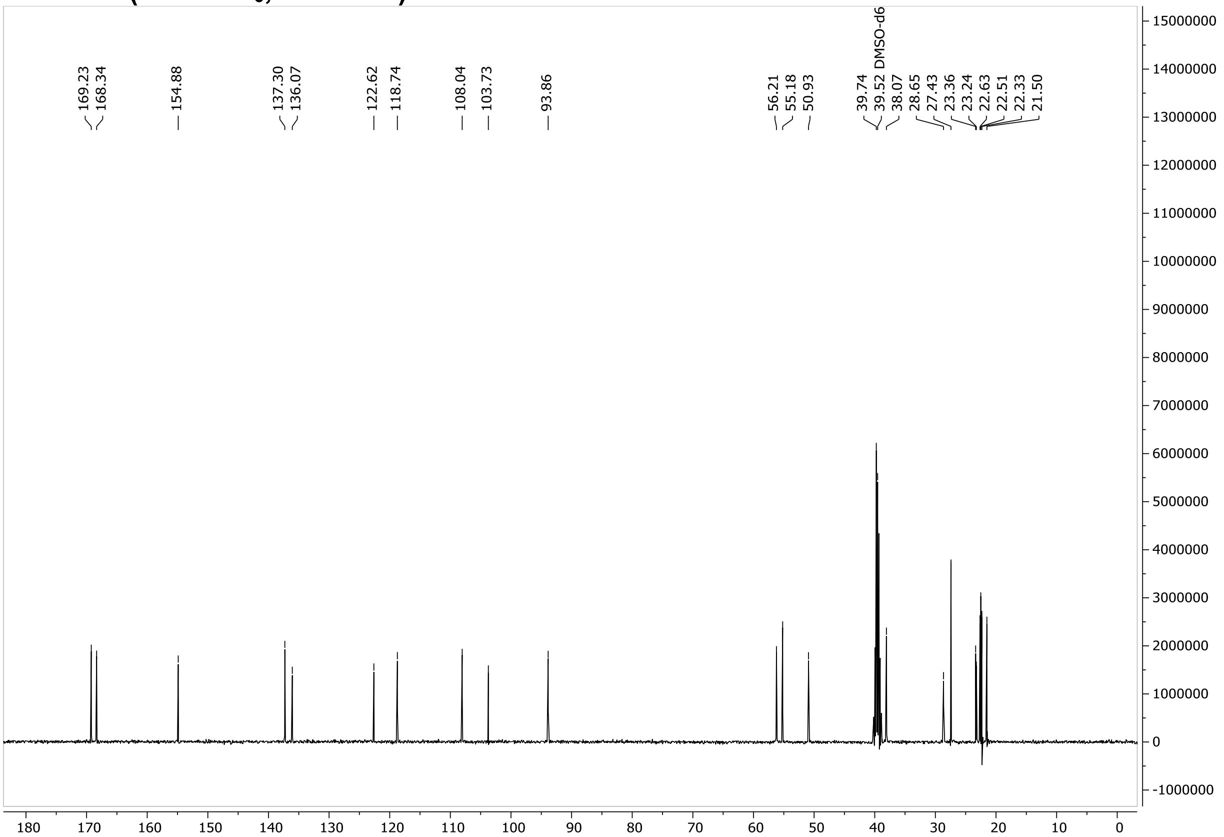

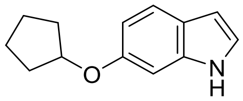

SI-10

6-(Cyclopentyloxy)-1H-indole (SI-10): To a solution of (commercially available) 6-hydroxyindole (**SI-9**) (300 mg, 2.25 mmol, 1.00 eq.) in acetone (7.51 mL, 0.30 M) were added Cs_2CO_3 (954 mg, 2.93 mmol, 1.30 eq.) and bromocyclopentane (0.36 mL, 3.38 mmol, 1.50 eq.) and the reaction mixture was stirred at rt overnight. After ca. 20 h additional bromide (0.18 mL) and Cs_2CO_3 (450 mg) where added and the mixture was left stirring for another 19 h. The reaction was quenched with water and the solution extracted with CH_2Cl_2. The combined organic layers were dried over MgSO4 and the solvent was removed under reduced pressure. The crude product was purified by FC (hexane/AcOEt 10:1) to give **SI-10** (356 mg, 79%) as a yellow solid.

R_f = 0.42 (hexane/AcOEt 4:1); **^{1}H-NMR** = (400 MHz, $CDCl_3$) δ 7.98 (s, 1H), 7.49 (d, *J* = 8.6 Hz, 1H), 7.11 – 7.05 (m, 1H), 6.86 (d, *J* = 2.0 Hz, 1H), 6.77 (dd, *J* = 8.6, 2.2 Hz, 1H), 6.46 – 6.49 (m, 1H), 4.79 (p, *J* = 4.4 Hz, 1H), 1.96 – 1.84 (m, 4H), 1.86 – 1.77 (m, 2H), 1.69 – 1.51 (m, 2H); **^{13}C-NMR** = (101 MHz, $CDCl_3$) δ 154.9, 136.7, 123.0, 122.1, 121.3, 111.6, 102.6, 97.0, 79.9, 33.0, 24.2; **IR** (film): $\tilde{\nu}$ **=** 3408, 2959, 2871, 1625, 1578, 1508, 1497, 1453, 1399, 1360, 1344, 1322, 1293, 1251, 1208, 1162, 1119, 1088, 1036, 987, 957, 898, 804, 757, 716, 609 cm^{-1}; **HRMS** (ESI): m/z calcd for $C_{13}H_{16}NO$ $[M+H]^+$: 202.1226, found: 202.1231.

^{1}H NMR (CDCl$_3$, 400 MHz)

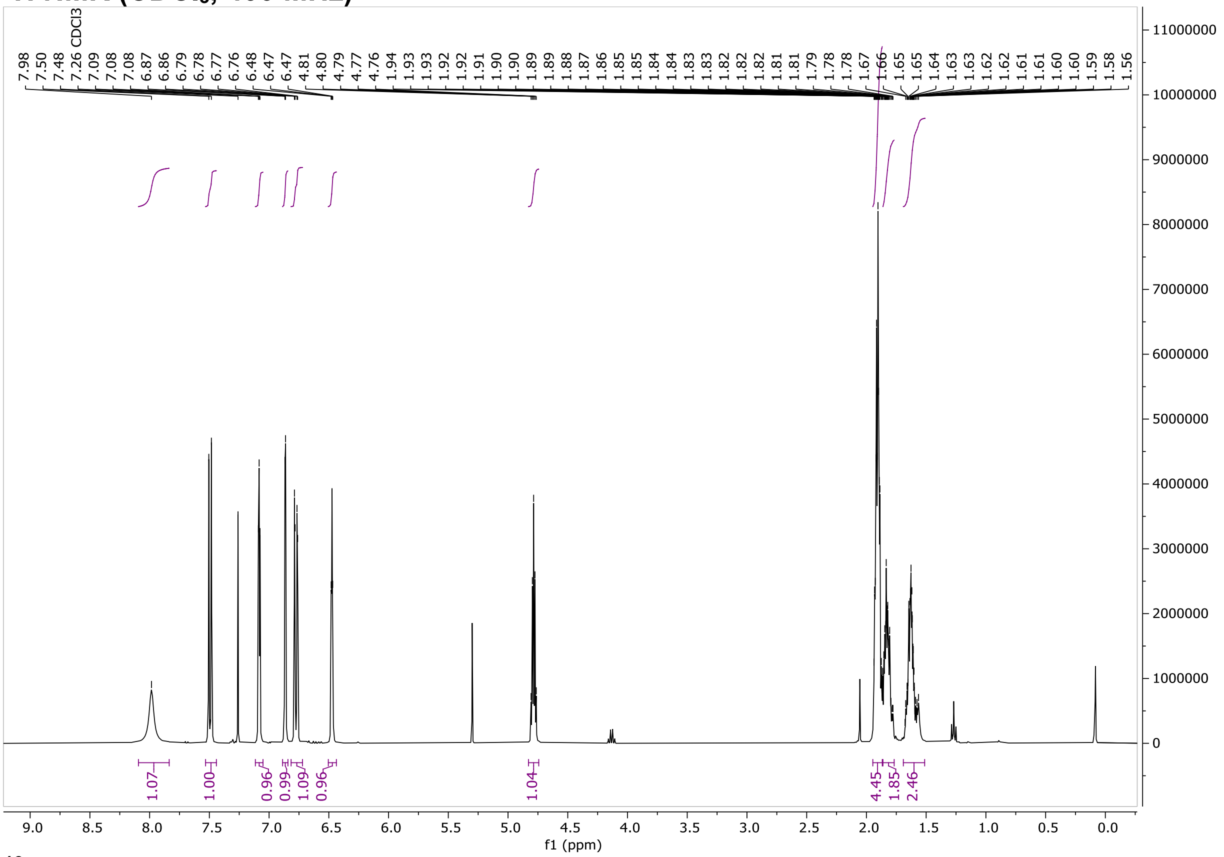

^{13}C NMR (CDCl$_3$, 101 MHz)

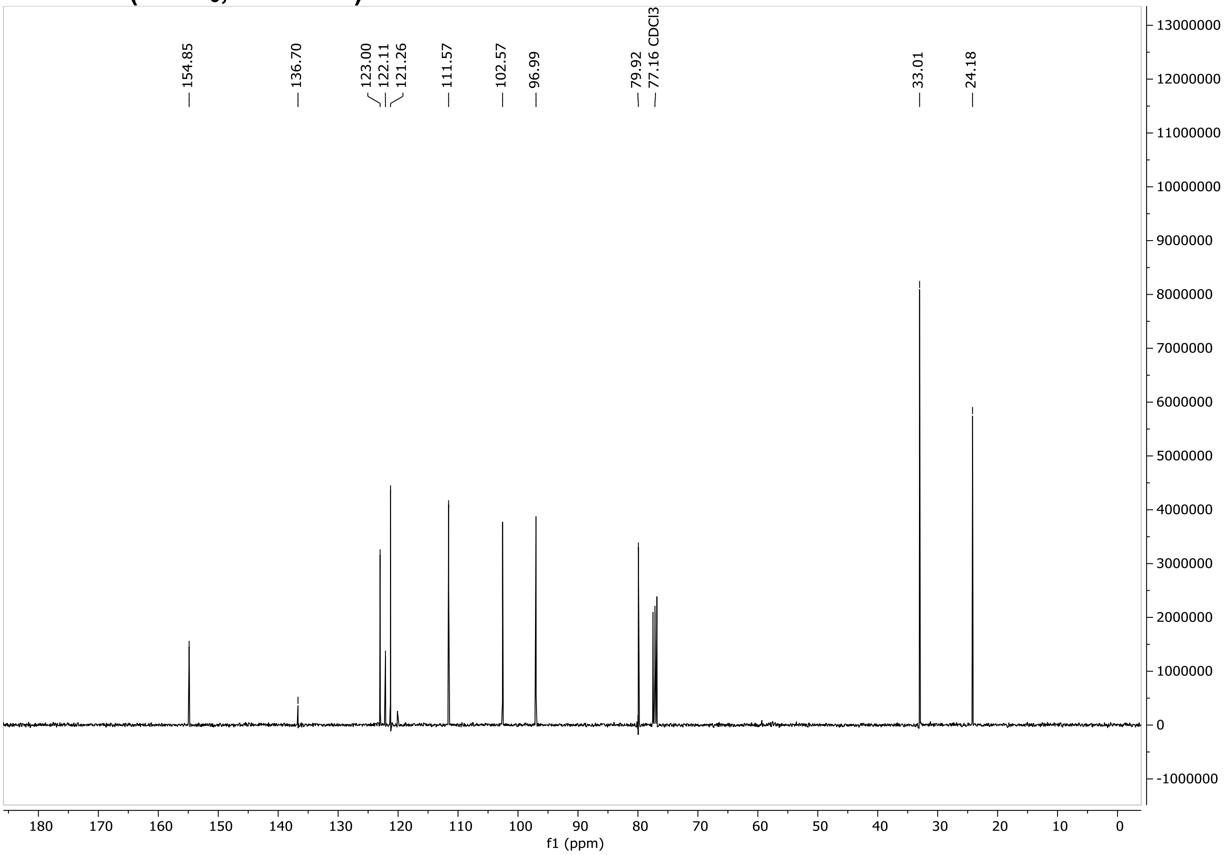

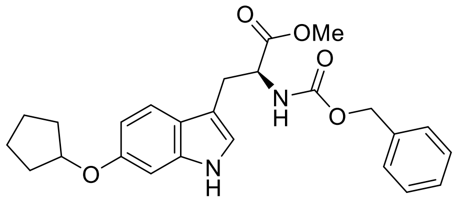

SI-11

Methyl (S)-2-(((benzyloxy)carbonyl)amino)-3-(6-(cyclopentyloxy)-1H-indol-3-yl)propanoate (SI-11): (*S*)-1-Benzyl 2-methyl aziridine-1,2-dicarboxylate (1**6**) (0.2 g, 0.85 mmol 1.00 eq.) and indole **SI-10** (0.342 g, 1.7 mmol, 2.00 eq) were dissolved in CH_2Cl_2 (3.0 ml) under argon and $Yb(OTf)_3$ (0.53 g, 0.85 mmol, 1.00 eq., dried overnight under vacuum at 150 °C) was added. The mixture was stirred at rt for 20 h and the reaction was quenched with water (3 ml). The solution was extracted with CH_2Cl_2 (3 x 3.5 ml), the combined organic layers were dried over $MgSO_4$, and the solvent was concentrated under reduced pressure. The residue was purified by FC (hexane/AcOEt 4:1 → 3:1 → 1:1) to yield tryptophan derivative **SI-11** as a sticky, yellow oil (181 mg, 49%).

R_f = 0.29 (hexane/AcOEt 2:1); **$[\alpha]_D^{20}$** = +25.99 (c = 0.5; $CHCl_3$); **^{1}H-NMR** = (400 MHz, $CDCl_3$) δ 7.91 (d, *J* = 12.0 Hz, 1H), 7.39 – 7.27 (m, 6H), 6.82 (d, *J* = 2.4 Hz, 1H), 6.80 (d, *J* = 2.2 Hz, 1H), 6.73 (dd, *J* = 8.7, 2.2 Hz, 1H), 5.32 (d, *J* = 8.1 Hz, 1H), 5.13 (d, *J* = 12.2 Hz, 1H), 5.08 (d, *J* = 12.4 Hz, 1H), 4.75 (p, *J* = 4.3 Hz, 1H), 4.69 (dt, *J* = 8.3, 5.5 Hz, 1H), 3.68 (s, 3H), 3.26 (d, *J* = 5.4 Hz, 2H), 1.93 – 1.85 (m, 4H), 1.84 – 1.76 (m, 2H), 1.68 – 1.55 (m, 2H); **^{13}C-NMR** = (101 MHz, $CDCl_3$) δ 172.6, 155.9, 155.1, 137.1, 136.5, 128.6, 128.3, 121.5, 119.3, 111.4, 110.0, 97.0, 79.9, 67.0, 54.6, 52.5, 33.0, 28.2, 24.2; **IR** (film): $\tilde{\nu}$ **=** 3356, 2954, 1705, 1627, 1550, 1498, 1454, 1438, 1353, 1302, 1261, 1210, 1164, 1128, 1084, 1056, 989, 908, 803, 731, 697, 484 cm^{-1}; **HRMS** (ESI): m/z calcd for $C_{25}H_{28}N_2NaO_5$ $[M+Na]^+$: 459.1890, found: 459.1889.

^{1}H NMR ($CDCl_3$, 400 MHz)

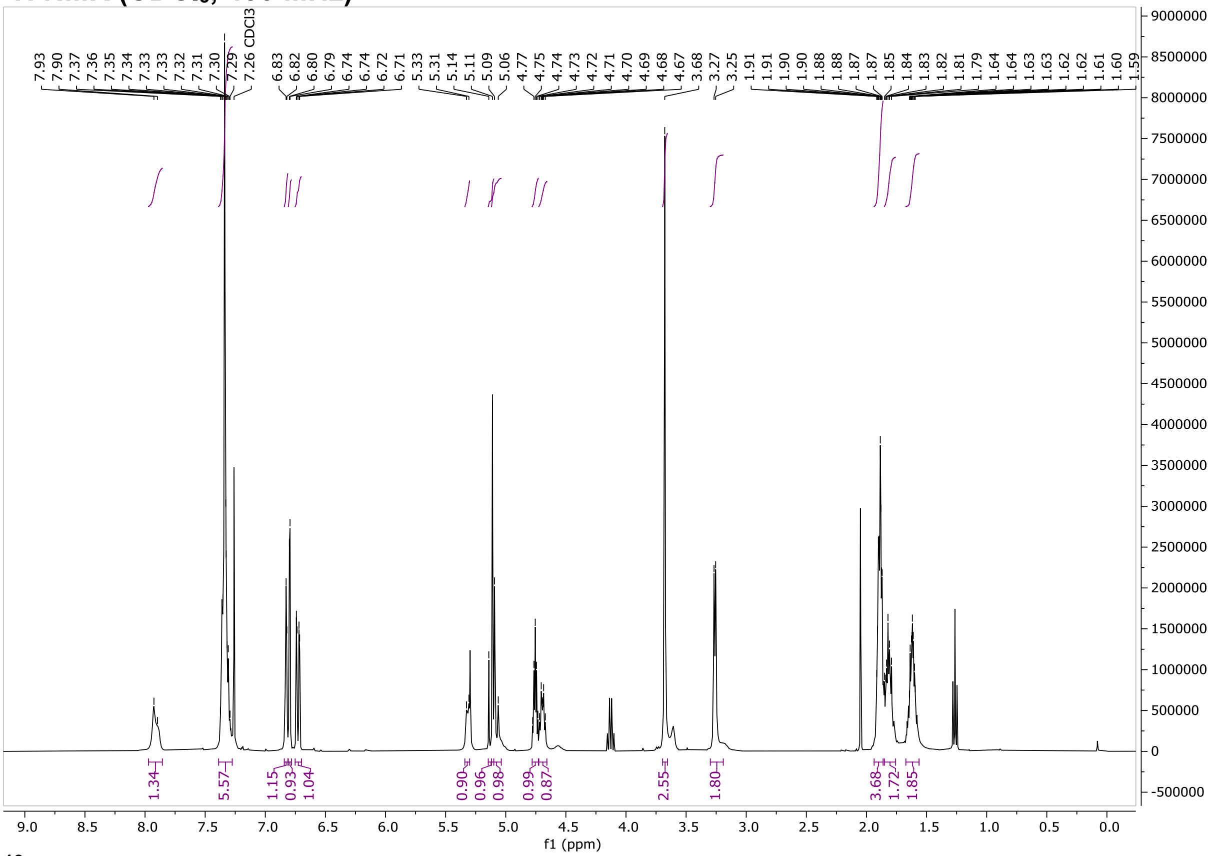

^{13}C NMR ($CDCl_3$, 101 MHz)

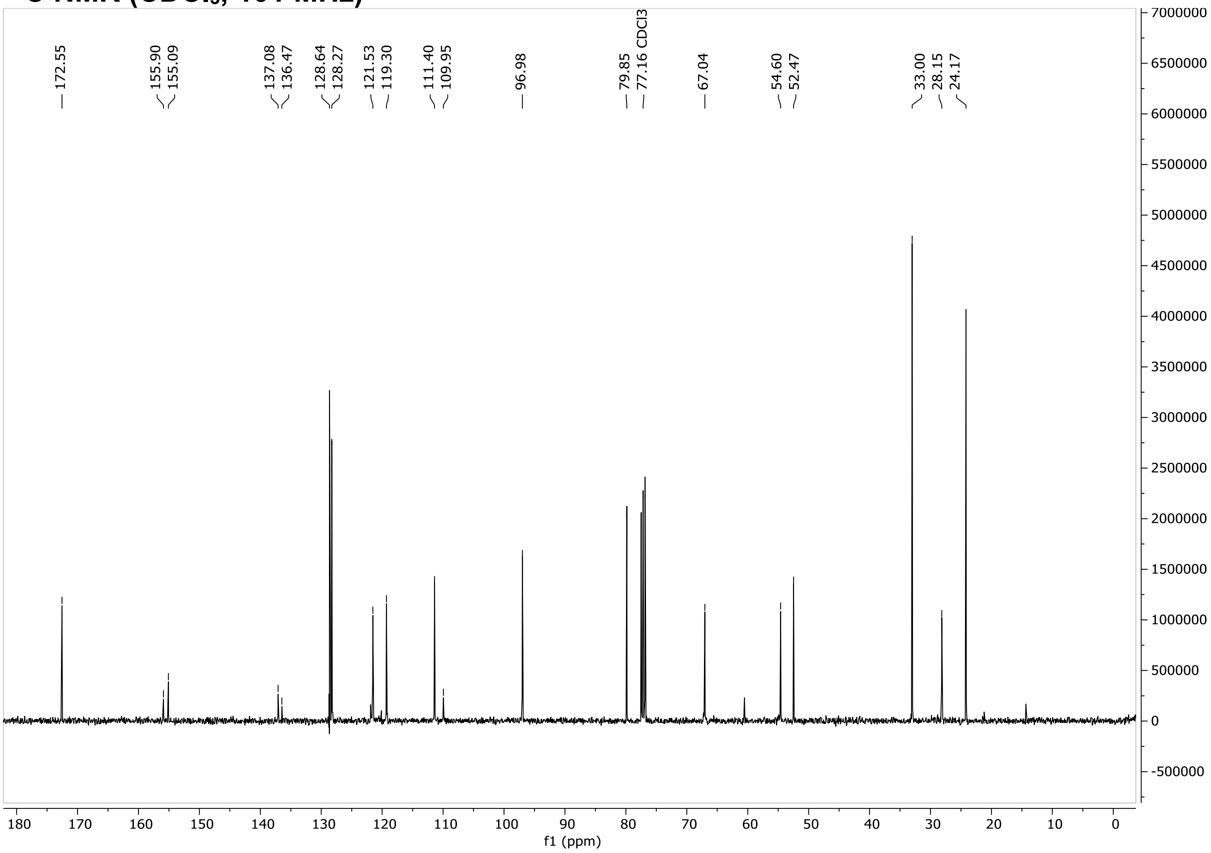

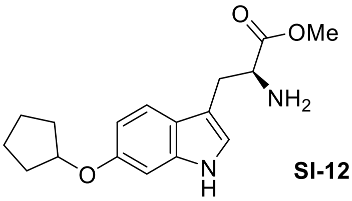

SI-12

Methyl (S)-2-amino-3-(6-(cyclopentyloxy)-1H-indol-3-yl)propanoate (SI-12): To a solution of **SI-11** (163 mg, 0.37 mmol, 1.00 eq.) in MeOH (7.46 ml) under argon was added Pd/C (10% w/w, 35 mg). The solution was degassed and the flask was flushed with hydrogen and the mixture was stirred at rt for 6 h. After complete conversion of the starting material the hydrogen was replaced by argon and the mixture was filtered over celite. The filtrate was concentrated under reduced pressure and the crude product was purified by FC (CH_2Cl_2/MeOH 20:1 to 10:1) to afford amino ester **SI-12** (99.1 mg, 88%) as a brown oil.

R_f = 0.29 (CH_2Cl_2/MeOH 20:1); **$[\alpha]_D^{20}$** = +6.82 (c = 0.44; $CHCl_3$); **^{1}H-NMR** = (400 MHz, $CDCl_3$) δ 7.98 (bs, 1H), 7.44 (d, *J* = 8.6 Hz, 1H), 6.94 (bs, 1H), 6.82 (d, *J* = 2.1 Hz, 1H), 6.76 (dd, *J* = 8.6, 2.2 Hz, 1H), 4.76 (p, *J* = 4.3 Hz, 1H), 3.80 (m, 1H), 3.71 (s, 3H), 3.24 (dd, *J* = 14.4, 4.8 Hz, 1H), 3.01 (dd, *J* = 14.4, 7.7 Hz, 1H), 1.96 – 1.84 (m, 4H), 1.85 – 1.76 (m, 2H), 1.68 – 1.55 (m, 2H); **^{13}C-NMR** = (101 MHz, $CDCl_3$) δ 175.8, 155.1, 137.2, 121.8, 121.8, 119.4, 111.3, 111.2, 97.1, 79.9, 55.0, 52.2, 33.0, 30.9, 24.2; **IR** (film): $\tilde{\nu}$ **=** 3371, 2953, 2871, 1735, 1627, 1578, 1551, 1498, 1455, 1438, 1360, 1302, 1261, 1200, 1167, 1099, 990, 803, 731 cm^{-1}; **HRMS** (ESI): m/z calcd for $C_{17}H_{22}N_2NaO_3$ $[M+Na]^+$: 325.1523, found: 325.1523.

^{1}H NMR ($CDCl_3$, 400 MHz)

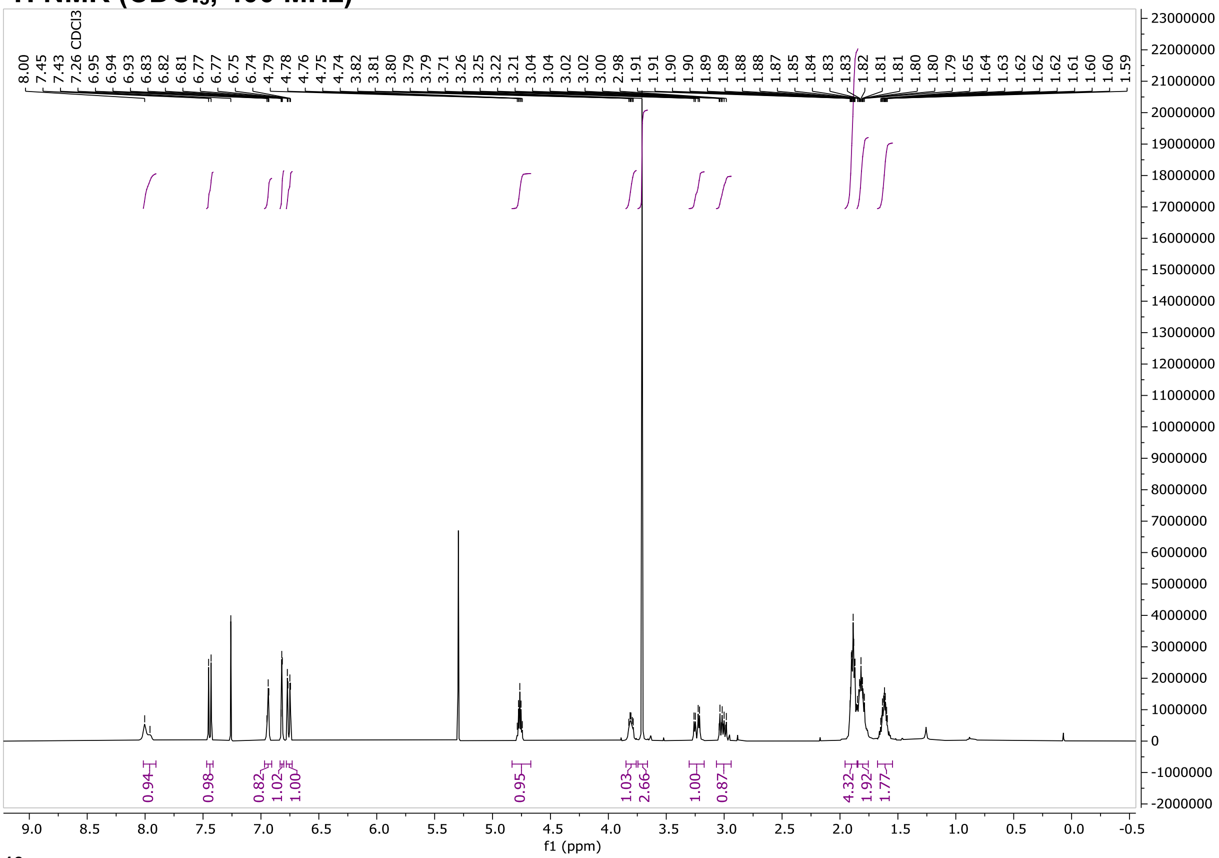

^{13}C NMR ($CDCl_3$, 101 MHz)

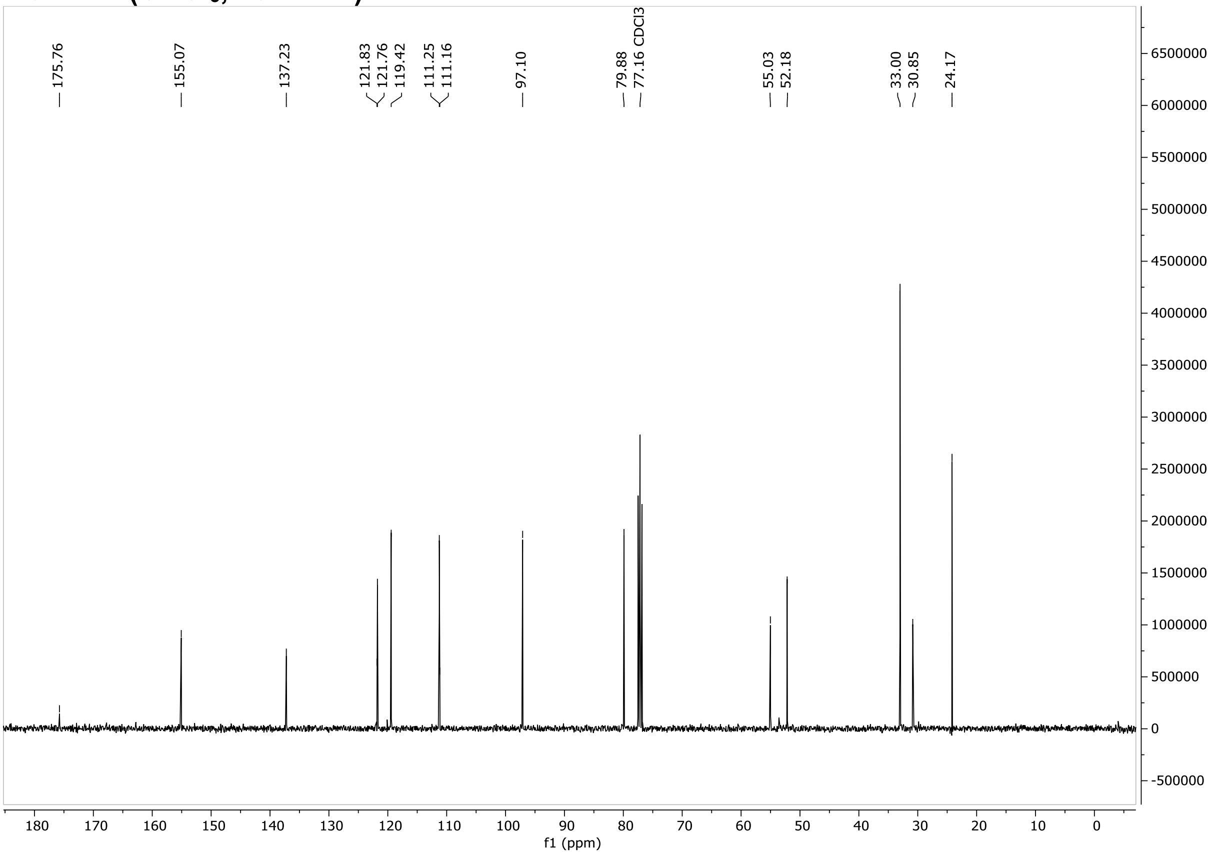

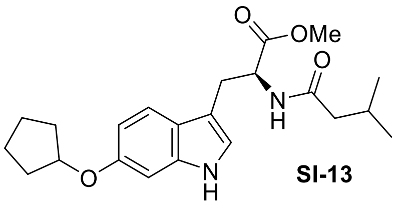

SI-13

Methyl (S)-3-(6-(cyclopentyloxy)-1H-indol-3-yl)-2-(3-methylbutanamido)propanoate (SI-13): To a solution of amino ester **SI-12** (85 mg, 0.28 mmol, 1.0 eq.) in THF (1.5 ml) were added Et_3N (51 µl, 0.37 mmol, 1.3 eq.) and isovaleryl chloride (41 µl, 0.34 mmol, 1.2 eq.) at rt and the reaction mixture was stirred for 10 min. The reaction was quenched with MeOH and the mixture filtered through a pad of Celite and rinsed with Et_2O. The filtrate was washed with brine, dried over $MgSO_4$ and the solvent was removed under reduced pressure at ambient temperature. The crude product was purified by FC (hexane/AcOEt 2:1) to obtain amide **SI-13** (75 mg, 69%) as a white foam.

R_f = 0.15 (hexane/AcOEt 2:1); **$[\alpha]_D^{20}$** = +37.49 (c = 0.4; $CHCl_3$); **^{1}H-NMR** = (400 MHz, $CDCl_3$) δ 7.90 (s, 1H), 7.37 (d, *J* = 8.6 Hz, 1H), 6.85 (d, *J* = 2.3 Hz, 1H), 6.82 (d, *J* = 2.1 Hz, 1H), 6.75 (dd, *J* = 8.6, 2.1 Hz, 1H), 5.91 (d, *J* = 8.0 Hz, 1H), 4.95 (dt, *J* = 8.0, 5.4 Hz, 1H), 4.77 (p, *J* = 4.8 Hz, 1H), 3.69 (s, 3H), 3.26 (d, *J* = 5.5 Hz, 2H), 2.13 – 2.05 (m, 1H), 2.04 – 1.97 (m, 2H), 1.93 – 1.86 (m, 4H), 1.85 – 1.77 (m, 1H), 1.68 – 1.56 (m, 3H), 0.92 (d, *J* = 1.8 Hz, 3H), 0.90 (d, *J* = 1.9 Hz, 3H); **^{13}C-NMR** = (101 MHz, $CDCl_3$) δ 172.7, 172.2, 155.1, 137.1, 122.1, 121.3, 119.3, 111.5, 110.3, 97.0, 79.9, 52.8, 52.4, 46.0, 33.0, 27.9, 26.2, 24.2, 22.6, 22.5; **IR** (film): $\tilde{\nu}$ **=** 3301, 2956, 2871, 1741, 1650, 1629, 1500, 1439, 1369, 1303, 1260, 1208, 1169, 992, 913, 800, 757, 744, 735 cm^{-1}; **HRMS** (ESI): m/z calcd for $C_{22}H_{30}N_2NaO_4$ $[M+Na]^+$: 409.2098, found: 409.2096.

^{1}H NMR ($CDCl_3$, 400 MHz)

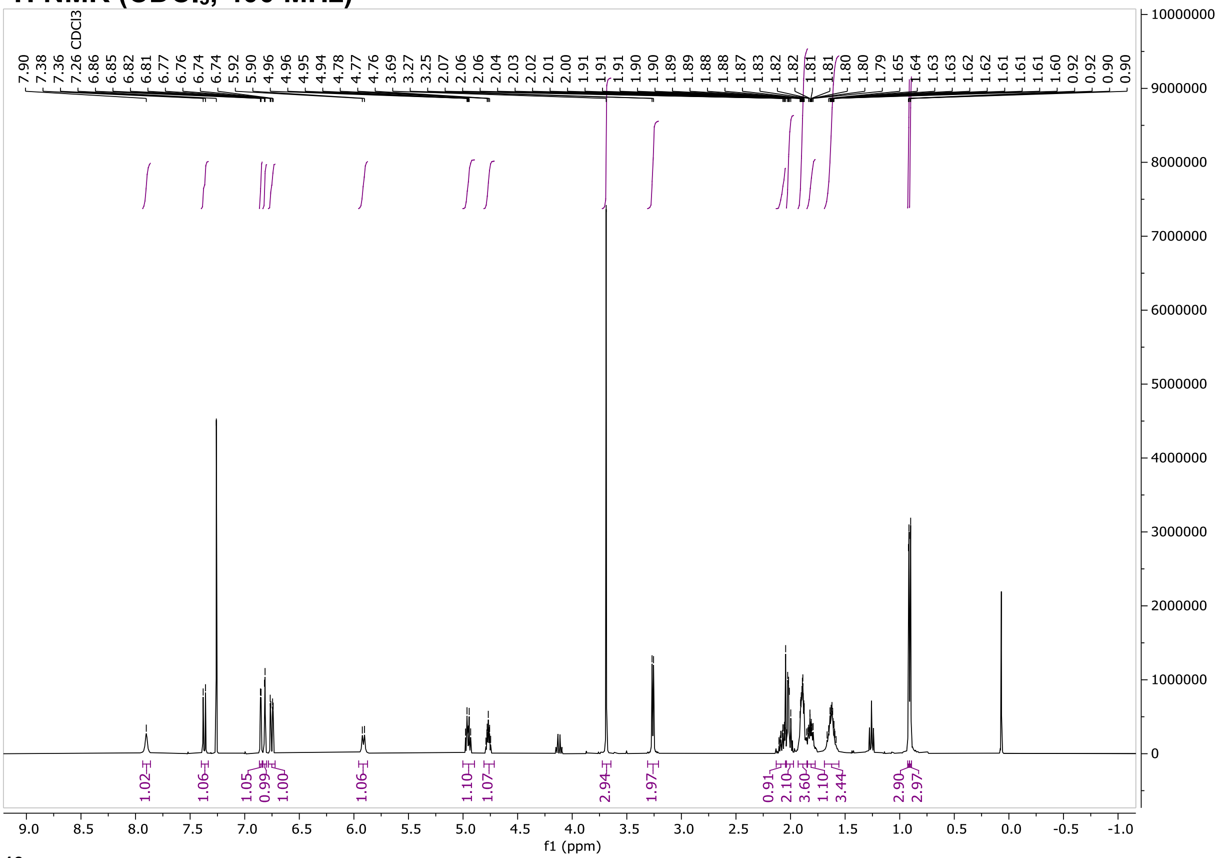

^{13}C NMR ($CDCl_3$, 101 MHz)

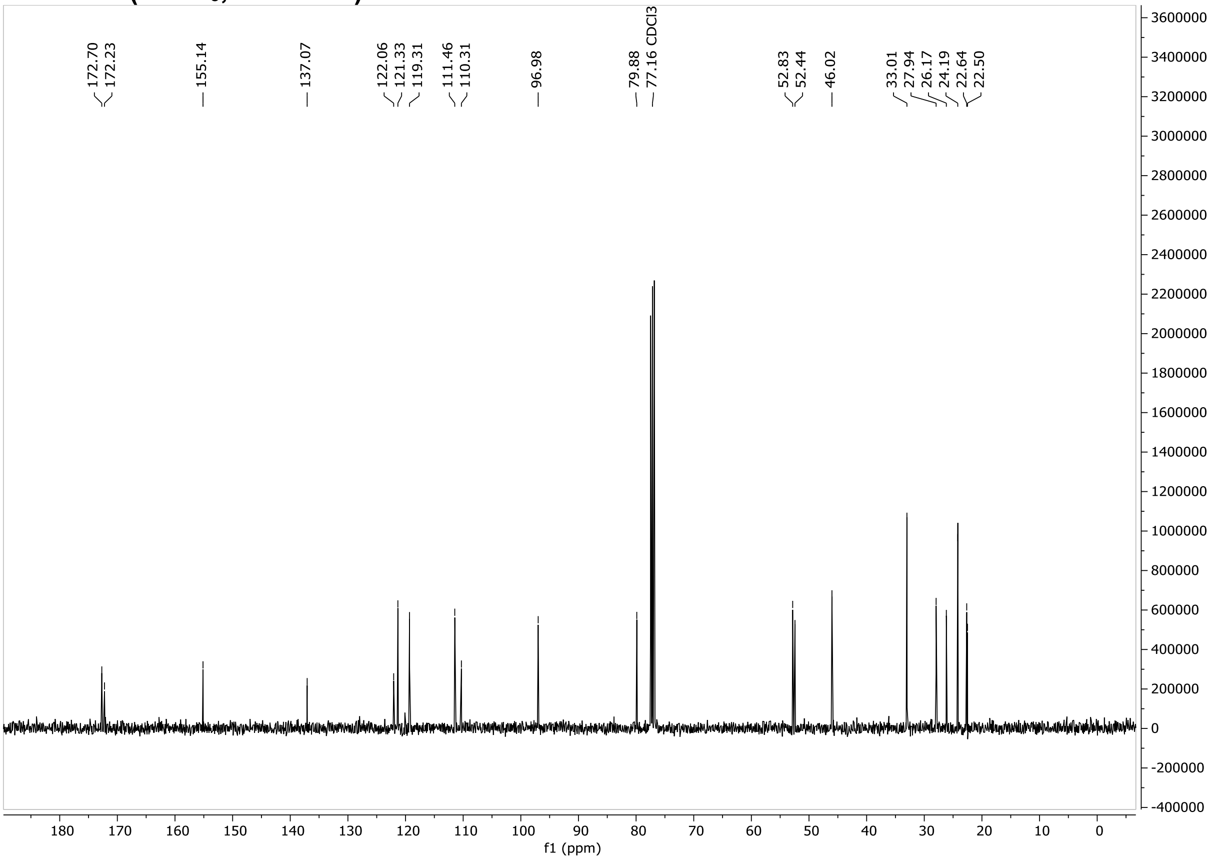

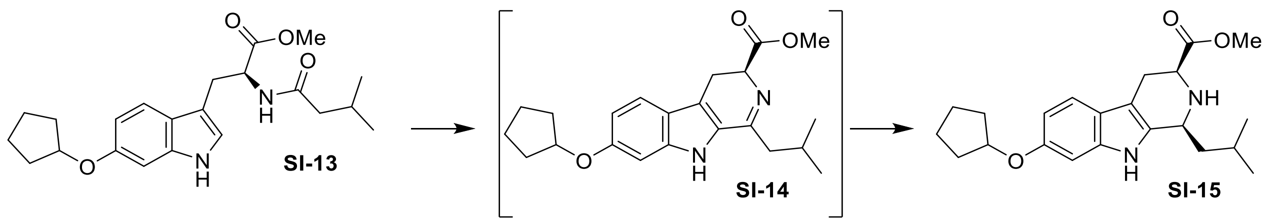

Methyl (1S,3S)-7-(cyclopentyloxy)-1-isobutyl-2,3,4,9-tetrahydro-1H-pyrido[3,4-b]indole-3-carboxylate (SI-15): To a solution of amide **SI-13** (70 mg, 0.18 mmol, 1.0 eq.) in benzene (2.26 ml) was added $POCl_3$ (0.05 ml, 0.54 mmol, 3.0 eq.) at 5 °C. Then the reaction mixture was stirred at reflux for 3 h. The solvent was removed under reduced pressure and the crude product, which was assumed to be imine **S-14**, was directly used for the next step without purification.

Imine **SI-14** (67 mg, 0.17 mmol, 1.0 eq.) was dissolved in MeOH (5.43 ml) followed by the addition of $NaBH_4$ (65 mg, 1.73 mmol, 10.0 eq.) at 0 °C. The reaction mixture was stirred at 0 °C for 5 min. The reaction was quenched with aq. sat. $NaHCO_3$ followed by the extraction with $CHCl_3$/MeOH 5% (6 x). The combined organic layers were dried over $MgSO_4$ and the solvent was removed under reduced pressure. The residue was purified by FC (hexane/AcOEt 4:1) to yield tetrahydro-β-carboline **SI-15** (33 mg, 52%) as a sticky, yellow solid.

R_f = 0.34 (hexane/AcOEt 2:1); **$[\alpha]_D^{20}$** = –56.99 (c = 1.0; $CHCl_3$); **^{1}H-NMR** = (400 MHz, $CDCl_3$) δ 7.69 (s, 1H), 7.32 (d, *J* = 8.5 Hz, 1H), 6.81 (d, *J* = 2.2 Hz, 1H), 6.74 (dd, *J* = 8.6, 2.2 Hz, 1H), 4.75 (p, *J* = 4.3 Hz, 1H), 4.19 (ddd, *J* = 9.1, 4.5, 2.3 Hz, 1H), 3.81 (s, 3H), 3.77 (dd, *J* = 11.0, 4.2 Hz, 1H,), 3.07 (ddd, *J* = 15.1, 4.3, 2.0 Hz, 1H), 2.78 (ddd, *J* = 15.1, 11.1, 2.7 Hz, 1H), 2.05 – 1.96 (m, 1H), 1.91 – 1.83 (m, 4H), 1.86 – 1.73 (m, 2H), 1.73 – 1.53 (m, 4H), 1.03 (d, *J* = 6.5 Hz, 3H), 0.99 (d, *J* = 6.6 Hz, 3H); **^{13}C-NMR** = (101 MHz, $CDCl_3$) δ 174.0, 154.6, 136.8, 134.8, 121.7, 118.5, 110.8, 107.7, 97.5, 80.0, 56.6, 52.3, 50.7, 44.7, 33.0, 26.2, 24.5, 24.1, 24.0, 21.9; **IR** (film): $\tilde{\nu}$ **=** 3382, 2954, 2869, 1737, 1629, 1572, 1498, 1470, 1437, 1345, 1264, 1217, 1157, 992, 807, 770.423 cm^{-1}; **HRMS** (ESI): m/z calcd for $C_{22}H_{31}N_2O_3$ $[M+H]^+$: 371.2329, found: 371.2328.

^{1}H NMR ($CDCl_3$, 400 MHz)

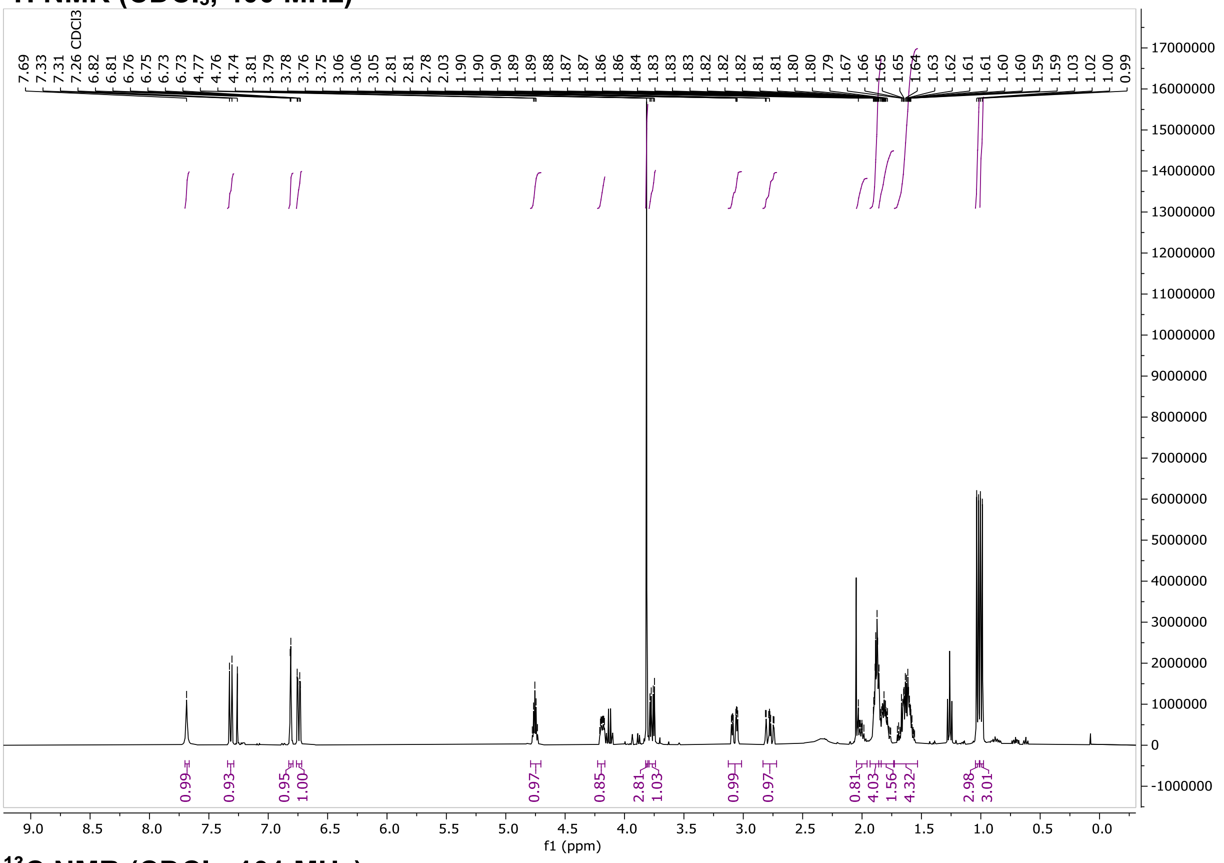

^{13}C NMR ($CDCl_3$, 101 MHz)

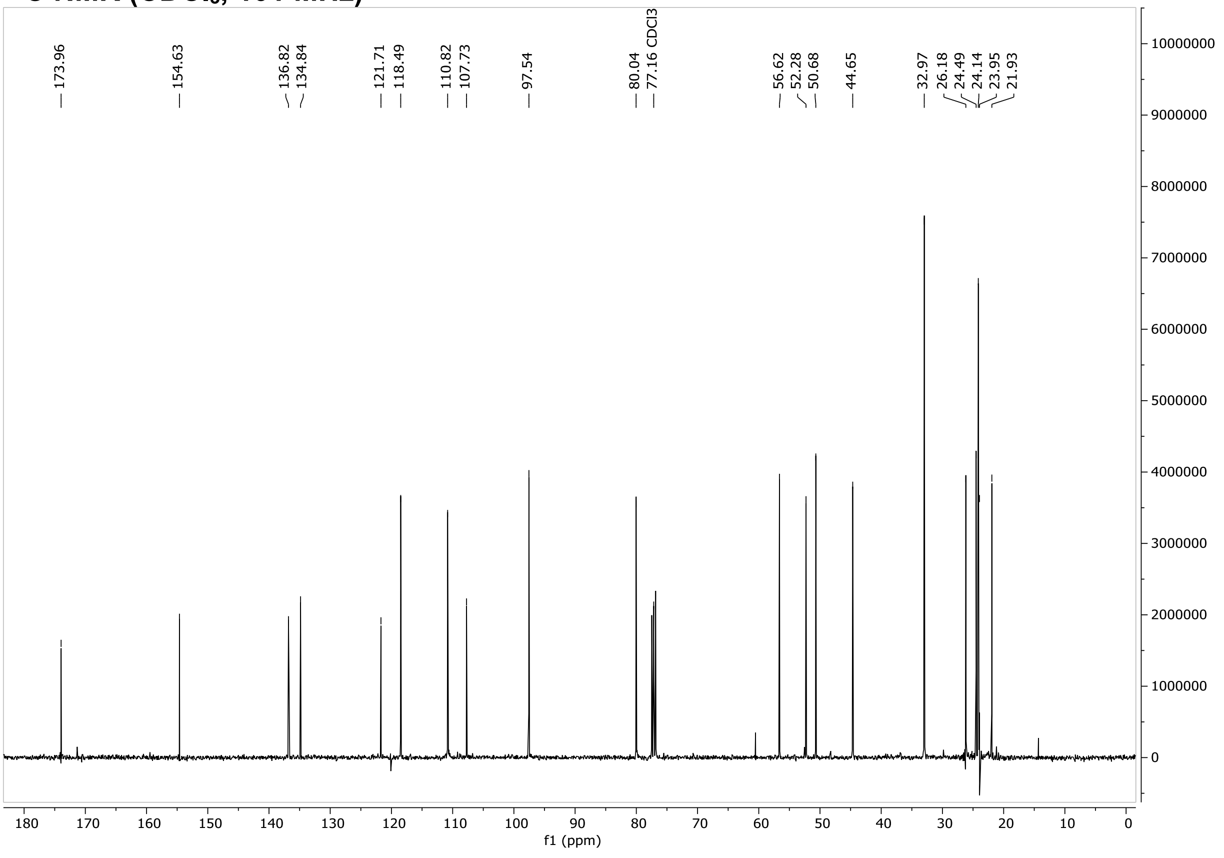

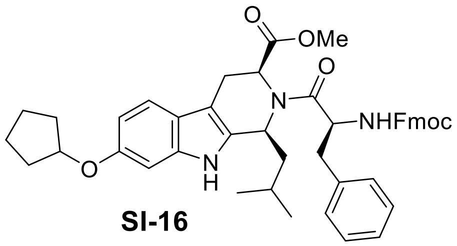

SI-16

Methyl (1S,3S)-2-((((9H-fluoren-9-yl)methoxy)carbonyl)-L-phenylalanyl)-7-(cyclopentyloxy)-1-isobutyl-2,3,4,9-tetrahydro-1H-pyrido[3,4-b]indole-3-carboxylate (SI-16): A solution of Nα-Fmoc-Phe-OH (20 mg, 52 µmol, 1.00 eq.) and tetrahydro-β-carboline **SI-15** (21 mg, 57 µmol, 1.10 eq.) in CH_2Cl_2 (0.8 mL) was stirred for 10 min at rt. Then DMTMM (31 mg, 0.11 mmol, 2.20 eq.) was added and the solution was stirred at rt until full conversion of the amino acid was reached (ca. 2 d). The reaction was quenched with water and the mixture was extracted with CH_2Cl_2 (3 x). The combined organic layers were washed with aq. sat. $NaHCO_3$, water, 1M HCl, water and brine, dried over $MgSO_4$ and the solvent was removed under reduced pressure. The crude material was purified by FC (hexane/AcOEt 4:1) to yield amide **SI-16** was as a yellow solid (54%). This material was submitted to the next step without characterization.

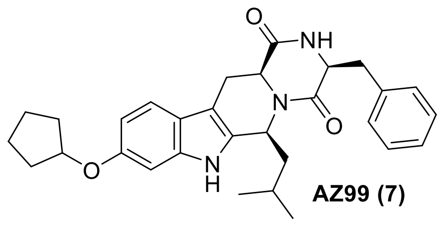

AZ99 (7)

(3S,6S,12aS)-3-Benzyl-9-(cyclopentyloxy)-6-isobutyl-2,3,6,7,12,12a-hexahydropyrazino[1',2':1,6] pyrido[3,4-b]indole-1,4-dione (AZ99 (7)):To a solution of amide **SI-16** (30 mg, 0.04 mmol, 1.00 eq.) in dry CH_2Cl_2 (0.34 mL, 0.11 M) was added piperidine under argon (0.06 mL, 0.65 mmol, 15.9 eq.) and the reaction mixture was stirred at rt for 1 h. The solution was concentrated *in vacuo* and the residue was purified by FC (hexane/AcOEt 1:1) to yield **AZ99 (7)** as a white solid (12 mg, 61%).

R_f = 0.35 (hexane/AcOEt 1:1); **$[\alpha]_D^{20}$** = –90.72 (c = 0.54; MeOH); **^{1}H-NMR** = (400 MHz, $CDCl_3$) δ 7.91 (s, 1H), 7.44 – 7.23 (m, 6H), 6.87 (d, *J* = 2.2 Hz, 1H), 6.80 (dd, *J* = 8.6, 2.2 Hz, 1H), 5.78 (s, 1H,), 5.49 (dd, *J* = 9.3, 4.1 Hz, 1H), 4.77 (p, *J* = 4.4 Hz, 1H), 4.20 (ddd, *J* = 10.6, 3.9, 1.2 Hz, 1H), 4.03 (dd, *J* = 11.6, 4.7 Hz, 1H), 3.70 (dd, *J* = 14.3, 3.8 Hz, 1H), 3.51 (dd, *J* = 15.7, 4.8 Hz, 1H), 2.98 (dd, *J* = 15.7, 11.6 Hz, 1H), 2.88 (dd, *J* = 14.4, 10.7 Hz, 1H), 1.93 – 1.85 (m, 4H), 1.85 – 1.75 (m, 2H), 1.67 – 1.51 (m, 5H), 1.08 (d, *J* = 6.4 Hz, 3H), 0.85 (d, *J* = 6.3 Hz, 3H); **^{13}C-NMR** = (101 MHz, $CDCl_3$) δ 169.4, 168.3, 155.0, 136.7, 135.9, 133.0, 129.5, 129.3, 127.8, 120.6, 118.8, 111.5, 106.9, 97.6, 80.0, 56.2, 55.2, 51.4, 46.1, 37.4, 33.0, 25.0, 24.2, 24.0, 22.2, 21.8; **IR** (film): $\tilde{\nu}$ **=** 2957, 1683, 1628, 1055, 1033, 1014**;** **HRMS** (ESI): m/z calcd for $C_{30}H_{35}N_3NaO_3$ $[M+Na]^+$: 508.2571, found: 508.2566.

^{1}H NMR ($CDCl_3$, 400 MHz)

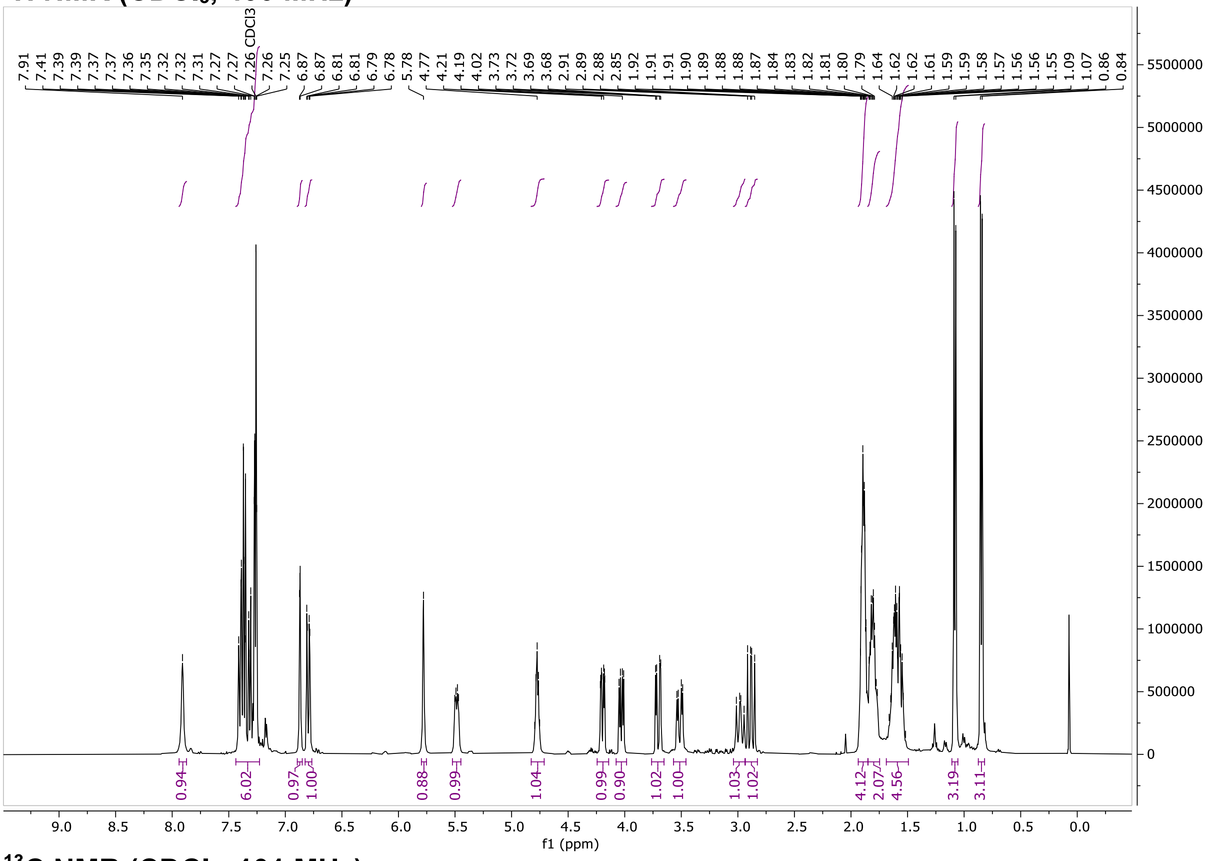

^{13}C NMR ($CDCl_3$, 101 MHz)

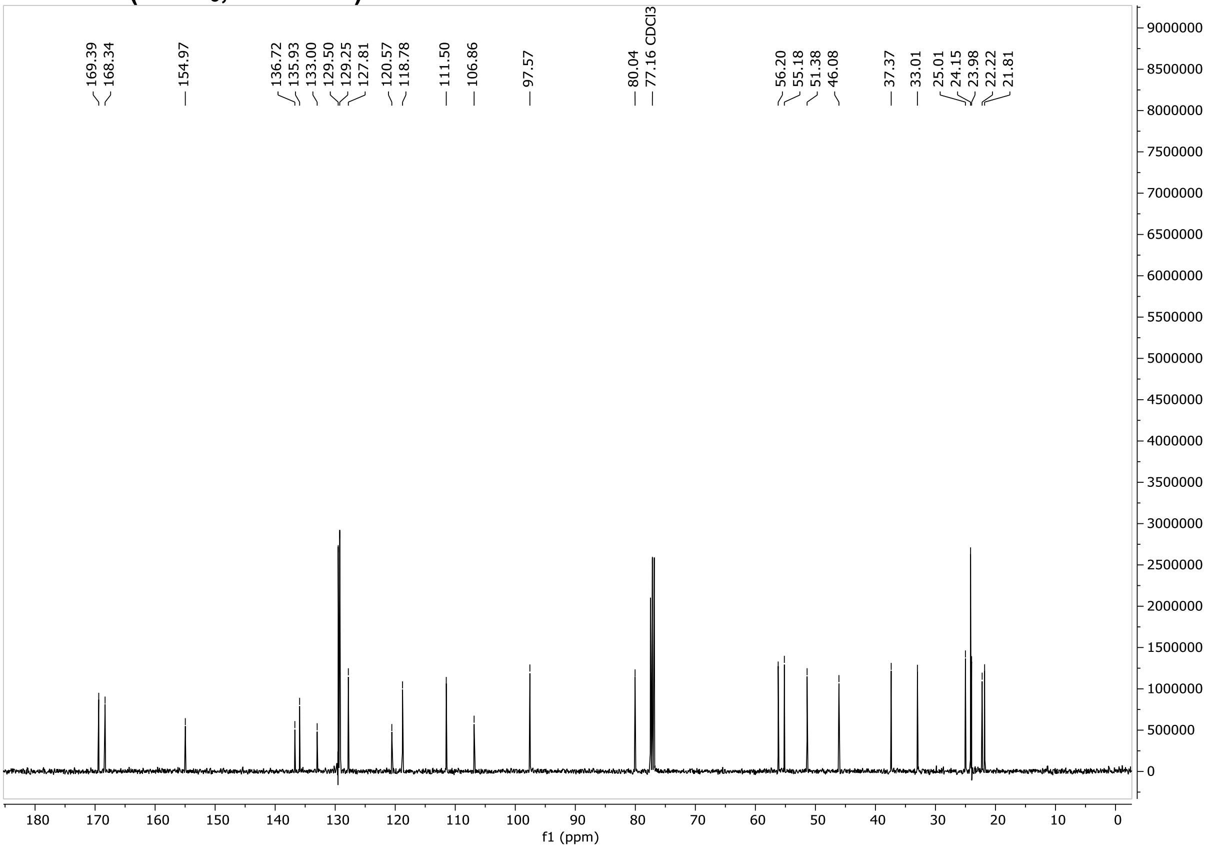

Supplementary table

Data collection and processing	ABCG2-Fab-MZ82 (Final)	ABCG2-Fab-Ko143 (Final)	ABCG2-Fab-AZ99 (Final)	ABCG2-Fab-MZ29 reprocessed
**Energy filters / detector**	BioQuantum / K3	BioQuantum / K3	BioQuantum / K3	
**Energy window (eV)**	20	20	20	
**Magnification (nominal)**	130,000 x	130,000 x	130,000 x	
**Voltage (kV)**	300	300	300	
**Electron exposure per movie ($e^-/Å^2$)**	42	58	58	
**Exposure Time(s)**	2.5/1.25	1.01	1.01	Published
**Exposure rate (e/pix/sec)**	8/15	25	25	
**Electron exposure per frame ($e^-/Å^2$/frame)**	1	1.45	1.45	
**Defocus range (μm)**	-0.6- -2.5	-0.2- -2.5	-0.2- -2.5	
**Pixel size (Å)**	0.66	0.66	0.66	0.84
**symmetry**	C1	C2	C2	C2
**Initial particle images (No.)**	4,118,521	708,440	848,463	1,042,226
**Final particle images (No.)**	601,814	174,256	201,796	557,549
**Map resolution (Å)**	2.39	3.00	3.00	2.56
**FSC threshold**	0.143	0.143	0.143	0.143
**Map resolution range (Å)**	1.7-9.0	2.6-9.0	2.8-9.0	2.5-20
	**Model refinement**			
**Model resolution(Å)**	2.6	3.0	3.1	2.6
**FSC threshold**	0.5	0.5	0.5	0.5
**Map sharpening B factor ($Å^2$)**	-55	-50	-80	–44
**Model composition**				
**Non-hydrogen atoms**	12,653	12,747	12,542	12774
**protein residues**	1,582	1,582	1,581	1,582
**Ligands**	MZ82:2<br>NAG:4<br>CLR:6	Ko143:2<br>NAG:4<br>CLR:10	AZ99:2<br>NAG:4<br>CLR:4	NAG: 4<br>CLR: 10<br>MZ29: 2
**B factors ($Å^2$)**				
**protein**	24.8/145.7/69.0	20.9/91.5/48.2	22.7/94.6/48.8	23.5/107.9/56.5
**ligand**	33.7/67.3/47.0	28.469.7/40.5	32.3/51.5/40.5	39.7/66.2/51.1
**R.m.s.d deviations**				
**Bond lengths (Å)**	0.007	0.008	0.010	0.009
**bond angles (°)**	0.674	0.631	0.629	0.978
**Validation**				
**MolProbity score**	1.55	1.43	1.48	1.84

**Clash score**	3.79	3.60	3.22	10.83
**Poor rotamers (%)**	1.19	0.89	1.04	1.04
**Ramachandran plot**				
**Favored (%)**	95.25	95.89	94.93	96.02
**Allowed (%)**	4.75	4.11	5.07	3.85
**Disallowed (%)**	0	0	0	0.13
**PDB**	N/A	N/A	N/A	N/A
**EMDB**	N/A	N/A	N/A	EMPIAR-10374

Table S1. Cryo-EM data collection, refinement, and validation statistics.

References

1. Taylor, N. M. I.; Manolaridis, I.; Jackson, S. M.; Kowal, J.; Stahlberg, H.; Locher, K. P., Structure of the human multidrug transporter ABCG2. *Nature* **2017,** *546* (7659), 504-509.
2. Yu, Q.; Ni, D.; Kowal, J.; Manolaridis, I.; Jackson, S. M.; Stahlberg, H.; Locher, K. P., Structures of ABCG2 under turnover conditions reveal a key step in the drug transport mechanism. *Nat Commun* **2021,** *12* (1), 4376.
3. Ritchie, T. K.; Grinkova, Y. V.; Bayburt, T. H.; Denisov, I. G.; Zolnerciks, J. K.; Atkins, W. M.; Sligar, S. G., Chapter 11 - Reconstitution of membrane proteins in phospholipid bilayer nanodiscs. *Methods Enzymol.* **2009,** *464*, 211-31.
4. Geertsma, E. R.; Mahmood, N. A. B. N.; Schuurman-Wolters, G. K.; Poolman, B., Membrane reconstitution of ABC transporters and assays of translocator function. *Nat Protoc* **2008,** *3* (2), 256-266.
5. Chifflet, S.; Torriglia, A.; Chiesa, R.; Tolosa, S., A method for the determination of inorganic phosphate in the presence of labile organic phosphate and high concentrations of protein: Application to lens ATPases. *Analytical Biochemistry* **1988,** *168* (1), 1-4.
6. Li, X. M.; Mooney, P.; Zheng, S.; Booth, C. R.; Braunfeld, M. B.; Gubbens, S.; Agard, D. A.; Cheng, Y. F., Electron counting and beam-induced motion correction enable near-atomic-resolution single-particle cryo-EM. *Nat Methods* **2013,** *10* (6), 584-+.
7. Zhang, K., Gctf: Real-time CTF determination and correction. *J. Struct. Biol.* **2016,** *193* (1), 1-12.
8. Jackson, S. M.; Manolaridis, I.; Kowal, J.; Zechner, M.; Taylor, N. M. I.; Bause, M.; Bauer, S.; Bartholomaeus, R.; Bernhardt, G.; Koenig, B.; Buschauer, A.; Stahlberg, H.; Altmann, K. H.; Locher, K. P., Structural basis of small-molecule inhibition of human multidrug transporter ABCG2. *Nat Struct Mol Biol* **2018,** *25* (4), 333-+.
9. Emsley, P.; Lohkamp, B.; Scott, W. G.; Cowtan, K., Features and development of Coot. *Acta Crystallogr D* **2010,** *66*, 486-501.
10. Moriarty, N. W.; Grosse-Kunstleve, R. W.; Adams, P. D., electronic Ligand Builder and Optimization Workbench (eLBOW): a tool for ligand coordinate and restraint generation. *Acta Crystallogr D* **2009,** *65* (Pt 10), 1074-80.
11. Afonine, P. V.; Poon, B. K.; Read, R. J.; Sobolev, O. V.; Terwilliger, T. C.; Urzhumtsev, A.; Adams, P. D., Real-space refinement in PHENIX for cryo-EM and crystallography. *Acta Crystallogr. D Struct. Biol.* **2018,** *74* (Pt 6), 531-544.

12. Chen, V. B.; Arendall, W. B., 3rd; Headd, J. J.; Keedy, D. A.; Immormino, R. M.; Kapral, G. J.; Murray, L. W.; Richardson, J. S.; Richardson, D. C., MolProbity: all-atom structure validation for macromolecular crystallography. *Acta Crystallogr D* **2010,** *66* (Pt 1), 12-21.
13. Adams, P. D.; Afonine, P. V.; Bunkoczi, G.; Chen, V. B.; Davis, I. W.; Echols, N.; Headd, J. J.; Hung, L. W.; Kapral, G. J.; Grosse-Kunstleve, R. W.; McCoy, A. J.; Moriarty, N. W.; Oeffner, R.; Read, R. J.; Richardson, D. C.; Richardson, J. S.; Terwilliger, T. C.; Zwart, P. H., PHENIX: a comprehensive Python-based system for macromolecular structure solution. *Acta Crystallogr D* **2010,** *66* (Pt 2), 213-21.
14. Humphrey, W.; Dalke, A.; Schulten, K., VMD: Visual molecular dynamics. *Journal of Molecular Graphics* **1996,** *14* (1), 33-38.
15. Webb, B.; Sali, A., Comparative Protein Structure Modeling Using MODELLER. *Curr Protoc Bioinformatics* **2016,** *54*, 5 6 1-5 6 37.
16. Olsson, M. H.; Sondergaard, C. R.; Rostkowski, M.; Jensen, J. H., PROPKA3: Consistent Treatment of Internal and Surface Residues in Empirical pKa Predictions. *J Chem Theory Comput* **2011,** *7* (2), 525-37.
17. Sondergaard, C. R.; Olsson, M. H.; Rostkowski, M.; Jensen, J. H., Improved Treatment of Ligands and Coupling Effects in Empirical Calculation and Rationalization of pKa Values. *J Chem Theory Comput* **2011,** *7* (7), 2284-95.
18. Zhang, L.; Hermans, J., Hydrophilicity of cavities in proteins. *Proteins: Structure, Function, and Genetics* **1996,** *24* (4), 433-438.
19. Gumbart, J.; Trabuco, L. G.; Schreiner, E.; Villa, E.; Schulten, K., Regulation of the protein-conducting channel by a bound ribosome. *Structure* **2009,** *17* (11), 1453-64.
20. Jo, S.; Kim, T.; Iyer, V. G.; Im, W., CHARMM-GUI: A web-based graphical user interface for CHARMM. *Journal of Computational Chemistry* **2008,** *29* (11), 1859-1865.
21. Lomize, M. A.; Lomize, A. L.; Pogozheva, I. D.; Mosberg, H. I., OPM: Orientations of Proteins in Membranes database. *Bioinformatics* **2006,** *22* (5), 623-625.
22. Phillips, J. C.; Hardy, D. J.; Maia, J. D. C.; Stone, J. E.; Ribeiro, J. V.; Bernardi, R. C.; Buch, R.; Fiorin, G.; Henin, J.; Jiang, W.; McGreevy, R.; Melo, M. C. R.; Radak, B. K.; Skeel, R. D.; Singharoy, A.; Wang, Y.; Roux, B.; Aksimentiev, A.; Luthey-Schulten, Z.; Kale, L. V.; Schulten, K.; Chipot, C.; Tajkhorshid, E., Scalable molecular dynamics on CPU and GPU architectures with NAMD. *J Chem Phys* **2020,** *153* (4), 044130.
23. Phillips, J. C.; Braun, R.; Wang, W.; Gumbart, J.; Tajkhorshid, E.; Villa, E.; Chipot, C.; Skeel, R. D.; Kale, L.; Schulten, K., Scalable molecular dynamics with NAMD. *J. Comput. Chem.* **2005,** *26* (16), 1781-802.
24. Huang, J.; Rauscher, S.; Nawrocki, G.; Ran, T.; Feig, M.; de Groot, B. L.; Grubmuller, H.; MacKerell, A. D., Jr., CHARMM36m: an improved force field for folded and intrinsically disordered proteins. *Nat Methods* **2017,** *14* (1), 71-73.
25. Klauda, J. B.; Venable, R. M.; Freites, J. A.; O'Connor, J. W.; Tobias, D. J.; Mondragon-Ramirez, C.; Vorobyov, I.; MacKerell, A. D., Jr.; Pastor, R. W., Update of the CHARMM all-atom additive force field for lipids: validation on six lipid types. *J Phys Chem B* **2010,** *114* (23), 7830-43.
26. Jorgensen, W. L.; Chandrasekhar, J.; Madura, J. D.; Impey, R. W.; Klein, M. L., Comparison of simple potential functions for simulating liquid water. *The Journal of Chemical Physics* **1983,** *79* (2), 926-935.
27. Vanommeslaeghe, K.; Hatcher, E.; Acharya, C.; Kundu, S.; Zhong, S.; Shim, J.; Darian, E.; Guvench, O.; Lopes, P.; Vorobyov, I.; Mackerell, A. D., Jr., CHARMM general

force field: A force field for drug-like molecules compatible with the CHARMM all-atom additive biological force fields. *J Comput Chem* **2010,** *31* (4), 671-90.
28. Vanommeslaeghe, K.; Raman, E. P.; MacKerell, A. D., Jr., Automation of the CHARMM General Force Field (CGenFF) II: assignment of bonded parameters and partial atomic charges. *J Chem Inf Model* **2012,** *52* (12), 3155-68.
29. Darden, T.; York, D.; Pedersen, L., Particle mesh Ewald: AnN·log(N) method for Ewald sums in large systems. *The Journal of Chemical Physics* **1993,** *98* (12), 10089-10092.
30. Ryckaert, J.-P.; Ciccotti, G.; Berendsen, H. J. C., Numerical integration of the cartesian equations of motion of a system with constraints: molecular dynamics of n-alkanes. *Journal of Computational Physics* **1977,** *23* (3), 327-341.
31. Martyna, G. J.; Tobias, D. J.; Klein, M. L., Constant pressure molecular dynamics algorithms. *The Journal of Chemical Physics* **1994,** *101* (5), 4177-4189.
32. Feller, S. E.; Zhang, Y.; Pastor, R. W.; Brooks, B. R., Constant pressure molecular dynamics simulation: The Langevin piston method. *The Journal of Chemical Physics* **1995,** *103* (11), 4613-4621.
33. Cournia, Z.; Allen, B.; Sherman, W., Relative Binding Free Energy Calculations in Drug Discovery: Recent Advances and Practical Considerations. *J. Chem. Inf. Model.* **2017,** *57* (12), 2911-2937.
34. Zwanzig, R. W., High-Temperature Equation of State by a Perturbation Method. I. Nonpolar Gases. *The Journal of Chemical Physics* **1954,** *22* (8), 1420-1426.
35. Chen, H.; Maia, J. D. C.; Radak, B. K.; Hardy, D. J.; Cai, W.; Chipot, C.; Tajkhorshid, E., Boosting Free-Energy Perturbation Calculations with GPU-Accelerated NAMD. *J. Chem. Inf. Model.* **2020,** *60* (11), 5301-5307.
36. Liu, P.; Dehez, F.; Cai, W.; Chipot, C., A Toolkit for the Analysis of Free-Energy Perturbation Calculations. *J. Chem. Theory Comput.* **2012,** *8* (8), 2606-16.
37. Bennett, C. H., Efficient estimation of free energy differences from Monte Carlo data. *Journal of Computational Physics* **1976,** *22* (2), 245-268.
38. Goddard, T. D.; Huang, C. C.; Meng, E. C.; Pettersen, E. F.; Couch, G. S.; Morris, J. H.; Ferrin, T. E., UCSF ChimeraX: Meeting modern challenges in visualization and analysis. *Protein Sci.* **2018,** *27* (1), 14-25.
39. Pettersen, E. F.; Goddard, T. D.; Huang, C. C.; Couch, G. S.; Greenblatt, D. M.; Meng, E. C.; Ferrin, T. E., UCSF Chimera--a visualization system for exploratory research and analysis. *J. Comput. Chem.* **2004,** *25* (13), 1605-12.
40. Yamakawa, T.; Ideue, E.; Iwaki, Y.; Sato, A.; Tokuyama, H.; Shimokawa, J.; Fukuyama, T., Total synthesis of tryprostatins A and B. *Tetrahedron* **2011,** *67* (35), 6547-6560.
